# SIRT2 Ablation Has No Effect on Tubulin Acetylation in Brain, Cholesterol Biosynthesis or the Progression of Huntington's Disease Phenotypes *In Vivo*

**DOI:** 10.1371/journal.pone.0034805

**Published:** 2012-04-12

**Authors:** Anna Bobrowska, Gizem Donmez, Andreas Weiss, Leonard Guarente, Gillian Bates

**Affiliations:** 1 Department of Medical and Molecular Genetics, King's College London, London, United Kingdom; 2 Paul F. Glenn Laboratory and Department of Biology, Massachusetts Institute of Technology, Cambridge, Massachusetts, United States of America; 3 Neuroscience Discovery, Novartis Institutes for BioMedical Research, Basel, Switzerland; Tokyo Medical and Dental University, Japan

## Abstract

Huntington's disease (HD) is a devastating neurodegenerative disorder for which there are no disease-modifying treatments. The molecular pathogenesis of HD is complex and many mechanisms and cellular processes have been proposed as potential sites of therapeutic intervention. However, prior to embarking on drug development initiatives, it is essential that therapeutic targets can be validated in mammalian models of HD. Previous studies in invertebrate and cell culture HD models have suggested that inhibition of SIRT2 could have beneficial consequences on disease progression. SIRT2 is a NAD^+^-dependent deacetylase that has been proposed to deacetylate α-tubulin, histone H4 K16 and to regulate cholesterol biogenesis – a pathway which is dysregulated in HD patients and HD mouse models. We have utilized mice in which SIRT2 has been reduced or ablated to further explore the function of SIRT2 and to assess whether SIRT2 loss has a beneficial impact on disease progression in the R6/2 mouse model of HD. Surprisingly we found that reduction or loss of SIRT2 had no effect on the acetylation of α-tubulin or H4K16 or on cholesterol biosynthesis in the brains of wild type mice. Equally, genetic reduction or ablation of SIRT2 had no effect on HD progression as assessed by a battery of physiological and behavioural tests. Furthermore, we observed no change in aggregate load or levels of soluble mutant huntingtin transprotein. Intriguingly, neither the constitutive genetic loss nor acute pharmacological inhibition of SIRT2 affected the expression of cholesterol biosynthesis enzymes in the context of HD. Therefore, we conclude that SIRT2 inhibition does not modify disease progression in the R6/2 mouse model of HD and SIRT2 inhibition should not be prioritised as a therapeutic option for HD.

## Introduction

Huntington's Disease (HD) is a devastating, autosomal dominant, neurodegenerative disorder, with a mean age of onset of 40 years [1]. HD symptoms are typically movement disorders, rapid weight loss, dementia and psychiatric disturbances, and the disease progresses to death over the course of 15–20 years [1]–[3]. The progressive atrophy of the cerebral cortex and basal ganglia is the most striking neuropathological change, although other brain regions are also affected with the result that HD patients can lose as much as 40% of their brain volume [4]–[6]. At the molecular level HD is caused by the expansion of a CAG tri-nucleotide repeat within exon 1 of the huntingtin gene (*HTT*), which translates into an expanded polyglutamine (polyQ) tract in the N-terminus of the huntingtin protein (HTT) [7]. Ubiquitin positive cytoplasmic aggregates and intranuclear inclusions which appear to consist of N-terminal mutant HTT (mHTT) fragments [8], are a prominent neuropathological feature. Disturbances in many cellular processes including transcriptional regulation, synaptic function, intracellular trafficking and energy homeostasis have been observed in HD [9]. Whether these contribute to or are an effect of HD pathology is currently unclear [9]. Therapeutic options currently available to HD patients are directed against primary symptoms such as chorea or depression, however, these approaches do not modify disease progression and as such, have limited benefits [3].

Histone deacetylase (HDAC) inhibition has recently emerged as an attractive therapeutic intervention for many complex diseases, including HD. There are altogether 18 mammalian HDACs, divided into four classes depending on their sequence homology to yeast enzymes. HDACs 1 through 11 are all zinc dependent enzymes, share the same reaction mechanism and belong to classes I, II and IV [10]. Sirtuins form the class III deacetylases which contain seven enzymes that are similar to the yeast Sir2, thus named SIRT1 through SIRT7 [11]. The sirtuin reaction mechanism is NAD^+^ (nicotinamide adenine dinucleotide) dependent and proceeds through the formation of an oxocarbenium-like transition species and the release of nicotinamide (NAM) [12]. As a side product of the deacetylation reaction, NAM is a pan-sirtuin inhibitor. Treatment with NAM has shown beneficial effects in a mouse model of HD [13]. However, it has also been demonstrated that depletion of SIRT1 exacerbates phenotypes in an HD mouse model [14]. Therefore, pan-inhibition of all sirtuins could have a less positive net effect than the inhibition of a specific sirtuin other than SIRT1. It is thus important to identify which sirtuin(s), when inhibited, has beneficial consequences for HD.

SIRT2 is the only member of the sirtuin family residing primarily in the cytoplasm, where it has been shown to deacetylate α-tubulin [15], [16]. Other clients of SIRT2 include histone 4 acetylated at lysine 16 (H4K16), FOXO1, FOXO3a, p53 and PEPCK1, and it has therefore been suggested that SIRT2 has a role in cell cycle progression and the regulation of gluconeogenesis as well as the stress responses to caloric restriction and cold exposure [17]–[21].

Evidence for a protective role of SIRT2 inhibition in HD came from two important studies. Firstly, genetic knock-down of *Sirt2* to 50% of normal levels prevented photoreceptor neuron degeneration in a HTT exon 1 *D. melanogaster* HD model, but did not rescue lethality [22]. In a second study, a specific SIRT2 inhibitor was demonstrated to be protective in *D. melanogaster*, *C. elegans* and primary striatal cell models of HD [23]. Although microarray profiling of HD striatal cells showed that SIRT2 inhibition did not correct the transcriptional dysregulation associated with HD, it revealed an unanticipated function for SIRT2 in cholesterol biosynthesis. Treatment of striatal cells with the SIRT2 inhibitor AK-1 resulted in a down-regulation of key enzymes in the cholesterol synthesis pathway. Further examination revealed that SIRT2 facilitates the nuclear translocation of SREBP-2 and subsequent activation of the cholesterol synthesis pathway. Consistent with this, inhibition of SIRT2 decreased nuclear SREBP-2, and consequently the expression levels of the cholesterogenic enzymes and therefore levels of cholesterol. It was proposed that the neuroprotective effect observed after treatment with SIRT2 inhibitors was due to a reduction in the high cholesterol levels observed in the HD striatal cells that had been used [23]. These findings strongly suggested that SIRT2 inhibition should modify HD progression.

Based on previous studies in worm, fly and cell culture HD models, we might expect that loss of SIRT2 would decrease aggregate load and cholesterol levels and modify HD progression in a mouse model of HD [22], [23]. To verify whether this is the case, *Sirt2* knock-out (*Sirt2*KO) mice have been crossed to the R6/2 mouse model of HD and disease progression was assessed with physiological, behavioural and molecular readouts. The R6/2 mouse has an early onset and rapid phenotype progression and at late stage disease expresses HD-related phenotypes that are extremely similar to those that develop in the genetically precise *Hdh*Q150 knock-in HD model [24]–[29]. Here we show that genetic depletion of SIRT2 does not affect disease progression in R6/2 mice. Additionally, we have observed no difference in the levels of soluble or aggregated mHTT transprotein upon SIRT2 ablation. Finally, we were unable to detect a role for SIRT2 in the cholesterol synthesis pathway, either in *Sirt2*KO mice or in mice acutely dosed with a SIRT2 inhibitor. We find no evidence for the therapeutic potential of SIRT2 modulation in a mammalian model of HD and suggest that SIRT2 inhibition should not be prioritised as a therapeutic strategy for HD.

## Results

### *Sirt2* knock-out mice do not express the SIRT2 protein

**Figure 1:**
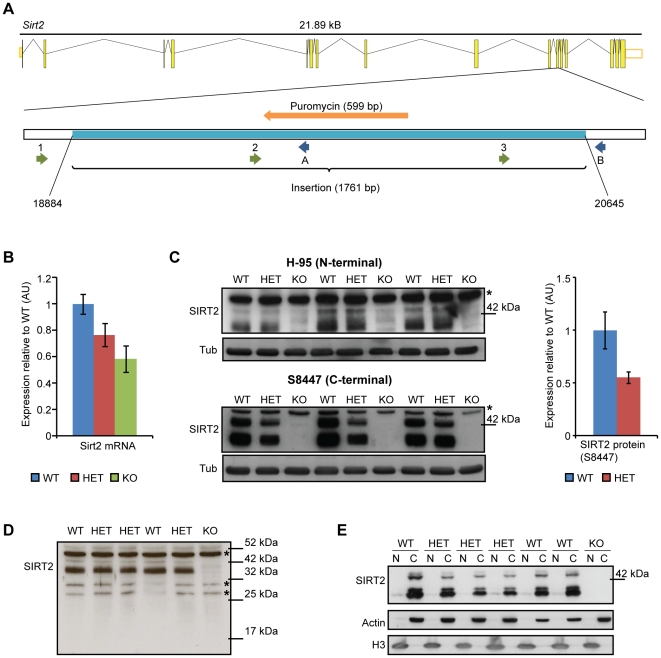
Reduction of *Sirt2* mRNA and an absence of the SIRT2 protein in *Sirt2* knock-out mice. (**A**) Exon-intron structure of the *Sirt2* gene in mouse and the location of the insertion (light blue) in exon 11 (after nucleotide 18883) in *Sirt2*KO mice. The positions of the sequencing forward and reverse primers are shown. 1-*Sirt2* forward, 2-*Sirt2* forward Seq2, 3-*Sirt2* forward Seq3, A-*Sirt2* reverse KO, B-*Sirt2* reverse WT. (**B**) Cortical *Sirt2* mRNA levels in 4 week old *Sirt2*KO (KO), *Sirt2*HET (HET) and wild type (WT) mice. Expression levels were normalised to the housekeeping genes *Atp5b* and *Canx* and expressed as fold change of WT levels ±SEM. n = 8/genotype. (**C**) Western blotting of KO, HET and WT brain lysates with SantaCruz H-95 (upper panel) and Sigma S8447 (lower panel) antibodies. The S8447 probed blot was used to quantify SIRT2 levels (both bands) between HET and WT (right panel). Values were normalised to α-tubulin (Tub) and expressed as fold change of WT ±SEM. * denotes a non-specific band. (**D**) Western blotting of KO, HET and WT brain lysates with SantaCruz H-95 antibody (long exposure) demonstrating that the *Sirt*2 disrupting mutation does not result in the production of an N-terminal fragment of SIRT2. *denotes non-specific bands. (**E**) SIRT2 is localised to the cytoplasm. Purity of fractions was determined by measuring the expression of actin (C-cytoplasm) and H3 (N-nucleus).

*Sirt2* knock-out (*Sirt2*KO) mice were generated by the targeted insertion of a puromycin resistance gene into exon 11 of the *Sirt2* locus. The insertion was sequenced and BLAST analysis confirmed that in addition to vector backbone sequences, the mutation introduced a puromycin resistance gene countersense to the *Sirt2* gene (Fig. 1A). Further analysis showed that the insertion introduces a stop codon that should result in nonsense-mediated decay of the *Sirt2* mRNA (Fig. S1).

To investigate the effects of the mutation on *Sirt2* expression, cortical *Sirt2* mRNA levels were measured by quantitative real-time PCR (qPCR) with primers binding upstream of the insertion in *Sirt2*KO, *Sirt2*HET (*Sirt2* heterozygous) and wild type (WT) mice at 4 weeks of age. *Sirt2*HET and *Sirt2*KO mice were found to express 80% and 60% of *Sirt2* mRNA levels as compared to WT respectively (Fig. 1B). To investigate the mechanism by which the insertion affects SIRT2 protein synthesis, we probed brain lysates from 4 week old mice with N- (Santa Cruz H-95) or C-terminal (Sigma S8447) anti-SIRT2 antibodies. Western blotting revealed 3 bands that correspond to the predicted molecular weight of the three SIRT2 isoforms (43, 37 and 34 kDa) [30], all of which were absent in *Sirt2*KO mice and reduced to 55% of WT levels in *Sirt2*HETs (Fig. 1C). These experiments also ruled out the possibility that the disruption of the *Sirt*2 gene resulted in the production of an N-terminal fragment of SIRT2 (Fig. 1D). Finally, the primarily cytoplasmic localisation of SIRT2 [15], [16] was not modulated by the reduction in SIRT2 levels (Fig. 1E). We have also ascertained that the reduction or depletion of SIRT2 does not affect the mRNA expression of the other *Hdacs* or *sirtuins*, or the expression of the SIRT1 protein, which shares the most sequence homology and substrate specificity with SIRT2 [20], [21], [31], [32] (Fig. S2).

### Genetic depletion of SIRT2 does not affect the acetylation of tubulin or of H4K16 and does not modify the expression of the cholesterol biosynthesis enzymes

**Figure 2:**
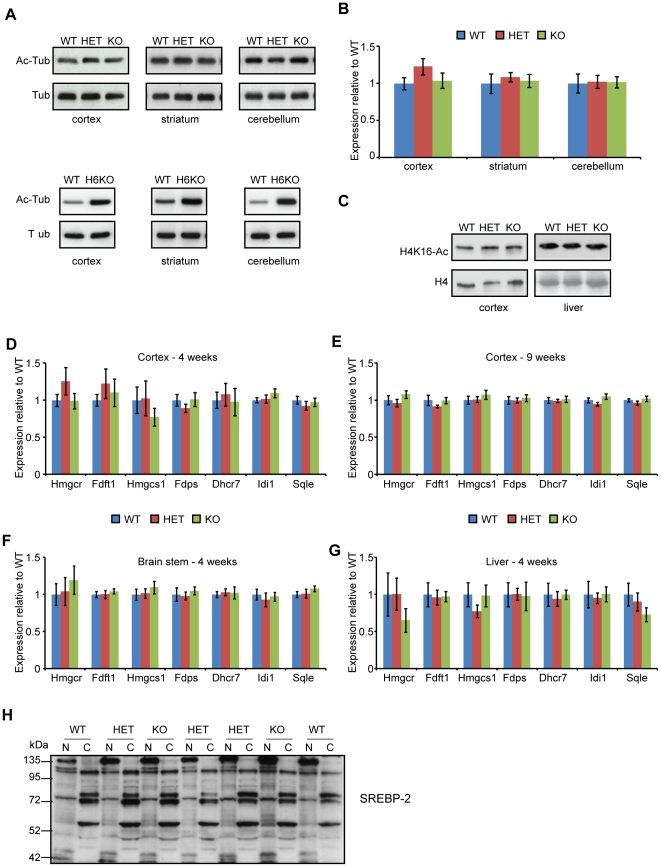
SIRT2 depletion does not affect α-tubulin or H4K16 acetylation or the expression of cholesterogenic enzymes. (**A**) Representative western blots of acetylated tubulin (Ac-Tub) at 4 weeks of age in the cortex, striatum and cerebellum of WT, *Sirt2*HET and *Sirt2*KO (upper panel) and WT and *Hdac6*KO (H6KO) mice (lower panel). (**B**) Quantification of acetylated tubulin in WT, *Sirt2*HET and *Sirt2*KO mice. Signal was normalised to the level of total tubulin (Tub) and expressed as fold change of WT ± SEM. n = 4/genotype. (**C**) Representative western blots of acetylated H4K16 (H4K16-Ac) and total H4 (H4) at 4 weeks of age in the cortex and liver of WT, HET and KO mice. n = 3/genotype (cortex) and n = 6/genotype (liver). (**D–G**) The mRNA expression levels of 7 cholesterogenic enzymes were determined by Taqman qPCR in the cortex at (**D**) 4 and (**E**) 9 weeks of age and in the (**F**) brain stem and (**G**) liver at 4 weeks of age between wild type (WT), *Sirt2*HET (HET) and *Sirt2*KO (KO) mice. n≥6/genotype. Expression was normalised to the housekeeping genes *Atp5b* (4 and 9 wk cortex and brain stem), *Canx* (4 and 9 week cortex and liver), *Gapdh* (brain stem and liver), and *ActB* (liver) and expressed as fold change of WT ± SEM. (**E**) Representative immunoblot for SREBP-2 in whole brains of 4 week old WT, HET, KO mice, performed on the same lysates as in Fig. 1D. The active form of SREBP-2 was expected to migrate at 60 kDa in the nuclear (N) fractions, the precursor of SREBP-2 was expected to migrate at 120 kDa in the cytoplasmic (C) fractions. n = 4/genotype.

The first two SIRT2 deacetylation substrates to be proposed were lysine (K) 40 of α-tubulin and K16 of histone 4 (H4) [15], [17]. We have previously shown that knock-out of another tubulin deacetylase, HDAC6, causes tubulin hyperacetylation throughout the brain [33]. To verify whether a similar effect would be observed after SIRT2 genetic depletion, western blotting was performed on cortical, striatal and cerebellar samples obtained from 4 week old WT, *Sirt2*HET and *Sirt2*KO mice. Interestingly, tubulin acetylation was not modified by SIRT2 depletion, indicating that HDAC6, not SIRT2, is the major tubulin deacetylase in the brain (Fig. 2A–B). To assess the effect of SIRT2 ablation on the levels of H4K16 acetylation, histones were enriched by acid extraction from the cortices and livers of 4 week old mice. Genotype specific differences could not be detected in the levels of acetylated H4K16 in the tissues examined (Fig. 2C).

Previous studies using mRNA microarray analysis suggested that inhibition of SIRT2 results in a decrease in the expression of enzymes that take part in cholesterol synthesis [23]. To verify whether genetic depletion of SIRT2 has an effect on cholesterol biosynthesis in the context of a mouse brain, we measured the expression of seven genes coding for cholesterogenic enzymes, chosen for analysis on the basis of previously published data [23]. Surprisingly, the expression of cholesterogenic enzymes was not modified by SIRT2 reduction or ablation in the cortex at 4 weeks of age (Fig. 2D). This effect was not masked by focusing on a specific time point, brain region or tissue type as no difference between WT, *Sirt2*HET and *Sirt2*KO mice could be detected in cortices from 9 week old mice, or brain stem and liver from mice at 4 weeks of age (Fig. 2E–G). SIRT2 had been proposed to modulate the expression of cholesterogenic enzymes by increasing the nuclear localisation of the 60 kDa active form of the transcription factor SREBP-2, the master regulator of the cholesterol biosynthesis pathway [23]. However, we have not been able to detect any genotype dependent changes in the intensity of bands detected with an antibody that recognises both the active and inactive forms of SREBP-2 (Fig. 2H). Taken together, these data suggest that the cholesterol biosynthesis pathway is unaltered in the brains of *Sirt2*KO mice.

### SIRT2 genetic ablation does not improve phenotypes in the R6/2 mouse model of HD

The potential therapeutic nature of SIRT2 inhibition in HD has not yet been tested in a complex mammalian system. To address this, the targeted *Sirt2* gene was crossed into the R6/2 mouse model of HD. The progeny, consisting of WT (n = 18), *Sirt2*HET (WT HET) (n = 21), *Sirt2*KO (WT KO) (n = 16), R6/2 (n = 18), *Sirt2*HETxR6/2 (R6/2 HET) (n = 19) and *Sirt2*KOxR6/2 (R6/2 KO) (n = 15) mice were monitored in a phenotyping study from 4 to 14 weeks of age and sacrificed at 15 weeks of age. The expression pattern of SIRT2 throughout the brain was consistent across this time frame (Fig. S3A). Equally, expression of *Sirt2* mRNA was not affected by the progression of HD-phenotypes in R6/2 mice (Fig. S3B). As the CAG repeat size is intimately linked to age of onset and mHTT toxicity [34], we have ascertained that mice were well matched for the CAG repeat number across the genotypes (R6/2 CAG = 213, R6/2 HET = 214, R6/2 KO = 212± standard deviation of 4 CAG's for each group).

**Figure 3:**
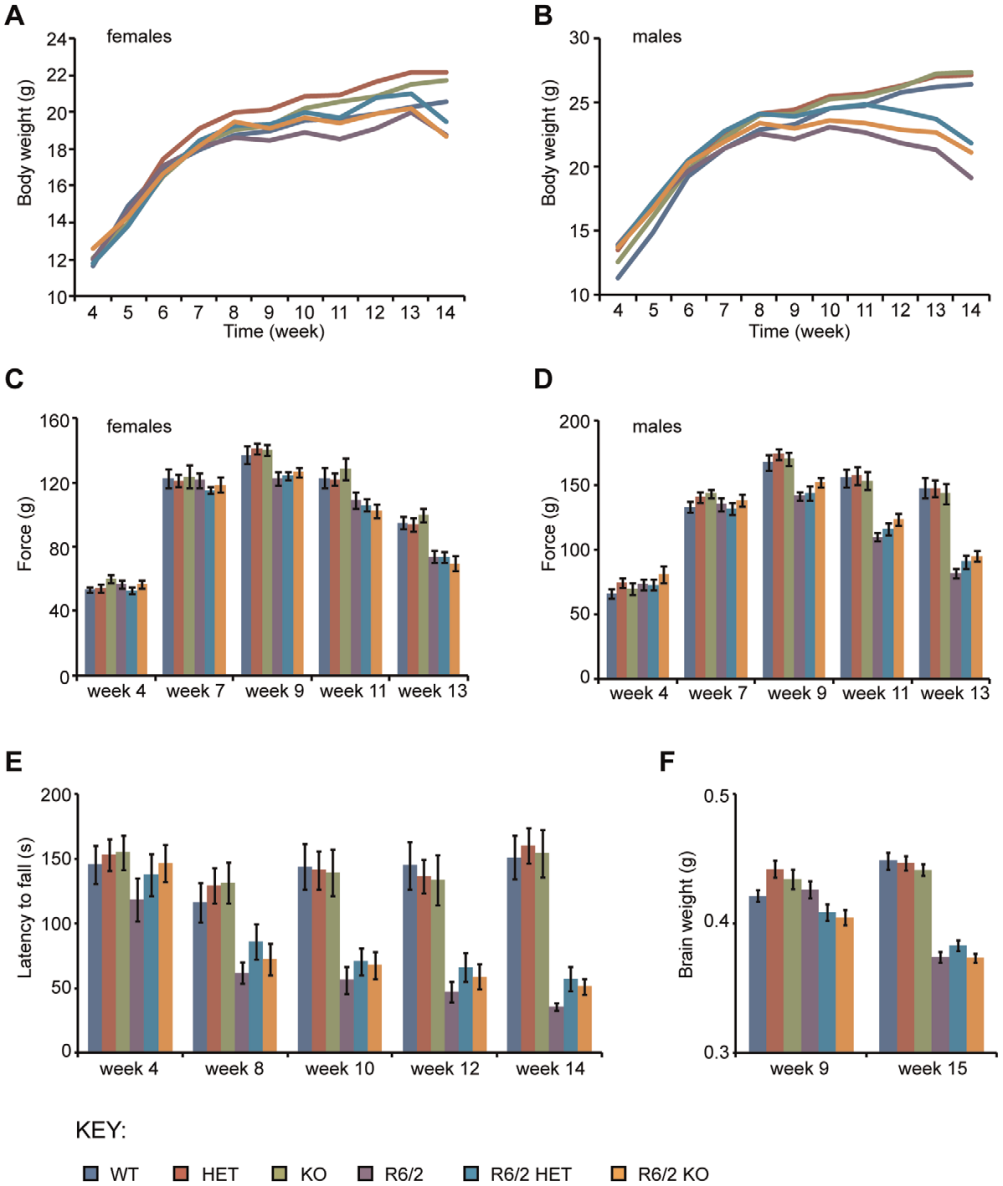
Behavioural and physiological phenotypes elicited by *Sirt2* knock-down and knock-out in R6/2 mice. (**A–B**) Mean body weight measurements in (A) female and (B) male mice. (**C–D**) Grip strength in (C) female and (D) male mice. (**E**) Rotarod performance. (**F**) Brain weight measured at 9 and 15 weeks of age. Error bars represent SEM. WT – wild type, HET – *Sirt2*HET, KO – *Sirt2*KO, R6/2 HET – *Sirt2*HETxR6/2, R6/2 KO – *Sirt2*KOxR6/2.

Mice were weighed weekly from 4 until 14 weeks of age. R6/2 mice weighed significantly less than WT mice overall (F_(1,96)_ = 10.655, *p* = 0.002) and gained weight at a significantly slower rate (F_(3,282)_ = 61.278, *p*<0.0005). Interestingly, *Sirt2*HET and *Sirt2*KO mice had significantly increased weight (F_(2,96)_ = 4.668, *p* = 0.012) (Fig. 3A–B), starting at 7 weeks of age (F_(2,96)_ = 3.152, *p* = 0.047). This phenomenon showed a trend to become significant with time (F_(6,282)_ = 2.131, *p* = 0.051). However, the *Sirt2* mutation had no overall effect on R6/2 weight loss (F_(2,96)_ = 0.087, *p* = 0.917) or on the decrease in weight of R6/2 mice over time (F_(6,282)_ = 1.902, *p* = 0.082), suggesting that if the *Sirt2* mutation modifies weight, it does so independently of HD progression.

Grip strength was measured in mice at 4 weeks of age and bi-weekly from 7 to 13 weeks of age. R6/2 mice had reduced grip strength compared to WT mice (F_(1,96)_ = 66.79, *p*<0.0005). This deficit appeared by 11 weeks of age (F_(1,96)_ = 54.462, *p*<0.0005) and deteriorated over time (F_(3,308)_ = 78.857, *p*<0.0005) (Fig. 3C–D). Grip strength was not altered in mice where SIRT2 was either reduced (*Sirt2*HET) or absent (*Sirt2*KO) overall (F_(2,96)_ = 0.654, *p* = 0.522) or with time (F_(6,308)_ = 0.355, *p* = 0.916) and there was no significant effect of the modulation of SIRT2 levels on the grip strength deterioration observed in R6/2 mice either overall (F_(2,96)_ = 0.116, *p* = 0.891), or with time (F_(6,308)_ = 0.9, *p* = 0.5) (Fig. 3C–D). These findings strongly suggest that reducing or ablating SIRT2 does not affect the grip strength in R6/2 mice.

Rotarod performance is a robust measure of motor coordination and was assessed at 4 weeks of age and bi-weekly from 8 to 14 weeks (Fig. 3E). In line with previous observations, R6/2 mice were indistinguishable from their WT littermates at 4 weeks of age (F_(1,95)_ = 2.128, *p* = 0.148) [35], but their performance deteriorated over time (F_(3,237)_ = 23.792, *p*<0.0005) (Fig. 3E). *Sirt2*HET and *Sirt2*KO mice did not perform differently from WT mice overall (F_(2,95)_ = 0.23, *p* = 0.795) or with time (F_(5,237)_ = 1.118, *p* = 0.351) and down-regulation or ablation of SIRT2 had no effect on the rotarod performance of R6/2 mice (F_(2,95)_ = 0.634, *p* = 532) or its deterioration over time (F_(5,237)_ = 0.376, *p* = 0.865) (Fig. 3E). Therefore, it was concluded that neither SIRT2 reduction nor depletion affect rotarod performance in WT or R6/2 mice.

As expected, R6/2 brains weighed significantly less than those of WT mice (F_(1,41)_ = 17.197, *p*<0.0005 at 9 weeks and F_(1,96)_ = 268.291, *p*<0.0005 at 15 weeks of age) (Fig. 3F). Genetic modulation of *Sirt2* had no effect on brain weight in WT or R6/2 mice (F_(2,41)_ = 0.734, p = 0.486 at 9 weeks and F_(2,96)_ = 1.107, *p* = 0.335 at 15 weeks).

Spontaneous motor activity was measured bi-weekly from 5 to 13 weeks of age. Data were analysed with General Linear Model repeated measures ANOVA and *p*-values are presented in Table S1. Figures S4 and S5 depict 5 min moving averages for activity and mobility (Fig. S4), and rearing and centre rearing (Fig. S5). Deficits in mobility were already apparent in R6/2 mice at 5 weeks of age, which is consistent with previous reports [36]. However, the reduction or ablation of SIRT2 had no effect on any of the parameters at any age tested, did not modify the pattern of exploration over time and did not affect the hypoactivity of R6/2 mice (Table S1 *Sirt2*KO Genotype, Time*Sirt2KO, R6/2*Sirt2KO). These data imply that depletion of SIRT2 has no effect on the progressive hypoactivity of R6/2 mice.

### SIRT2 knock-down and knock-out do not affect aggregate load or levels of soluble mHTT in R6/2 mice

Although the genetic ablation of SIRT2 had no discernible disease modifying effect on HD related behavioural and physiological phenotypes in R6/2 mice, it remained possible that SIRT2 depletion elicited molecular changes. Mutant HTT aggregates are formed in HD patients and all known mouse models and correlate with disease progression [37], [38]. To investigate whether SIRT2 genetic depletion modified levels of soluble or aggregated mHTT, Seprion ELISA, time resolved – Förster resonance energy transfer (TR-FRET) and Mesoscale Discovery (MSD) were performed on the cortex, hippocampus, brain stem and cerebellum from 4, 9 and 15 week old R6/2, R6/2 HET and R6/2 KO mice.

**Figure 4:**
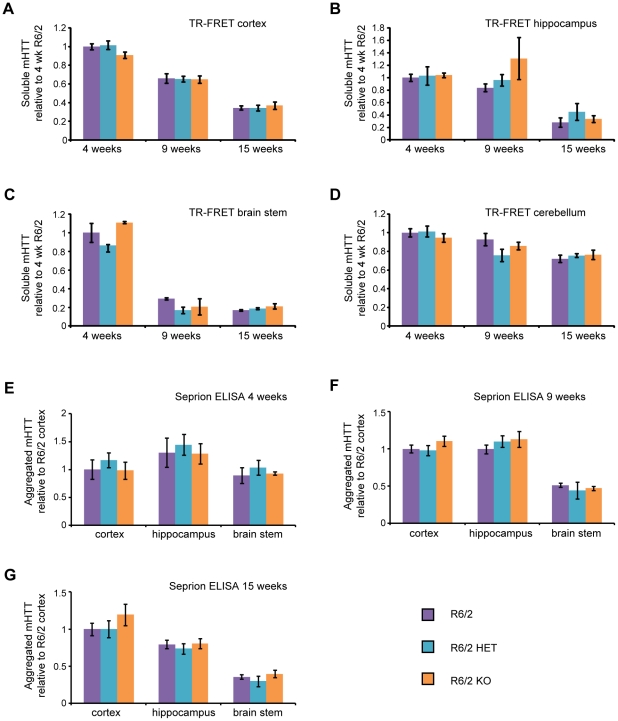
Aggregated and soluble mHTT in the brain at 4, 9 and 15 weeks of age. Levels of soluble mHTT as measured by TR-FRET with 2B7-MW1 antibodies in the (**A**) cortex, (**B**) hippocampus (**C**) brain stem and (**D**) cerebellum at 4, 9 and 15 weeks of age in R6/2, *Sirt2*HETxR6/2 (R6/2 HET) and *Sirt2*KOxR6/2 (R6/2 KO) mice. The signal for each tissue was expressed as fold change of R6/2 at 4 weeks. Signals above background were not observed for mice without the R6/2 transgene. n≥4/genotype/tissue/time point (except for R6/2 KO at 4 weeks where n = 3). Error bars represent SEM. (**E–G**) Aggregate load in the cortex, hippocampus and brain stem at (E) 4, (F) 9 and (G) 15 weeks of age as measured by Seprion ELISA with the MW8 antibody. The average signal for WT, HET and KO values was subtracted from each reading and expressed as fold change of R6/2 cortex ± SEM. n = 6/genotype(R6/2, R6/2 HET and R6/2 KO) or n = 3/genotype (WT, HET, KO).

**Figure 5:**
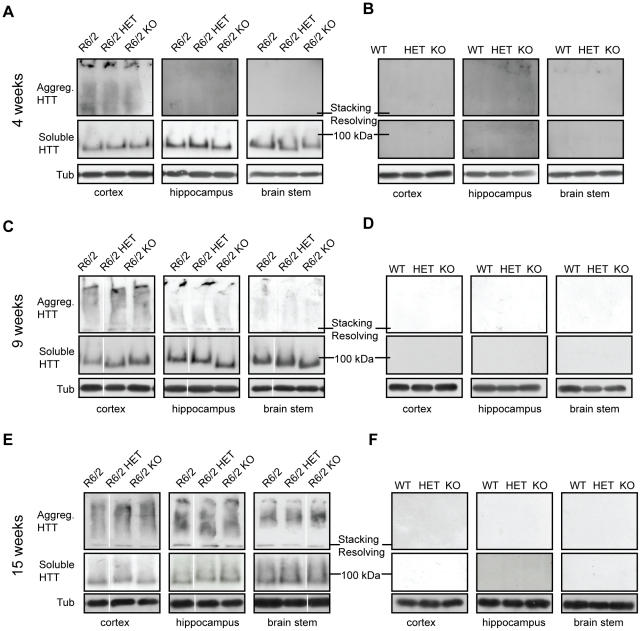
Levels of soluble mHTT in various brain regions at 4, 9 and 15 weeks of age. Representative western blots from cortex, hippocampus and brain stem of (**A–B**) 4, (**C–D**) 9 and (**E–F**) 15 week old wild type (WT), *Sirt2*HET (HET), *Sirt2*KO (KO), R6/2, *Sirt2*HETxR6/2 (R6/2 HET) and *Sirt2*KOxR6/2 (R6/2 KO) mice probed with an anti-HTT antibody (S829) and tubulin (Tub) as loading control. Both soluble mHTT transprotein and aggregates retained in the stacking gel can only be detected in mice expressing the R6/2 transgene (A, C, E). All samples were run on the same gel. White lines indicate where lanes are not contiguous.

As has been observed previously, the aggregate load in R6/2 mice was highest in the cortex and hippocampus and much lower in the brain stem (Fig. 4E–G) [26]. As expected, levels of aggregated mHTT increased and soluble mHTT decreased between 4 and 15 weeks of age in all brain regions tested (Fig. 4). Interestingly, the rate at which levels of soluble mHTT decrease with disease progression varied greatly between different brain regions (Fig. 4A–D). The Seprion ELISA did not detect any changes in the aggregate load and TR-FRET did not detect any changes in the levels of soluble mHTT in the cortex, hippocampus or brain stem at 4, 9 or 15 weeks of age between R6/2, R6/2 HET or R6/2 KO mice (Fig. 4). These results were confirmed by western blotting with an anti-HTT antibody (S829) (Fig. 5).

The results obtained from the Seprion ELISA and TR-FRET experiments were confirmed by an MSD assay (Fig. S6). Importantly, the profiles obtained for the decrease in soluble mHTT for each brain region were very similar to those seen by TR-FRET, indicating a high degree of comparability between the two methods. Although some significant differences were observed with the MSD technique, these had not been detected by TR-FRET or the Seprion ELISA. Given that the TR-FRET and MSD assays had been performed on the same lysates, it is more likely that statistically significant values were obtained by chance as a result of the large number of comparisons performed rather than reflecting a biological effect. Taken together, these data demonstrate that genetic ablation of SIRT2 does not alter the dynamics of mHTT aggregation between 4 and 15 weeks of age in brain tissues from R6/2 mice.

### Cholesterogenic enzyme dys-homeostasis is not corrected by the genetic depletion of SIRT2

**Figure 6:**
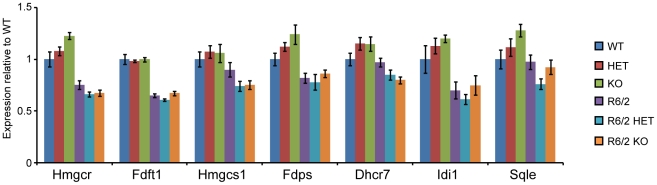
Expression of cholesterogenic enzymes at 15 week of age. The Taqman qPCR assay was used to measure the mRNA expression of the cholesterogenic enzymes in the cortex of 15 week old wild type (WT), *Sirt2*HET (HET), *Sirt2*KO (KO), R6/2, *Sirt2*HETxR6/2 (R6/2 HET) and *Sirt2*KOxR6/2 (R6/2 KO) mice. Values were normalised to the housekeeping genes *Atp5b* and *Canx* and expressed as fold change of WT ± SEM. n≥7/genotype.

SIRT2 genetic ablation had no effect on the expression of cholesterogenic enzymes in WT mice (Fig. 2D–G). However, it was possible that the effects of SIRT2 depletion would only be apparent in the context of HD pathogenesis. To verify whether this was the case, we measured the expression of cholesterogenic enzymes in WT, HET, KO, R6/2, R6/2 HET and R6/2 KO mice at 15 weeks of age. The R6/2 genotype had a statistically significant effect on the expression of all cholesterogenic enzymes (two-way ANOVA) (Fig. 6). The *Sirt2* genotype alone had no significant effect on the expression of any of the cholesterogenic enzymes. Surprisingly, however, the *Sirt2* genotype significantly modified the effect of the R6/2 genotype on the expression of *Hmgcr* (F_(2,33)_ = 6.03, p = 0.006), *Dhcr7* (F_(2,36)_ = 5.14, p = 0.011) and *Sqle* (F_(2,36)_ = 3.38, p = 0.045). Close inspection of the data revealed that the statistical significance arises from a SIRT2-related increase in the WT, and decrease in the R6/2 groups, which would be inconsistent with the proposed biological mechanism. All of the tested cholesterogenic enzymes are targets of SREBP-2, therefore, if SIRT2 ablation was causing a decrease in the nuclear shift of SREBP-2, KO mice should display lowered expression for all seven enzymes in both R6/2 and WT mice. As this was not the case, the observed statistical differences are more likely a result of biological variability than a consequence of SIRT2 depletion.

### Acute inhibition of SIRT2 has no effect on the levels of cholesterol biosynthesis enzymes in WT or R6/2 mice

**Figure 7:**
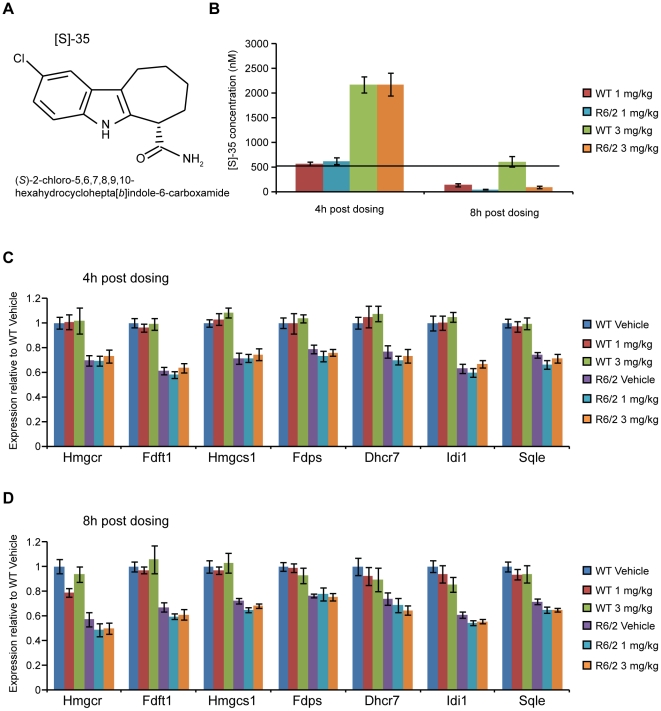
Expression of cholesterogenic enzymes in WT and R6/2 mice after an acute dose of [S]-35. (**A**) Structure of [S]-35. (**B**) Amount of [S]-35 present in brain samples from mice dosed with vehicle, 1 mg/kg or 3 mg/kg as analysed by LC/MS/MS. No compound was detected in mice dosed with vehicle alone. n≥8/genotype/dose/time point. Black line denotes the SIRT2 IC_50_ concentration of 560 nM for [S]-35 as determined in *in vitro* assays. (**C–D**) Expression of cholesterogenic enzymes in the cortex of 12 week old wild type (WT) and R6/2 mice (**C**) 4 h and (**D**) 8 h after an acute dose of the [S]-35 SIRT1/SIRT2 inhibitor or vehicle. Values were normalised to the housekeeping genes *Atp5b* and *Canx* and expressed as fold change of WT Vehicle ± SEM. n≥7/genotype/treatment group.

The lack of an effect of SIRT2 knock-out on tubulin and H4K16 acetylation, and on cholesterogenic enzyme expression could be due to compensatory mechanisms. To investigate whether this is the case, WT and R6/2 mice at 12 weeks of age were given a single low (1 mg/kg) or high (3 mg/kg) dose of the dual SIRT1/SIRT2 inhibitor, [S]-35 [39] (Fig. 7A) by oral gavage, and tissues were harvested at either 4 or 8 h post dosing. [S]-35 penetrates the blood brain barrier with brain levels peaking at 2 h post dosing (Fig. S7). The IC_50_ for SIRT2 inhibition, as measured at Cerep (assay 2582) is 560 nM and [S]-35 was found to inhibit SIRT1 with a similar IC_50_ (assay 2581). LC/MS/MS analysis of brain tissue from mice dosed with [S]-35 revealed that at 4 h post dosing, [S]-35 was still present in both WT and R6/2 mice at concentrations above the SIRT2 IC_50_ (Fig. 7B).

In line with previous reports and data from this study, expression of all cholesterogenic enzymes was diminished in R6/2 vehicle treated mice as compared to WT vehicle treated mice (Fig. 7C–D) [40]. However, neither dose of [S]-35 affected the expression of cholesterogenic enzymes at 4 or 8 h post dosing in either WT or R6/2 mice. These data strongly suggest that SIRT2 inhibition does not modulate the expression levels of cholesterol biosynthesis enzymes *in vivo*.

## Discussion

Previous studies in invertebrate and cell culture models of HD have indicated that SIRT2 inhibition alters HD-related phenotypes, particularly by modulating the expression of cholesterol biosynthesis enzymes [22], [23]. To verify whether these findings could be translated to a mouse model, we investigated the effects of SIRT2 genetic reduction and depletion on HD physiological, behavioural and molecular phenotypes in the R6/2 mouse. The data presented here show that a reduction in SIRT2 protein levels to approximately 50% or total ablation of the SIRT2 protein has no effect on the levels of acetylated tubulin, acetylated H4K16 or on the expression of enzymes involved in cholesterol biosynthesis. Additionally, we demonstrate that compensatory mechanisms have not occurred to maintain the mRNA levels of cholesterogenic enzymes as no changes in expression were observed after the acute inhibition of SIRT2. Finally, we show that the reduction or removal of the SIRT2 protein has no effect on HD progression, aggregate load or levels of soluble mHTT in the R6/2 mouse.

SIRT2 has been shown to deacetylate tubulin *in vitro* and in cell culture experiments [15]. Given the dramatic changes in tubulin acetylation in the brains of *Hdac6*KO mice [33], we were surprised to find no difference in the levels of acetylated tubulin between brains of *Sirt2*KO mice and those of wild type mice. It is possible that SIRT2 is not a *bona fide* tubulin deacetylase *in vivo*. Consistent with this, previous studies could not detect changes in the levels of acetylated tubulin in mouse embryonic fibroblasts derived from *Sirt2*KO mice [41]. Also, double mutant *Hdac6*KOx*Sirt2*KO mice are viable and do not display any overt phenotypes up to 4 weeks of age, arguing for non-redundant roles for HDAC6 and SIRT2 (unpublished observations). Therefore, it is possible that HDAC6, and not SIRT2, is the major tubulin deacetylase in the brain and that previous reports suggesting that SIRT2 is a tubulin deacetylase do not translate to the mammalian system.

Although cytoplasmic in nature, SIRT2 has been shown to regulate cell cycle progression through deacetylation of H4K16 [17]. Interestingly, we found no changes in the levels of acetylated H4K16 in brain and liver tissue from *Sirt2*KO mice. This could indicate that SIRT2 is only important for cell cycle regulation under specific conditions, as has been suggested by some, but not all, previous studies [17], [42]–[44]. Equally, in addition to its role in cell cycle progression, acetylation of H4K16 has been reported to be important for cellular processes such as maintenance of active chromatin for transcription or DNA repair [45]. Given the fundamental importance of these pathways, it is possible that compensatory mechanisms to maintain proper H4K16 acetylation levels exist and could be responsible for the inability to detect differences in H4K16 acetylation between wild type and *Sirt2*KO mice.

A partial rescue of toxicity after genetic knock-down or pharmacological inhibition of SIRT2 has been observed in *D. melanogaster* models of HD and Parkinson's disease (PD) [22], [23], [46]. However, the question of whether SIRT2 inhibition affects behaviour in the context of a mammalian model of HD had not been previously addressed. The thorough phenotypic characterisation performed in this work revealed that SIRT2 genetic depletion did not modulate rotarod performance, grip strength or spontaneous exploratory activity and had no effect on the brain weight in WT or in R6/2 mice. Interestingly, reduction and depletion of SIRT2 caused a significant increase in body weight that was observed in both WT and R6/2 mice, indicating that this effect is independent of disease progression. It is possible that the increased body weight results from the fact that SIRT2 is important for the inhibition of adipogenesis and could also be involved in maintaining cellular ATP levels [20], [47], [48]. Decreased ATP levels in *Sirt2*HET or *Sirt2*KO mice could lead to activation of the AMPK pathway, which in turn has been shown to affect multiple downstream events, including hypothalamic inhibition of POMC, activation of NPY/AgRP and an increase in food intake [49]–[51]. Further investigation will undoubtedly reveal which stages of metabolic control require SIRT2 for adequate weight maintenance.

The lack of an effect of SIRT2 loss on aggregate load or the levels of soluble mHTT was very interesting. The pharmacological inhibition of SIRT2 was found to reduce mHTT inclusion number in primary striatal neurons, but over-expression of a catalytically dead SIRT2, had little effect on mHTT inclusion number in the same model [23]. Additionally, SIRT2 knock-down rescued α-synuclein mediated toxicity in human neuroglioma cells but whilst SIRT2 inhibition led to a decrease in the number of inclusions, it caused an increase in inclusions size in the same model of PD [46]. No changes in inclusion size were observed when the same SIRT2 inhibitors were used on primary striatal HD neurons [23]. In contrast, though not a specific SIRT2 inhibitor, NAM treatment had no effect on inclusion number in R6/1 mice [13]. Consideration of the previously published findings together with the data obtained in this study suggests that not only could mechanisms of aggregate formation be quite different for α-synuclein and mHTT but also that mHTT aggregate formation and handling is dependent on the cellular and organismal context. Our findings indicate that SIRT2 inhibition would be unlikely to affect aggregate load or the levels of soluble mHTT in HD.

A role for SIRT2 in the modulation of cholesterol content was proposed after acute inhibition of SIRT2 ameliorated mHTT toxicity in worm, fly and cell culture HD models [23]. This effect was attributed to SIRT2 inhibition decreasing cholesterol synthesis via the cytoplasmic retention of SREBP-2 [23]. Surprisingly, the expression of seven enzymes of the cholesterol biosynthesis pathway was found to be unaltered in *Sirt2*KO mice. Equally, reduction and depletion of SIRT2 did not affect the expression of cholesterol biosynthesis enzymes at late stage disease in R6/2 mice. These data indicate that SIRT2 does not play a role in cholesterol synthesis in the mouse. As previous work linking SIRT2 to cholesterol synthesis was performed with inhibitors, the observed effects on the cholesterol pathway could be the result of off-target effects. Although changes in the expression of *Fdft1*, *Hgmcs1* and *Hmgcr* were also detected after over-expression of a catalytically dead SIRT2, viral transduction and over-expression of a protein can also evoke compensatory mechanisms or aberrant interactions within cells [23]. On the other hand, it was possible that the lack of changes in the expression of cholesterol biosynthesis enzymes after SIRT2 genetic depletion could be a result of compensatory mechanisms. However, our demonstration that acute SIRT2 inhibition does not affect the expression levels of cholesterol biosynthesis enzymes, verified that confounding compensatory effects were unlikely to have occurred. Our data indicate that SIRT2 does not play a role in cholesterol biosynthesis and that the previously observed findings are either a result of off-target effects of the inhibitors used or due to differences between primary cell culture and mouse models.

Previously published reports on the role of SIRT2 as a modifier of neurodegenerative disease clearly provided a rationale for conducting a phenotypic study in a mouse model of HD. Our finding that depletion of SIRT2 does not modify HD progression in R6/2 mice was surprising and the relevance of this model could be questioned. Although R6/2 mice express an N-terminal fragment of mHTT and thus lack the context of full length mutant protein, the possible involvement of SIRT2 in cholesterol biosynthesis originated from studies in cells expressing an N-terminal fragment of mHTT [23]. This indicates that the R6/2 mouse is an ideal system to extend the cellular work and study SIRT2 depletion or inhibition in the context of a mammalian brain. In addition, although knock-in models of HD are genetically precise, the striking similarity between the phenotypes that develop in the R6/2 and *Hdh*Q150 knock-in models suggests that the rate limiting step in disease onset and progression in these mice is likely to be the formation of an N-terminal mHTT fragment [25], [52]. A such, it is highly unlikely that the genetic depletion of SIRT2 would modify *Hdh*Q150 knock-in phenotypes, and as the time and resources required to perform these experiments are considerable, priorities should be directed toward the validation of more convincing therapeutic targets.

Given the devastating nature of HD and the lack of disease modifying treatments, considerable effort has been invested in the search for, and validation of, therapeutic strategies. Pan HDAC and sirtuin inhibitors such as SAHA and NAM have been shown to ameliorate disease phenotype in HD mice but can be associated with significant toxic effects. In order to divorce the toxic and beneficial effects of HDAC inhibition, considerable efforts have been made to determine which HDAC(s) and sirtuin(s) are key modifiers of disease progression. Genetic knock-down or knock-out of HDAC3, 5, 6, 7 and 9 has shown no benefit in R6/2 mice (unpublished data and [33], [53], [54]) whereas genetic knock-down of HDAC4 resulted in a marked improvement in R6/2 behavioural and molecular phenotypes (unpublished data). Similarly, whilst we have shown that a reduction in SIRT2 does not modify HD-related phenotypes in an HD mouse model, overexpression of SIRT1 has recently been shown to have beneficial consequences [14], [55]. This study demonstrates that SIRT2 does not modify disease progression in R6/2 mice and should not be prioritised as a therapeutic target for HD.

## Materials and Methods

### Ethics statement

All experimental procedures performed on mice were approved by the King's College London Ethical Review Process Committee and carried out under Home Office License 70/6545.

### Mouse husbandry

Hemizygous R6/2 mice were maintained by backcrossing R6/2 males to CBAxC57BL/6 F1 (CBF) females (B6CBAF1/OlaHsd, Harlan Olac, UK). *Sirt2* knock-out (*Sirt2*KO) mice were on C57BL/6/129Ola background and were backcrossed to CBF three times.

At 4 weeks of age, mice were weaned into cages of 5 or 6 animals. Each cage contained at least one representative of each genotype when available. Animals were housed under 12 h light/12 h dark cycle, with unlimited access to water and chow (Special Diet Services, Witham, UK). Cages were environmentally enriched as described [56]. R6/2 mice and all mice in phenotypic assessment trials were always given mash food consisting of powdered chow mixed with water from 12 weeks of age until sacrificed.

### Mouse genotyping and repeat sizing

All mice were genotyped by PCR of tail-tip DNA. R6/2 mice were genotyped and repeat sizes were determined as described previously [26]. *Sirt2* mice were genotyped with all 3 primers (Table S2) at 5 µM final concentration in a 15 µL multiplex reaction also containing 1 µL 100 ng/µL DNA, 1.5 µL 2 mM dNTP, 3 µL Phire reaction buffer (5×) and 0.2 µL Phire Hot Start II Polymerase (Finnzymes, F-122). Cycling conditions were as follows: 98°C for 30 s, (98°C for 20 s, 63°C for 20 s and 72°C for 30 min)×35, followed by 1 min at 72°C.

### Mouse phenotypic assessment

All tests were performed blind to the genotype. Mice were weighed weekly to the nearest 0.01 g. Brains were harvested with optic bulbs intact and weighed to the nearest 0.001 g immediately after cervical dislocation. Forelimb grip strength was assessed as described [56] at 4 weeks of age and then bi-weekly from 7 weeks of age. Motor coordination was assessed using an Ugo Basile 7650 rotarod, modified to accelerate from 4 to 44 rpm over 300 s, as described [56]. Spontaneous exploratory motor activity was measured bi-weekly from 5 to 13 weeks of age as described previously [36]. Mice were tested at the same time of day, with male mice always tested before female mice and cages cleaned thoroughly in between the two sexes. Data were analysed for activity (total number of beam brakes in the lower level), mobility (at least two consecutive beam breaks in the lower level), rearing (beam break in upper level) and centre rearing (beam breaks in upper level away from the cage walls).

### Sirt2 KO mutation sequencing

Tail-tip DNA from *Sirt2*KO mice and WT littermates was used for PCR with Qiagen Type-it Mutation Detection kit using 200 ng DNA in a 50 µL reaction containing 25 µL 2× Master mix, 5 µL *Sirt2* forward primer, 5 µL *Sirt2* reverse KO primer and 10 µL Q solution, with cycling conditions of: 95°C for 10 min, (95°C for 30 s, 65°C for 90 s, 72°C for 60 s)×30 and 68°C for 10 min. The PCR product was purified with the Qiaquick Gel Extraction kit according to manufacturer's instructions and cloned into TOP10 bacteria using the Invitrogen TOPO TA cloning kit according to manufacturer's instructions. Plasmid DNA was isolated with Qiaprep Spin Miniprep Kit according to manufacturer's instructions and eluted in ultra-pure water. Sequencing reactions were carried out in a 6.25 µL volume containing 200–500 ng DNA, 0.25 µL BigDye v3.1 (Applied Biosystems), 1.25 µL sequencing buffer, 0.25 µL primer (80 ng/µL) under cycling conditions of: 96°C for 2 min, (96°C for 30 s, 50°C for 20 s, 60°C for 1 min)×30. PCR products for sequencing were precipitated with 26 µL sequencing precipitation solution (120 mM C_2_H_3_O_2_Na in 95% absolute ethanol), incubated for 10 min at room temperature and centrifuged at 3,000× *g* for 20 min. The supernatant was removed, the pellet washed with 100 µL 70% ethanol, centrifuged at 3,000× *g* for 20 min, cleared of supernatant and re-suspended in 10 µL Hidi-formamide (Applied Biosystems). The sample was then denatured at 96°C for 2 min and analysed with an ABI3730 sequencer. Sequence traces were analysed with the ABI Sequencing Analysis and Vector NTI programmes. The primer sequences used are given in Table S2.

### Tissue preparation

Dissected brain regions, whole brains or peripheral tissues were snap frozen in liquid nitrogen and stored at −80°C until use.

### Nuclear/cytoplasmic fractionation

Whole brains were homogenised in 2 volumes of ice cold sucrose buffer 1 (575 mM sucrose, 25 mM KCl, 50 mM triethanolamine hydrochloride pH 7.5, 5 mM MgCl_2_, 1 mM DTT, 1 mM PMSF, 5 µM TSA, 10 mM nicotinamide, Complete protease inhibitor cocktail (Roche)) with 10 gentle pestle strokes in a Dounce homogeniser. The homogenates were centrifuged at 500× *g* for 15 min at 4°C. The supernatant (cytoplasmic fraction) was removed and stored at −80°C. The pellet was re-suspended in 1 mL of ice cold sucrose buffer 1 with an additional 10 gentle pestle strokes in the Dounce homogeniser. Two volumes of ice cold sucrose buffer 2 (2.3 M sucrose, 2.5 mM KCl, 50 mM triethanolamine hydrochloride pH 7.5, 2.5 mM MgCl_2_, 1 mM DTT, 1 mM PMSF, 5 µM TSA, 10 mM nicotinamide, Complete protease inhibitor cocktail (Roche)) were added to the homogenate and the solution was mixed until uniform. Ultracentrifuge tubes were layered with a 0.5 mL cushion of sucrose buffer 2 and the homogenate was gently layered on top. The tubes were appropriately balanced and spun in pre-cooled SW41 rotor tubes at 124,000× *g* for 1 h at 4°C. Subsequently, the supernatant was removed and the pellet re-suspended in 0.5 mL sucrose buffer 1, transferred to fresh 1.5 mL tubes and spun at 800× *g* for 15 min at 4°C. The supernatant was removed and the pellet was re-suspended in 0.5 mL buffer 1, spun at 800× *g* for 15 min at 4°C twice more before being re-suspended in 50 µL of buffer 1. The nuclear fraction was sonicated on ice for 10 s at 80 Hz. The protein concentration of both fractions was determined with the BCA assay (Thermo Scientific) for each sample.

### Acid extraction of histone proteins

Tissues were homogenised in 1 volume of acid extraction buffer (5% (v/v) Triton X-100, 3 mM DTT, 1 mM Orthovanadate, 5 mM NaF, 1 mM PMSF, 5 µM TSA, 10 mM nicotinamide, Complete protease inhibitor cocktail) using a polytron homogenising probe and centrifuged at 800× *g* for 8 min at 4°C. The supernatant was removed and the pellet washed twice by re-suspending in acid extraction buffer, and centrifuging at 800× *g* for 8 min at 4°C. The pellet was then re-suspended in 80 µL of 0.2 M HCl and incubated for 3 h at 4°C with constant shaking at 800 rpm. Samples were then centrifuged at 800× *g* for 8 min at 4°C, the supernatant collected, neutralised with 16 µL of 1 M NaOH, and the protein concentration determined for each sample with the BCA kit.

### Tissue preparation for SDS PAGE and Western blotting

Unless nuclear/cytoplasmic fractionation, acid extraction or Seprion ELISA were performed, tissue was homogenised in 1 volume of ice cold RIPA buffer (150 mM NaCl, 1% (v/v) NP-40, 0.5% (w/v) Na deoxycholate, 0.1% (w/v) SDS, 50 mM Tris-HCl pH 8.0, 1 mM 2-mercaptoethanol, 10 mM DTT, 1 mM PMSF, 5 µM TSA, 10 mM nicotinamide, Complete protease inhibitor cocktail) or TX buffer (50 mM Tris-HCl pH 7.5, 150 mM NaCl, 2 mM EDTA, 1% (v/v) Triton - X100, 1 mM PMSF, 5 µM TSA, 10 mM nicotinamide, Complete protease inhibitor cocktail) (TX was used for tubulin acetylation and RIPA buffer for all other experiments) with a polytron homogenising probe. Homogenates were sonicated on ice for 10 s at 80 Hz. Lysates were cleared by centrifugation at 16,200× *g* for 15 min at 4°C. Protein concentration was measured by BCA assay for each sample.

### SDS PAGE and Western blotting

Samples were diluted with 2× Laemmli buffer (125 mM Tris–HCl pH 6.8, 20% glycerol, 4% SDS, 0.01% (w/v) bromophenol blue) and denatured for 5 min at 95°C. Equal amounts of protein were loaded onto an SDS polyacrylamide gel alongside a size reference. Proteins were transferred onto nitrocellulose membrane at 120 V for 90 min by a submerged transfer apparatus in transfer buffer. Membranes were blocked in 5% non-fat dried milk in PBS for at least 1 h. Primary antibodies were applied in 0.02% PBS-Tween 20 (PBST) for 20 min (acetylated α-tubulin; α-tubulin; actin; H3 and H4), 1 hour (S829; SIRT2 (S8847) at room temperature or overnight (SIRT2 (sc-20966); SIRT1; SREBP-2; Acetylated H4K16; Acetylated p53; Acetylated FOXO1) at 4°C. Blots were washed three times for 5 min in 0.2% PBST and incubated with appropriate HRP coupled secondary antibody. For signal detection the enhanced chemi-luminescence (ECL) detection system, hyperfilms and Xenograph developer were used according to the manufacturers' instructions. Signals were quantified using GS-800 densitometer.

Antibodies and the dilutions at which they were used are presented in Table S3. The antibodies MW1 [57], MW8 [57], 2B7 [58] are not commercially available and were kind gifts as noted in Table S3. The S829 antibody was developed in house by the Scottish Antibody Production Unit using the same epitope as that for S830 [59].

### Coomassie staining of polyacrylamide gels

Polyacrylamide gels were stained with Coomassie solution (0.25% (w/v) Brilliant blue R-250, 50% (v/v) methanol, 10% (v/v) acetic acid) for 30 min with gentle agitation. Gels were then washed several times in ddH_2_O and three times for 10 min in de-staining solution (16.5% (v/v) methanol, 0.5% (v/v) acetic acid). Gels were dried in a vacuum gel drier.

### Seprion ELISA

For SDS-insoluble aggregate detection, 2.5% (w/v) lysates were prepared by homogenising tissue in ice cold RIPA buffer. Aggregate load was measured as previously described [26].

### TR-FRET

Time Resolved – Förster Resonance Energy Transfer (TR-FRET) experiments were performed as previously described [58].

### Mesoscale electrochemiluminescence detection of soluble and aggregated mutant huntingtin

384-well high bind MesoScale Discovery L21XB plates were coated at 4°C over night with 12 µl/well of 30 µg/ml 2B7-antibody for soluble mutant huntingtin detection or 10 µg/ml MW8-antibody for aggregated mutant huntingtin detection. Wells were washed with wash buffer (20 mM Tris, 0.9% NaCl, 0.2% Tween-20) and blocked with 35 µl Starting Block T20 (#37539 Thermo) per well. After washing, 10 µl tissue homogenate was applied. Plates were incubated for 1 h at room temperature while shaking at 450 rpm. Wells were washed and 10 µl/well detection antibody (10 µg/ml MW1-Sulfo-tag diluted 1∶10 in Starting Block T20 for soluble, 10 µg/ml MW8-Sulfo-tag diluted 1∶10 in Starting Block T20 for aggregated mutant huntingtin) were added. Plates were incubated for 1 h at room temperature while shaking at 450 rpm. After final washing steps, 2× R92TC-2 MSD Read Buffer was added and plates were analyzed with a MSD SECTOR Imager 6000 reader.

### Gene expression analysis

RNA extraction, cDNA synthesis, Taqman RT-qPCR and ΔCt analyses were performed as previously described [60]. Housekeeping genes were selected appropriate to the tissue examined. Primers and probe mixes for housekeeping genes, *Sirt4*, *Sirt6* and cholesterol pathway genes were purchased from Primer Design. Primers for analysis of expression of *Hdac1-11*, *Sirt1-3*, *Sirt5* and *Sirt7* are listed in Table S2.

### Dosing of mice with [S]-35

The [S]-35 SIRT1/SIRT2 inhibitor was synthesized at Cychem. [S]-35 was formulated in the vehicle solution (30% PEG-400, 20% Solutol HS15, 0.5% Lutrol F68 – all from BASF) immediately prior to dosing. 12 week old WT and R6/2 mice (N≥9/genotype) were weighed and given one acute dose of either vehicle, or formulated [S]-35 at 1 mg/kg or 3 mg/kg by oral gavage and dissected either 4 or 8 hours later. Each treatment group contained mice matched for age, sex and CAG repeat number.

### Measurement of brain and plasma levels of [S]-35

Blood was collected into EDTA tubes after cardiac puncture and stored on ice before centrifugation for 10 min at 4°C at 16 100× g. The plasma was removed and frozen at −20°C. Brains were removed, rinsed with saline to remove blood, weighed, and immediately frozen at −20°C. Analysis of the pharmacokinetic properties of [S]-35 was performed in 6–7 week old male C57BL/6 mice at Pharmadex and the levels of [S]-35 in the brains of WT and R6/2 mice 4 h and 8 h post dosing was performed at BioFocus.

#### Sample preparation for bioanalysis

Brain tissue was homogenized in acetonitrile∶water (3∶1, v/v) using an Precellys tissue homogenizer; the tissue-to-solvent ratio was of 1∶3 (w/v). Aliquots (25 µL of plasma or 100 µL tissue homogenate) of study samples, and corresponding controls were dispensed into 96-well plates. One hundred (100) µL of solvent containing internal standard (0.1% formic acid in acetonitrile with 200 ng/mL diclofenac as the internal standard) were added to all samples except matrix double blanks and solvent blanks. Acetonitrile∶water (75∶25 v/v) without internal standard (100 µL) was added to matrix double blanks. Samples were vortexed and centrifuged for 5 min, 100 µL of the supernatants were transferred to a new plate, diluted with 100 µL of acetonitrile∶Milli-Q water (75∶25 v/v) and submitted to LC-MS/MS analysis.

#### Bioanalytical Method

Concentrations in mouse plasma and brain were determined using LC-MS/MS assays. UPLC reverse phase separation was performed on a Waters Acquity UPLC chromatography system using a Waters Acquity UPLC BEH C18 column (50×2.1 mm, 1.7 µm); the column temperature was maintained at 40°C. The mobile phase consisted of acetonitrile containing 0.01% v/v formic acid (B) and milliQ water containing 0.01% v/v formic acid (A). A gradient elution program (flow rate 0.6 mL/min) was used with initial conditions of 5% B. These conditions were maintained for 0.2 min, followed by a linear increase to 95% B over 1 min, maintained at 95% B for 0.6 min before returning to initial conditions; the total run time was 2 min. An injection volume of 5 µL was used. The entire LC eluent was directly introduced to an electrospray (ESI) source for LC/MS/MS analysis on a Waters TQMS Xevo triple quadrupole mass spectrometer with a source temperature of 150°C and a desolvation temperature of 500°C. Positive ion mode of ESI was used for the analysis. The mass spectrometer ion optics were set in the multiple reaction monitoring (MRM) mode, and the following transitions were monitored: 263.98>218.92 for [S]-35 and 296.05>214.14 for diclofenac (the internal standard). The data was processed using QuanLynx software from Waters. Using this method, the retention time of [S]-35 and the IS were 1.18 and 1.28 min, respectively. The lower limit of assay quantitation (LLOQ) for [S]-35 was 3.8 nM for both plasma and brain.

### Statistical Analysis

Statistical analysis was performed with SPSS (three-way ANOVA, repeated measures ANOVA General Linear Model with Greenhouse-Geisser correction for non-sphericity) or Microsoft Excel (Student's *t*-test) software. For mouse phenotypic assessment, weight, rotarod and grip strength were analysed with repeated measures ANOVA and at each time point with three-way ANOVA. Brain weight was analysed by three-way ANOVA. Activity was analysed with repeated measures ANOVA at each time point separately. For western blotting, ELISA, TR-FRET and qPCR, group means were compared by Student's *t*-test, two or three-way ANOVA.

## Supporting Information

Figure S1 **Sequence of the** ***Sirt2*** **knock-out mutation.** Genomic sequence of the mouse *Sirt2* gene with the position and sequence of the mutation as present in the *Sirt2*KO mice. The inserted sequence (mutation) is highlighted in yellow. The mouse Sirt2 genomic sequence was obtained from NCBI under accession number: NC_000073.5.
(DOCX)

Figure S2 **Expression of** ***Hdacs 1-11*** **,** ***Sirt1-7*** **and SIRT1.** (**A–B**) Taqman qPCR assays measuring the mRNA expression of all known deacetylases in the cortex of 4 week old wild type (WT), *Sirt2*HET (HET) and *Sirt2*KO (KO) mice. Expression was normalised to the housekeeping genes *Atp5b* and *Canx* and expressed as fold change of WT ± SEM. n≥6/genotype. (**C**) Representative western blot probed with SIRT1 antibody showing SIRT1 expression between wild type (WT) and *Sirt2*KO (KO) brain lysates extracted from cortices of 9 week old mice in RIPA buffer. * denotes a non-specific band. Tubulin (Tub) was used as loading control. n≥3/genotype.
(TIF)

Figure S3 **SIRT2 expression is comparable between 4 and 15 weeks and not affected by HD progression.** (**A**) Representative western blot showing SIRT2 expression in different brain regions at 4 and 15 weeks of age in wild type mice. The signal for SIRT2 was normalised to total protein loaded (Coomasie stain) and expressed as fold change of rest of brain (Rb) (containing all regions except cortex (Cx), striatum (S), hippocampus (H), cerebellum (Cb) and brain stem (BS)) ± SEM. n = 4/brain region/time point. (**B**) Taqman qPCR assay measuring levels of *Sirt2* mRNA between wild type (WT) and R6/2 mice at 4 and 15 weeks of age in cortex, striatum and cerebellum. Expression was normalised to the housekeeping genes *Atp5b* and *Canx*, and expressed as fold change of WT ± SEM. n≥6/genotype.
(TIF)

Figure S4 **Activity and mobility.** Activity (single lower beam break) and mobility (consecutive lower beam breaks) were recorded for wild type (WT), *Sirt2*HET (HET), *Sirt2*KO (KO), R6/2, *Sirt2*HETxR6/2 (R6/2 HET) and *Sirt2*KOxR6/2 (R6/2 KO) mice over the course of 30 min at 5, 7, 9, 11, and 13 weeks of age. Activity parameters measurements were visualised by plotting 5 min moving averages.
(TIF)

Figure S5 **Rearing and centre rearing.** Rearing (upper beam break) and centre rearing (unsupported upper beam break) were recorded for wild type (WT), *Sirt2*HET (HET), *Sirt2*KO (KO), R6/2, *Sirt2*HETxR6/2 (R6/2 HET) and *Sirt2*KOxR6/2 (R6/2 KO) mice over the course of 30 min at 5, 7, 9, 11, and 13 weeks of age. Activity parameters measurements were visualised by plotting 5 min moving averages.
(TIF)

Figure S6 **Soluble and aggregated mHTT in the cortex, hippocampus, brain stem and cerebellum measured by MSD.** Levels of soluble (upper panels) and aggregated (lower panels) mHTT as measured by Mesoscale Discovery (MSD) in (**A**) cortex, (**B**) hippocampus, (**C**) brain stem, and (**D**) cerebellum at 4, 9 and 15 weeks of age in R6/2, *Sirt2*HETxR6/2 (R6/2 HET) and *Sirt2*KOxR6/2 (R6/2 KO) mice. Soluble mHTT was measured with 2B7-MW1 and aggregated mHTT with MW8-MW8 antibody combinations. The signal for each tissue was expressed as fold change of R6/2 at 4 weeks of age. A signal was not observed above background for mice without the R6/2 transgene. n≥4/genotype/tissue/time point (except for R6/2 KO at 4 weeks where n = 3). Error bars represent SEM. *p≤0.05, **p≤0.01 (to the R6/2 at the respective time point).
(TIF)

Figure S7 **Pharmacokinetic properties of [S]-35 in mouse brain after acute dosing.** Plasma and brain levels of [S]-35 following intravenous (IV), or oral gavage (PO) administration. Male, 6–7 weeks old C57BL/6 mice were dosed with 5 mg/kg (IV) or 10 mg/kg (PO) of [S]-35 and plasma and brains were harvested at various timepoints after compound administration. Concentration of [S]-35 in the analyte was measured by LC/MS/MS. n = 4 per timepoint, mean ± standard deviation shown.
(TIF)

Table S1 **Genetic depletion of SIRT2 does not modify R6/2 exploratory behaviour.** The *p* values displayed in Table S1 indicate whether the activity measures: activity, mobility, rearing and centre rearing are significantly influenced by R6/2 genotype, *Sirt2* genotype, Sex and the duration of the 30 min test (Time) at 5, 7, 9, 11 and 13 weeks of age. Significant *p*-values are highlighted in blue for *p*<0.05, orange for *p*<0.01 and pink for *p*<0.001.
(DOCX)

Table S2 **Summary of primer and probe sequences designed in-house.**
(DOCX)

Table S3 **Summary of all antibodies used in this study.**
(DOCX)
