# Correction: SIRT2 Ablation Has No Effect on Tubulin Acetylation in Brain, Cholesterol Biosynthesis or the Progression of Huntington’s Disease Phenotypes *In Vivo*

**DOI:** 10.1371/journal.pone.0248926

**Published:** 2021-03-25

**Authors:** Anna Bobrowska, Gizem Donmez, Andreas Weiss, Leonard Guarente, Gillian Bates

The authors wish to correct a number of errors discovered in Fig 5 of this paper [1], in which incorrect blot images were used for several panels. In this figure, results for R6/2 transgenic animals are reported in panels A, C, and E, and results for wild type (WT) animals are reported in panels B, D, F.

A summary of the errors in compiling the figure panels is as follows:

Fig 5A and 5B:

- Blots showing Aggregated mHTT in hippocampus of R6/2 mice (Fig 5A), Soluble mHTT in hippocampus of WT mice (Fig 5B), and Aggregated mHTT in hippocampus for WT mice (Fig 5B), are all incorrectly taken from a blot for the soluble fraction of cortex tissue. The Aggregated mHTT panel for R6/2 hippocampus in Fig 5A and the Soluble mHTT panel for WT hippocampus in Fig 5B are duplicate images.
- The Aggregated mHTT panels for cortex in Fig 5A and 5B, and the Soluble mHTT panel for WT cortex in Fig 5B are incorrectly taken from a blot of hippocampus samples. The Aggregated mHTT panel and the Soluble mHTT panel for WT cortex (Fig 5B) contain a region of overlap.
- With the exception of the tubulin loading controls for R6/2 brain stem (bottom right panel, Fig 5A), all tubulin loading control panels in Fig 5A and 5B are incorrect, and do not correspond to the genotype lane labels in the figure, having been taken from the incorrect lanes of their respective blots.
- The soluble mHTT and tubulin panels for R6/2 hippocampus (Fig 5A) do not report data obtained from samples from the same animals.

Fig 5C:

- In the Aggregated mHTT and tubulin panels for R6/2 cortex (Fig 5C), and in the soluble mHTT panel for R6/2 hippocampus (Fig 5C), bands were taken from the incorrect lanes of their respective blots and do not correspond to the genotype lane labels in the figure.
- In the brain stem panels of R6/2 animals (Fig 5C), bands were taken from the incorrect lanes of the blots and do not correspond to the genotype labelling of the lanes in the figure. The Aggregated mHTT and tubulin samples were separated on a different gel than the soluble mHTT fraction, though the experiments were run at the same time.
- The data shown in Aggregated mHTT, Soluble mHTT, and tubulin control panels were not all obtained using samples from the same animals for each tissue type in R6/2 animals (Fig 5C).

Fig 5E and 5F:

- The tubulin loading control panel for cortex of R6/2 animals (Fig 5E) is incorrectly taken from a blot showing hippocampus tissue.
- The Aggregated mHTT panels for cortex, hippocampus, and brainstem in Fig 5E, the tubulin panel for cortex, hippocampus and brain stem in Fig 5F, and the soluble mHTT panel for hippocampus in Fig 5F are each flipped horizontally and therefore the lane labels are incorrect in the figure.
- Incorrect data, corresponding to different genotypes, are shown in the tubulin panel for brain stem in Fig 5E. The tubulin and Soluble mHTT panels for brain stem in Fig 5E were taken from a different gel than the Aggregated mHTT panel for brain stem.
- The data shown in Aggregated mHTT, Soluble mHTT, and tubulin panels were not all obtained using samples from the same animals within each tissue type of R6/2 and WT animals (Fig 5E and 5F).

The authors provide a revised Fig 5 with data from the original experiments to correct the above errors, and the legend has been updated to describe the results more accurately. In the revised Fig 5, some panels are replaced so that the R6/2 and WT samples shown at each time point are taken from the same gels. Additionally, some panels are replaced to ensure that all panels (Aggregated mHTT, Soluble mHTT, and tubulin) for a given tissue are derived from the same sample animals. However, in Fig 5A, 5C and 5E representing results from R6/2 animals, data shown for the different tissues (cortex, hippocampus, and brain stem) are not in all cases obtained from the same animals across the tissues. Black lines indicate image splicing, where data from the same blot were rearranged when preparing the figure.

To prepare the revised Fig 5, the original autorads were rescanned, as the original scans are no longer available. Due to the change in brightness caused by rescanning, the revised Fig 5 has been prepared using mostly rescanned panels, including the ones that were correctly placed in the original figure. In the revised Fig 5 panels A-D, the Aggregated mHTT and Soluble mHTT panels for the WT samples (Fig 5B and 5D) and the Aggregated mHTT for the R6/2 samples (Fig 5A and 5C) are taken from autorads with a higher exposure than those used for the Soluble mHTT fraction in R6/2 mice (Fig 5A and 5C), to show that there is no signal for the Soluble mHTT and Aggregated mHTT levels in WT mice.

Please see the complete, correct Fig 5 and updated caption here.

**Fig 5:**
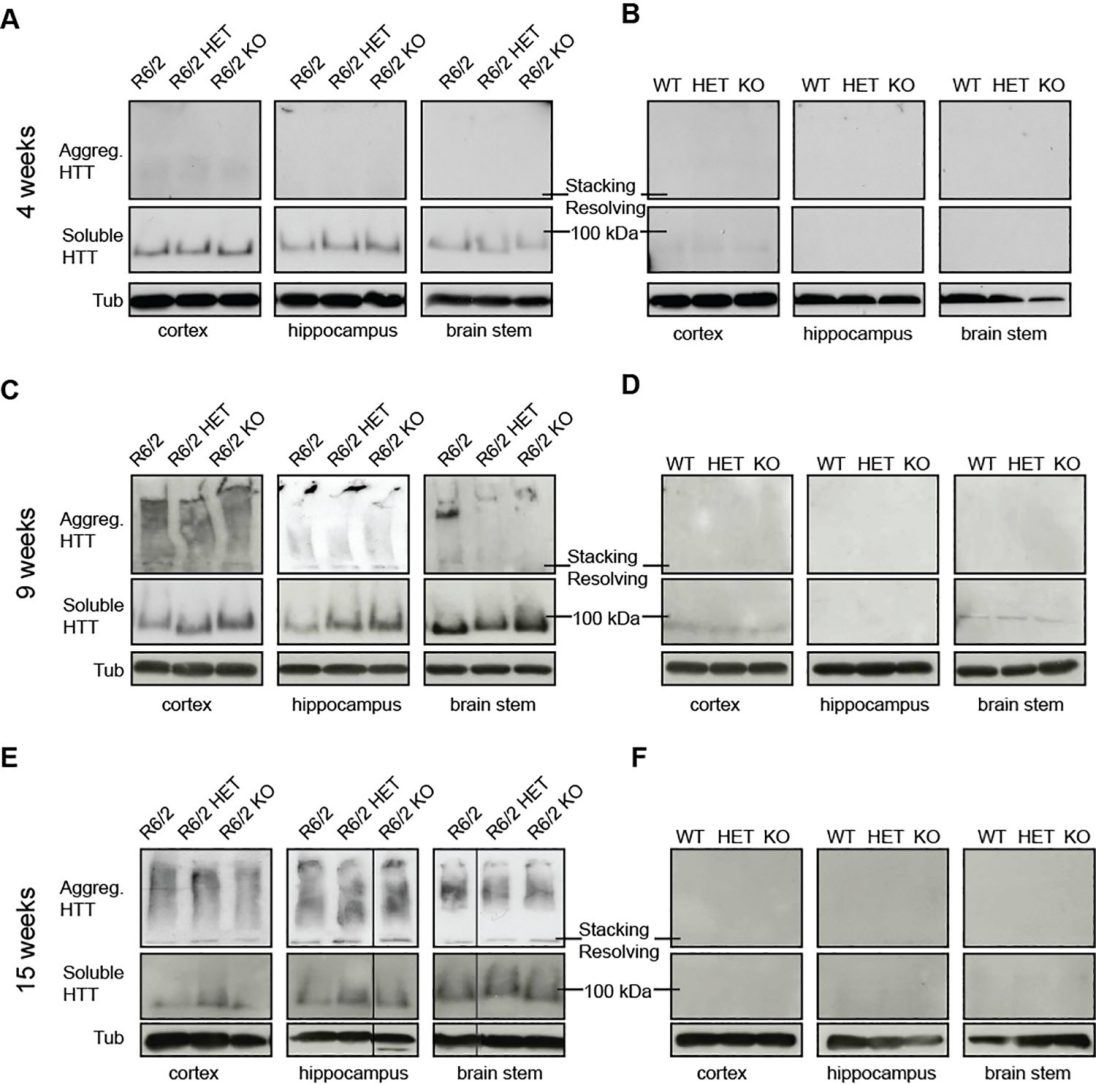
Levels of soluble mHTT in various brain regions at 4, 9 and 15 weeks of age. Representative western blots from cortex, hippocampus and brain stem of (**A–B**) 4, (**C–D**) 9 and (**E–F**) 15 week old wild type (WT), *Sirt2*HET (HET), *Sirt2*KO (KO), R6/2, *Sirt2*HETxR6/2 (R6/2 HET) and *Sirt2*KOxR6/2 (R6/2 KO) mice probed with an anti-mHTT antibody (S829) and tubulin (Tub) as loading control. Faint signal was visible in panels B and D upon longer exposure; this is non-specific as these data were obtained using samples from WT animals. All samples were run on the same gel. Black lines indicate where lanes are not contiguous.

In addition, clarifications are provided on aspects of the Results section:

The aim of the study was to compare levels of aggregated and soluble mHTT between the genotypes at different ages within each tissue. Statistical analyses were performed by comparing results across genotypes for each timepoint/tissue for the Seprion ELISA, TR-FRET and MSD data generated in this study. Where these comparisons were significant at p<0.05, this is presented in the relevant figure. In Fig 4, no comparisons were found to be significant at p<0.05.

Based on the results in the corrected Fig 5 and the data presented in Fig 4, the description of the results in Figs 4 and 5 needs to be updated. In the "SIRT2 knock-down and knock-out do not affect aggregate load or levels of soluble mHTT in R6/2 mice" subsection of the Results, the second paragraph should read as follows:

The levels of soluble and aggregated HTT between brain regions and across ages showed a similar pattern to that previously reported (Fig 4, S6 Fig) [26]. Comparing data across SIRT2 genotypes in samples carrying the transgene, the Seprion ELISA did not detect any changes in Aggregated mHTT and TR-FRET did not detect any significant changes in the levels of soluble mHTT in the cortex, hippocampus or brain stem at 4, 9 or 15 weeks of age (Fig 4, compare R6/2, R6/2 HET, R6/2 KO within each panel). These results were confirmed by western blotting with an anti-mHTT antibody (S829) (Fig 5).

Additional methodological information is also provided about the control data for Figs 1C, 1E, 2A, 2C, S2C and S3A:

The tubulin loading controls in Figs 1C and S2C were not run on the same gels used for the SIRT2 blots, as these two proteins are close in molecular mass and difficult to resolve. The gels used for the SIRT2 and tubulin blots were cast simultaneously from the same solution and equal volumes of the same protein samples were loaded onto each gel. Similarly, for experiments investigating acetylation of tubulin and H4 (Fig 2A and 2C cortex panels), control blots were obtained by running equal volumes of the samples on parallel gels as described above. For the liver panels in Fig 2C, the loading control shown is Ponceau S staining of the extracted histone proteins on the same membrane. The transfer membrane was stained immediately after the transfer for about 30 seconds with Ponceau S solution and then washed with PBS buffer, until protein bands were clearly distinguishable from the background, at which point the membrane was scanned. Ponceau S staining was removed by one wash with PBS buffer and during the blocking step. For S3A Fig, equal volumes of the same samples were run on parallel gels and one was used for a Coomassie stain. Each western blot experiment included one replicate per brain region per time point, and four separate biological samples (each on two parallel gels) were tested to confirm the reliability of the results.

The samples for the SIRT2 blot (Fig 1E) were all run on the same gel. The samples for the H3/actin blots were run on two gels and these images were cut and put together so that each H3/actin sample corresponds to the same sample used for SIRT2. Splicing together of lanes from the original H3/actin blots was carried out from the two underlying blots provided for Fig 1E H3/actin in S1 File as follows:

- Fig 1E H3/actin lanes 1–8 are from lanes 1–4 and 7–10 of the left blot;
- Fig 1E H3/actin lanes 9–12 are from lanes 1–4 of the right blot;
- Fig 1E H3/actin lanes 13–14 are from lanes 5–6 of the left blot.

Splicing together of these lanes was not clearly indicated. The authors provide a corrected Fig 1 here. Please see the complete, correct Fig 1 and updated caption.

**Fig 1:**
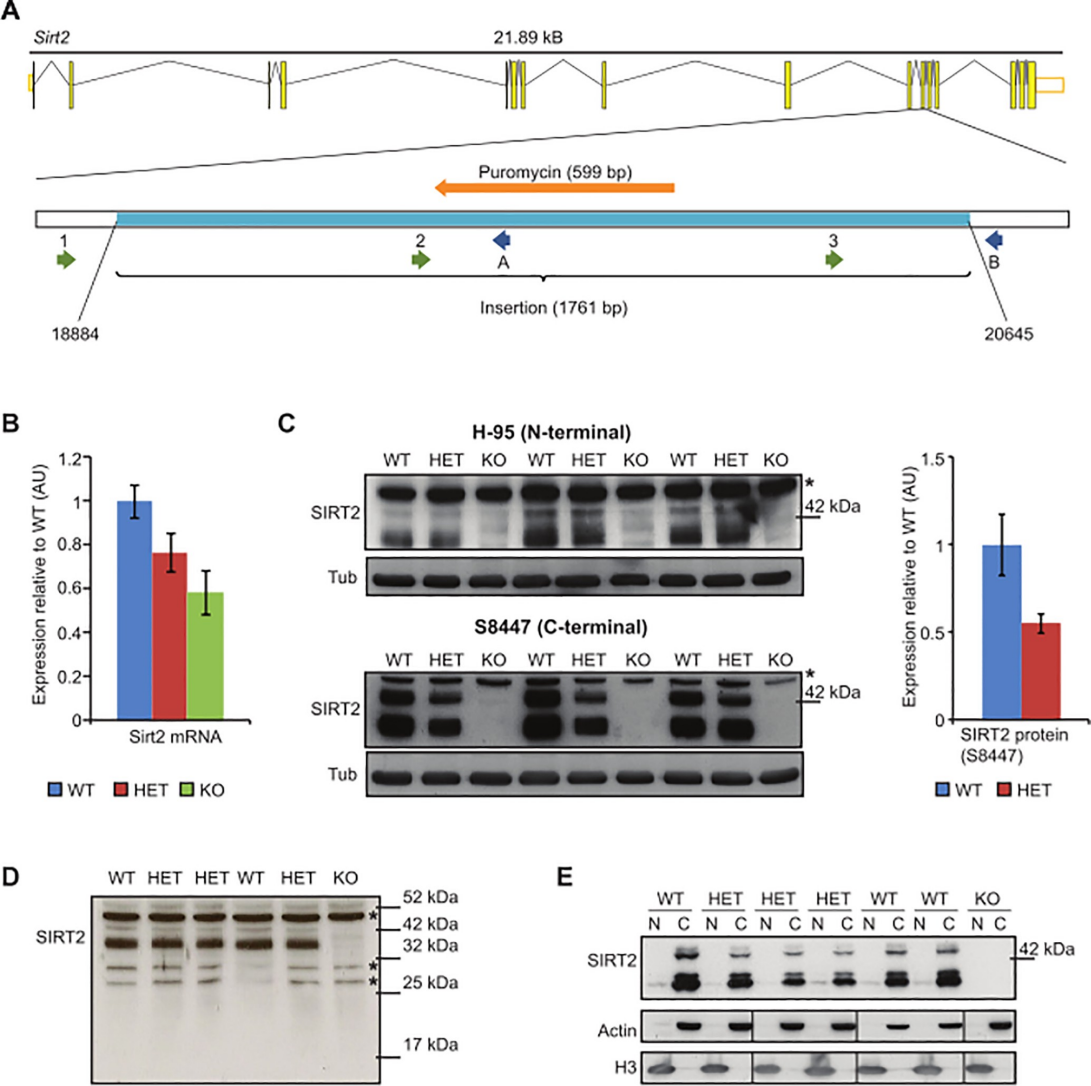
Reduction of *Sirt2* mRNA and an absence of the SIRT2 protein in *Sirt2* knock-out mice. (**A**) Exon-intron structure of the *Sirt2* gene in mouse and the location of the insertion (light blue) in exon 11 (after nucleotide 18883) in *Sirt2*KO mice. The positions of the sequencing forward and reverse primers are shown. 1-*Sirt2* forward, 2-*Sirt2* forward Seq2, 3-*Sirt2* forward Seq3, A-*Sirt2* reverse KO, B-*Sirt2* reverse WT. (**B**) Cortical *Sirt2* mRNA levels in 4 week old *Sirt2*KO (KO), *Sirt2*HET (HET) and wild type (WT) mice. Expression levels were normalised to the housekeeping genes *Atp5b* and *Canx* and expressed as fold change of WT levels ±SEM. n  =  8/genotype. (**C**) Western blotting of KO, HET and WT brain lysates with SantaCruz H-95 (upper panel) and Sigma S8447 (lower panel) antibodies. The S8447 probed blot was used to quantify SIRT2 levels (both bands) between HET and WT (right panel). Values were normalised to α-tubulin (Tub) and expressed as fold change of WT ±SEM. * denotes a non-specific band. (**D**) Western blotting of KO, HET and WT brain lysates with SantaCruz H-95 antibody (long exposure) demonstrating that the *Sirt*2 disrupting mutation does not result in the production of an N-terminal fragment of SIRT2. *denotes non-specific bands. (**E**) SIRT2 is localised to the cytoplasm. Purity of fractions was determined by measuring the expression of actin (C-cytoplasm) and H3 (N-nucleus). Black lines indicate splicing together of non-adjacent lanes or separate gels.

There is an error in the caption for Fig 2. Information pertaining to panel H was mislabelled as information pertaining to panel E. The labelling of part 2C is also amended to clarify that the loading control for the histone acetylation experiment in liver is Ponceau S stain. Please see the complete, correct Fig 2 caption here.

**Fig 2:**
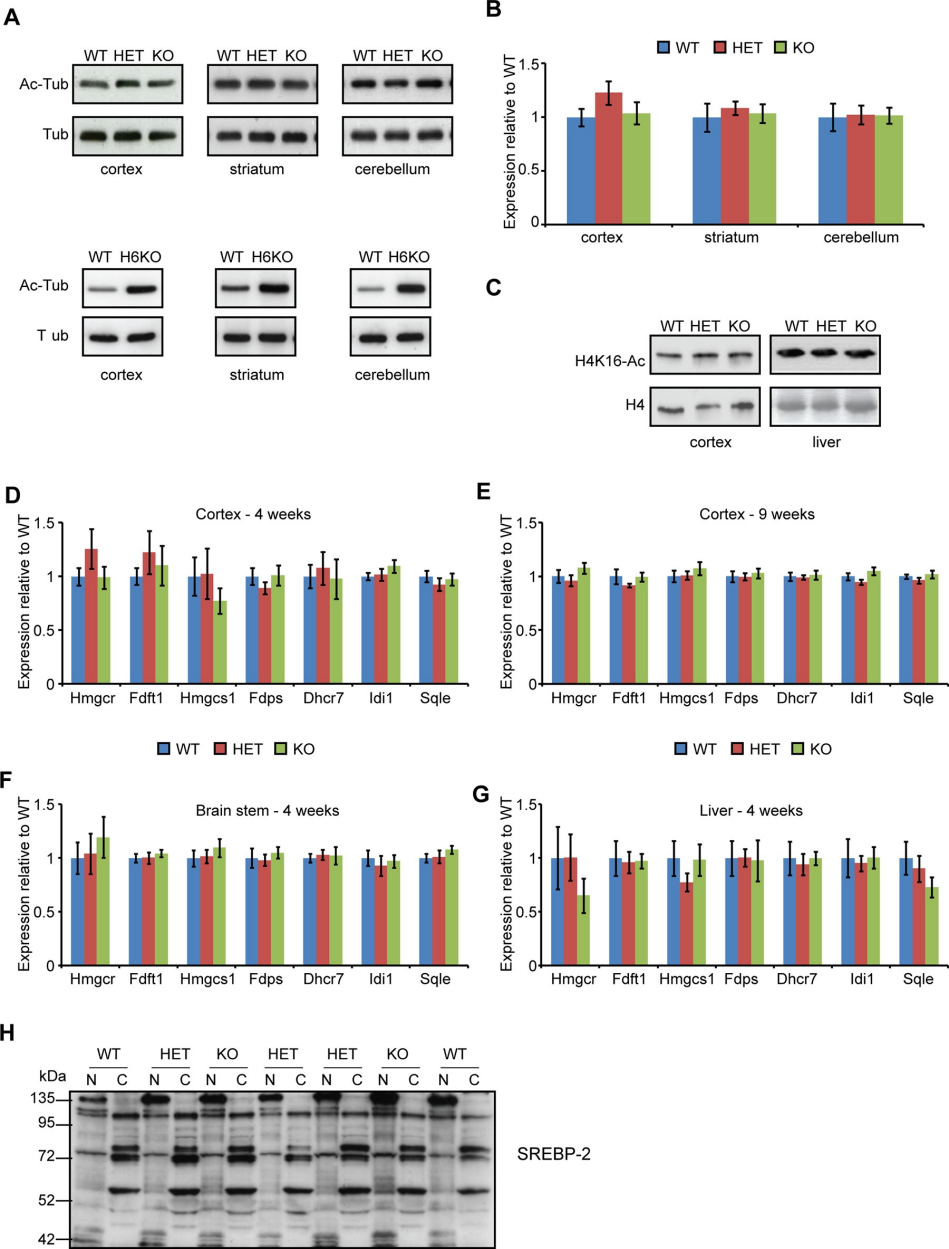
SIRT2 depletion does not affect α-tubulin or H4K16 acetylation or the expression of cholesterogenic enzymes. (**A**) Representative western blots of acetylated tubulin (Ac-Tub) at 4 weeks of age in the cortex, striatum and cerebellum of WT, *Sirt2*HET and *Sirt2*KO (upper panel) and WT and *Hdac6*KO (H6KO) mice (lower panel). (**B**) Quantification of acetylated tubulin in WT, *Sirt2*HET and *Sirt2*KO mice. Signal was normalised to the level of total tubulin (Tub) and expressed as fold change of WT ± SEM. n  =  4/genotype. (**C**) Representative western blots of acetylated H4K16 (H4K16-Ac) at 4 weeks of age in the cortex and liver of WT, HET and KO mice. n  =  3/genotype, loading control total H4 (cortex) and n  =  6/genotype, loading control Ponceau S stain of extracted histone protein, assumed to be H4 based on molecular weight and protein abundance (liver). (**D–G**) The mRNA expression levels of 7 cholesterogenic enzymes were determined by Taqman qPCR in the cortex at (**D**) 4 and (**E**) 9 weeks of age and in the (**F**) brain stem and (**G**) liver at 4 weeks of age between wild type (WT), *Sirt2*HET (HET) and *Sirt2*KO (KO) mice. n≥6/genotype. Expression was normalised to the housekeeping genes *Atp5b* (4 and 9 wk cortex and brain stem), *Canx* (4 and 9 week cortex and liver), *Gapdh* (brain stem and liver), and *ActB* (liver) and expressed as fold change of WT ± SEM. (**H**) Representative immunoblot for SREBP-2 in whole brains of 4 week old WT, HET, KO mice, performed on the same lysates as in Fig 1D. The active form of SREBP-2 was expected to migrate at 60 kDa in the nuclear (N) fractions, the precursor of SREBP-2 was expected to migrate at 120 kDa in the cytoplasmic (C) fractions. n  =  4/genotype.

The scanned autorads of the blots underlying all original figures in the article and the revised Fig 5, including those obtained using different exposures, are provided as Supporting Information accompanying this notice (S1–S4 Files), along with an illustration of the assembly of panels A-F of the revised Fig 5 (S5 File) from the uncropped blots. The loading controls for Fig 5 were all run on the same gels as used for the experimental blots, and membranes were cut for different antibody staining.

## Supporting information

S1 File Underlying Blots Fig 1_2_S2_S3 (ZIP)

S2 File Underlying Blots Fig 5A and 5B. (ZIP)

S3 File Underlying Blots Fig 5C and 5D. (ZIP)

S4 File Underlying blots Fig 5E and 5F. (ZIP)

S5 File Revised Fig 5A–5F Assembly (PPTX)
