# Supplementary material for: SIRT2 Ablation Has No Effect on Tubulin Acetylation in Brain, Cholesterol Biosynthesis or the Progression of Huntington's Disease Phenotypes In Vivo
Source: PLoS One. 2012 Apr 12;7(4):e34805. doi: 10.1371/journal.pone.0034805 (PMC3325254; doi:10.1371/journal.pone.0034805)
Supplement: Figure S1 — Sequence of the Sirt2 knock-out mutation. Genomic sequence of the mouse Sirt2 gene with the position and sequence of the mutation as present in the Sirt2KO mice. The inserted sequence (mutation) is highlighted in yellow. The mouse Sirt2 genomic sequence was obtained from NCBI under accession number: NC_000073.5. (DOCX) [file pone.0034805.s001.docx]

Genomic sequence of the mouse *Sirt2* gene with the position and sequence of the mutation as present in the *Sirt2*KO mice. The inserted sequence (mutation) is highlighted in yellow. The mouse Sirt2 genomic sequence was obtained from NCBI under accession number: NC_000073.5.

**1 TTCTTGTTTC CGCTGCCGTC ACGGGACAGA GCAGTCGGTG ACAGTCCCGA**

**51 GGGCCCCCAC CCCGTTCCCA TGGCCGAGCC GGACCGTGAG TTAGGGGCCC**

**101 TGTTGGGAGG GTGGGAGGGT CGGGCCGCGC CGGGGCCTGG CTTTGGGCTG**

**151 CAGGCCCCGG GGCCCGAGGG TAGGCTCGGT GGGGCGGGAG ACAGGATCTA**

**201 CCTGAAGGGA GTGGGATTCG GAGGGGTGTT GCTAAGTGGT CCGTATTACT**

**251 AGGTTGGTTG GTTTCTGGAT TGTGAAATGT AAGGGTCCGA TTGTCAAGTT**

**301 CTTAATGATC GCATTTCTCG TACTTTGGCT GCTGAGGACC CTCGTTGCTA**

**351 AGGACCAAAG TTACCACTAT CCCGGTTGCT AAGGTTGTCT GTTGTTAAGG**

**401 GCAACTGTAG GATGGGTTCC GGGGACCCCT GTGGCCCCTT ACTATCTCAT**

**451 TCCTTCAGTT CTTGTGCCTC TCCTAAACCT CCTACCTTCA CTGCATCTCC**

**501 TCTCCCACAC TCAACTCTCA GCAGAAGTAT CCCTACTTAC GTCTCTTGAA**

**551 AAACTTAGCC CTCCCTCATT CTCTCGACTC GCACATGCTA CCATATCCTG**

**601 CTTAACTCTG AAACCTTCCT TCAGTCCAGT CTCGCCTGCT TTCTCCTCCT**

**651 AAAGATTCCC CTCCTCAGCC TTTTCACGTC ACACATACCT CCACTCTAAA**

**701 ATATGCCTTT TCCCATCTCC GATAGTTTTA CTACCATCCC TGACTCTGAA**

**751 ATAATTAGGT TGGTGGGTCC TCTTGTCCTC CACCATTGTG AGCCCTTCTG**

**801 CTTAACTCTT TCCCCGCACT GTAACCACGT CTGCTCCCCT CCTCCCCAGC**

**851 CTCTGACCCT CTGGAGACCC AGGCAGGGAA GGTGCAGGAG GCTCAGGTGA**

**901 GATGGGGAAG GGGTGGAGGG AAGGAGAGAC TTGGTTCCAC GTGCCGCACT**

**951 GGGGGGAGGT GGCAAGGGAA GGCTTTTACC CAAGGGCCCT TGGCTTATGG**

**1001 CCCTGATCCC CAAGCCCATC TGGGGATTAG TACAATCTCC TGGGAGAATG**

**1051 GTGGCTTCTT GGGACTGTCA AGGAAAATGT ATAGCCTCAA CCCCTGGTTC**

**1101 CCTTCCATAT CGTCCTCGTT TCCTACTGTT GCCCATCCCA TCCTCAGCCC**

**1151 CTTCTGAACC TCATTTGTGC TGGACCTGAA AGAGACGGTT TTCCAGTTCA**

**1201 ATCCGCAAAC TCAGATCCTA TGAGGTGGGG GAAAGGACCC TATAGGGCAC**

**1251 GTTATAAAAG TATCGAAGCT GGGTGTGGTG CCAGCCCCCT GTAATGCCAG**

**1301 CACATGGGGG GGGGCAGGGG AGGGGGGAAG TGAGGTAGGA GGATGGAGAA**

**1351 AGGAGAGGCA GCTTGGGCCA TATGGTGAGA CCCAGTCTCA AAAAAGGAGG**

**1401 GAGGAAGGGA GAGAGCATAC CCCTGGAGTA CCGGGCAAAC TCTGTGGGTC**

**1451 AGAGATTATA GAAGATTCTA GCCAAACTCT CCCCTGGTGG AACCCACAAG**

**1501 CTGATCTAGG AACAAGACCA AGATATAGTC ACAGTGTCGT ATAAGTGAAG**

**1551 TGATAAGGGA TTTTCTTAAA TGAGATCGGA GGTGGTGTTC CATGCAGGGT**

**1601 ACAGGCCTGA GAGCACGAAG GGATAGTAGA TGTTTCAGTA GTTGGGTTGC**

**1651 TGGCGTGGTC ATTTATTGAT ACTCTGCCTG AATTTTAATG TACATGACTT**

**1701 CATTTAATCT TTGCAGCATC CCAGTGAAGG GAAGGCCTCT CATGGCTGTG**

**1751 TTACAGATGA GCAGCTTACT TGGTGAAAAC TGTTAACCCC TGTGTTCTTT**

**1801 GGGTAGAAAG GAAAGGGCTT GAAACTTATG GGGAAATGGG CTCCGAAAGA**

**1851 TGCTGCAGCT TCAGTGGAGT GTAGATGAGC CATCTCAAAG ATGTTTGAAG**

**1901 ACACGTGCCA AGGCCTCCGG TTCCCCCAGA GGCCAGGTTG TCCTCATATA**

**1951 CTCGAGGAGT GGTTCTCAAT CTTCCTAATG CTGCAACCTT TTAATGTAGC**

**2001 TCCAACGGTG TGGTGTCCCT CCAACCATAA AATTATTTTG TTACTACTTC**

**2051 ATAACTGTCC TTTTGCTACT GTTATGAACT ATAATGTAAA TATCAGATAT**

**2101 GTAGGATATC TGATATGGGA CCCCCCCCCC AAGAGAGGGT CGCTTGATCC**

**2151 CTAAAGGGAT TGTGTCCCAC AGGTTGGGAA ACAGTGTACT GGACCATGCC**

**2201 TACAACCACT GAATGCCAGC ATGCTCATCC TATGAGACCA CACTTCGAAG**

**2251 GGAGGCAGGT GCAGAAGCTG TGGTCAACAA TACCCCCATA AGAGCTGTGA**

**2301 CAGCCTGCTG TGGGTCTCTA GTCTCTTACT TGTCTGGCTC CCAAATGAAG**

**2351 GCTTAATGCC AGACTAAAAA AGGGTTTCAC AGTCAAAAGG GGTTCTCAGT**

**2401 GGCCAAGAAG ATGTGGGGTG TCAATTGCAG CATTATCCAG ATGGGGCAAC**

**2451 GCTAGCAGCA GCCTCAGTGG CTGTCAGGAG AGGGAGTCAA TGAACCACGG**

**2501 TTCATCATAC AATGTGTTAC TATGGAGCCA CAATAAAGAA TGAGGTGGGG**

**2551 GCTGGAGAGA TGGCTCAGTG GTTAAGAATT CTATTCCAGA GAACCTGAGT**

**2601 TCCATGTACC AGGCGCTCAC AAGTGACTAT AACTCCAGTC CCAGGGGATC**

**2651 TGACTCTATG GAGACTTGCA CACACATGCA CATATTCACA TAACTAAAAT**

**2701 AAACTAAAGA GAATGATGTA GGTCTGTGAA ACATTGTGCA GTATCTTACT**

**2751 CTGGCAAGAC CCAGTTCTAT AATAATGAGC AAATGCGCAA AACCTCACGC**

**2801 CGAGAAACCA CGTAGAGTTA TCTCTGTTTA ACTGTTGCTT GGTTTTGCTT**

**2851 TGGTGTTTGT TCGTTATCCA GGCTCAGGAG GCCGAAGGGG GAGGATTAGG**

**2901 AGTTGGAAGC CATCTTAGGT AAAACAGTAA GACTTGGACA GGCTCTTTTT**

**2951 AAAGCTTTTA TCTTTTTTTT TTTTTTTTTT TTTTTTTTTT TTTAGTTTCA**

**3001 TGTGCATTGG CATTTTGCCT GCGTGTGTGT CTGTGTGAGC GTGTCACATC**

**3051 CCCTAGAACT GGGGTTACAG GCAGCTCTGA TCTGCCATGT GGGTGCTGGG**

**3101 AATTGAAGCA GTCAGTGCTC GTAACCACTG AGCCATCTCT CCAGACCCTC**

**3151 TTTTTAAAAT TTTACACCTA GTTTGTTTTG TGTGTGTGTG TGTGTGTGTG**

**3201 TGTGTGTGTG TGTGTGTGTT TAGGATACAC ACGCCACAGT ATATGTATGG**

**3251 TGATCTGAGG ACAGCTATCG TCTCCTCCCA CCATGTGGGG CCTGGGAATC**

**3301 AAACGCAGAT TGTCAGGCTT AGCAGGCAGC CCCTTCCCCT GGTGACCCAT**

**3351 CATGCCAACT TATGTCATTT TTTAATATCA GTGAAGAATC ATTCTAGAGT**

**3401 CAGGCATGAT GGTGATGCAA TCCTGTAATC CTAGCACCTA AAACAATAAA**

**3451 AATAGATGGG TCAGGAATTC AAGTTCACCC TCACGTACAT ACAAGTTAGA**

**3501 GCCAGCCTGG GCTATGTCGG ACCCTGTCTT AAAACAATAA CAACAGAAGA**

**3551 ATCTTTAAAA ATAATTTAGA AAGGAGATTT GGAAGCAGAG TGTGGAAGGG**

**3601 AAGGTCTGGG GGCCTTTGAG CAGGGAGTTG ACTGTACTGG TCAGACTGTG**

**3651 TGGCATCTGC TGCAAGGCCA GCCCTGGGCT GGCCCCAGTC GGCATGTGTT**

**3701 GAGTGGTAGA TGGTTCTGGA AGTCCTGTTA TCTAGGCTGG GCAGTAAGCA**

**3751 GTAAACGTAA TGTCCCTGCC CTTGTGTGGC TGATATTGTG GCACTCAGTA**

**3801 GATGTTTGTG ATTTGGGGAC GGGGGAAGGA TTAAAGAAGA GAGAGGTGGA**

**3851 CTGTCCTCAA CAGGCCATCA TATGATCTAA GACAGAAACT GAGTCAAGGA**

**3901 GGCCAGGGCA GGACCCCAGG AGAGAACACG TTGAACCAGG TGGAGCTCTA**

**3951 GGGTTGGGAG AGGGAGTCAG TGGAAGGATG TCAGGAGGCT GGCATCCCAG**

**4001 TGGGTACACT GGGAATTGGC ATGAGGGTGG TGAGGTAGGG AAGCCAATGA**

**4051 AGGGACAGGG AGGCCACTTA GTATGGGGAC TGGGAGCAGG AGCTGGTTTG**

**4101 GGGAGATTCT GAGGCCACTT TGGAACATGG TTGGAGCAAG GTGGGGTCGC**

**4151 CCTGAGAGGC TCATGTCCGG TGTCAGAGAA GTGGCATCAC CAATGACTAG**

**4201 GCTGTGGTAT AGCCAGCCGA GGCACCTAGG GGCTCGCTGC TCGGCTTCAC**

**4251 AGCAACCTCT GAGCTATATG GACAAGTTGC GGCCCATGGG CTTGGAGGTC**

**4301 TATCTCGAGG TCACCTAGCA TGCATGAAGC CATGGCTTCA GGCCCTAGCA**

**4351 TCTTGTCAGG CTGTGATGGC CAACTGGGTA GAGTGTTGGA TGTGCTCCCA**

**4401 ACATCATGCC CGTAATCCCA GCTTTAGAGG GGTGGAGACA GGAAGATCAG**

**4451 AAGTTCAAGT TACAAAGACT TCATAGCAAG TTTGAAGCCA GCCTGGGCTA**

**4501 CATAAAACCC TAACTCAAAA AGCAAGCTAG GCATGGTGGC ATATGCCTTT**

**4551 AATCCTAGCA GTCAGAGAGA GGCAAGTGGT AATCTAGTCT ACATAGTGAG**

**4601 TTCTAGGCCA GCTAGGGATA TATAAAGTGA GACTCTGTCA GAAGAAAAAA**

**4651 GATGGGACAA GCATGCAGGT GCATGCCTTT AAACCCAGGC CACAGATCCA**

**4701 GGCAGATCTT CAGGAATTCC AGGTCAGCCT GGTCTGTGTA GTAAGTTTTA**

**4751 GGCCAGCCAG AGAGACCCCA TCTTGAAAGA AAGATAAGGC CTAGAGGAGA**

**4801 GCCAGGTCCT CTCTCTAAAG CAACCTTTCC CAGAGTCCTT ACACTGGCCC**

**4851 TGTACTTCTT CAGGGCACCT CCCAGTCAGA GCCCTACTGT ATTTGAACCA**

**4901 GGTTGTCACC CTTCCCATCC CTTCCCTTCC CTTCCCTTCC CTTCCCTTCC**

**4951 CTTCCCTTCC CTTCCCTTCC CTTCCCTTCC CCGCTAACCC CACTTCTTGG**

**5001 GCTTGGCCAT CCCACCCACC CCTTCCAGGA TTCAGACTCG GACACTGAGG**

**5051 GAGGAGCCAC TGGTGGAGAG GCAGAGAGTA AGTGTCGTCC TGGAGGAGGG**

**5101 CAGGGAGGTG ATCTGGGGTC TGGGATTAGA GGATGGGATT CTGGGGAGAC**

**5151 CCCCTCCAAG CTACCGGAGT GATAGGCAAA GCATGTAACA ATGGTTTGGG**

**5201 ACTGGATGCT GCTGTCTGTG GGAGACATTG GGAGTTGAGA TGTGGGAGGA**

**5251 GAGTGTGTGC TTGGGCCCTG GCTCGATGCC CACCTGGCGC TGTCTCTCTT**

**5301 GTTGTTGGCC AGTGGACTTC CTGAGGAATT TATTCACCCA GACCCTGGGC**

**5351 CTGGGTTCCC AAAAGGAGCG TCTTCTAGAC GAGCTGACCC TCGAAGGAGT**

**5401 GACACGCTAC ATGCAGAGCG AGCGCTGTGA GTCCCCAATT GCTTGGCCGC**

**5451 TTCTCCCATT CCTGGCCCCA GTTGACTCCA TATTTTGGGG GGTAGGAGTA**

**5501 GTAAGAAAGC CTGCTGTATA CACCTGTAAT CCCAAACTCT GAGCAGATTG**

**5551 GGAAAGGACC AGCATTTCAA GTTAGCCTCT GCTAGACTGT GTGTGTGAGG**

**5601 CTACCCTGAG CTACATGGGA AGTTTTCCTA AAATTCAGAA AGAAAAAAGA**

**5651 TGGAGGAAAC CCAACTCTTG GGATGGAGAG ATGGTTTAGT AGTTAAGAGC**

**5701 ACTAGCTGTT TTCCCAAAAG ACCTAGGTTG GGTTCCCAGG ACTCACATGA**

**5751 TGGCTTACAA CCATCTGTAA CTCCAGTTTC AGGGGATCCT ACTTATGGCC**

**5801 ACCATGGGCA TCAGGCATGC ATGTAGCACA CAACATTCAC GAAGGAAAAC**

**5851 ACCCATGTAG TTAAGAAAAA AAACAAAAAA CTCAGCTTGC TGGGGGGGGG**

**5901 GGTGGTGAAC GCCTTTTACC CCAGCACTCG GGAGGCAGAG GCAGGCAGAT**

**5951 TTCTATAGTT TGAGGCCAGC CTGGTCTGCA GAGTGAGTTC CAGGAGGTGA**

**6001 CTGAAGCCAG AAGATCATGA GTTTGAGGGT AACATGTGCT ACATAAGATT**

**6051 GTTAACTCAA AGAAAGAAAA GAGAAAGACA AGAAACCAGA ACTCTAGCCA**

**6101 AAAACAATAA AAAGGAGCAT GCAGACTCTT AAGCATTAAA TGTGATGGAG**

**6151 CCAGGCTCGA GGACATTGTT AGGACATTGT TCTCAGGCCT CACAAGTCTG**

**6201 TCTGCGTAGG GTGAAGGAAA GTCCAGCAGG CGGCTCCTGC TTGGGAGGGT**

**6251 ACCTTTGCAC TTAAGAACAA GGCCACATTG GCCCAGAAGC CCCCCCCCCC**

**6301 CCCCCCCGGA TGGAACTCAG CCCCTACACT CACACACCAC GCTAGGCAGG**

**6351 CACTCTGTCA TTGAGCTACA GCCCCAGCCC TTCCTTTCCT ACTGTTTTAT**

**6401 TTTGAACGCC CTGAATTTAC CCTCTAGTCC CTGAAGGTCG ATCCTCTTAC**

**6451 CTCAGCCTCC CACATAGCTG GGAGTACAGA GTAGCACCAC TAGGCCTGCC**

**6501 TTTGGGTCAC TTCCCTAGTG AATCTATTAC TGTCTTCTGA TGGGCCAGGC**

**6551 CTATGCCCTA GACTGGAAGT GATCAAAGCT CTGGGGTGAC TCAGGTGAGG**

**6601 CTAAGGAAAT TGCCACATGC TGAGCATACA TATGCTCAGC ATGCCGAGCT**

**6651 CATCTGCAGG CCCAGCCACT CTGGAAGCTG AGGCTAGAGC ATGTGAATTC**

**6701 AGAGGCAGCC TGGACTACAG AAAAAAGAGG GAAATGGAAT GATGATGGTG**

**6751 GCCCACACCT TTAATCCCAG CAGAGGCAGG GGGATTTCTG AGTTTGAGAT**

**6801 CTGCCTGGTC TACAGAGTGA GTTCCGGGAA AGCCAGGGTT ACACAGAGAA**

**6851 ACCCTGTCTC AAAACACGAG AGAGAGACAG AGAGAGACAG AGAGAGAGAG**

**6901 ACAAAGAGAG ACAGAGAGAG AGAGACAGAG AGAGAGACAG AGAGAGACAG**

**6951 AGAGAGACAG AGAGACTTAG GAGATTATCT CAGTCCATTA AGTGCTGGCA**

**7001 GCTCCCACAC AAGCAGCTCA CAAGTGCTTG TAATTCCCGG GCTGGGGAGG**

**7051 CAGAGGCAGG AGCCAAGCTC AGGTCCCCAT GAGACTCGTG AGACCCTGCC**

**7101 TTAAAATCCG TGATGGCACC AGTTGTCCTC TCACTTCCTT CCACATGTTT**

**7151 GCTGTGAAAT ACACGCACAA AGAGATGTGT GCACACCACC AAACAAACAA**

**7201 ATAACAGCCC ATATACACAC TAGGTGGATG GCCCTGGAGA AATACACACA**

**7251 CACACACACA CACACACACA CACACACACA CACTCGAGAG ATAAGTGATT**

**7301 AGAACAGGAC CCGATCTCTT GAGAGATACA GAAGTGTTTG CCATGGTATT**

**7351 ATTACAGGAA GCTTGTCTCA GACGTGCTGT TTAGAAGCCA GCTAGCTGGG**

**7401 TCACCGTGAA ATTGATATTT CTTGCTGAGG TCGAGTCACC GAAGTCTGAG**

**7451 AACCCAGGCC CTCGCAGTCC CCGTTGGGAA CAATGCAGCT TTGAGGGTCC**

**7501 TGAACAGCCC TTTGTTGAGG CCATCTTCTC CAGTTTCCCC TTGGCCCAGC**

**7551 TGCTCAGCGC CCTCCTTGGC AGCCGGGATG GCAGCTGAAC CTCCCTCTCC**

**7601 TGCCACAGGC TGGGCAGCCG GCCGCCTACA GAGCATACCC ACACAGTGGC**

**7651 CAGGCGACAC TGGTCCTCCC TCTTACACTC AGATCTGAGA GTCCCTGGCC**

**7701 CATAGGGTGC TGGTGTGGGA CGTGACATAA ATATCAACTA AGCAGACCCT**

**7751 TGCTGTTCGC CAGGCTCTGG GCTAAGTAGC TCACCAAGCA TCCTTGAACC**

**7801 TAATTATCAC GCAATAGCTG TGAGGTCTTT CTAGAAGTGA GCTGAACAGA**

**7851 AGAGGAAACT GAGGCACGGA GAGGTTGTGA CTTGGTGTGA ACATATGCAA**

**7901 GGGGCACAGT CAGGGCCCTT GAAGCTCGGT CTTGTTCTGT AGCTGTGGCA**

**7951 AGCTTTGAAT TTAAGATTTT CTTGCCTCAG TCTCTGGAGT TTTGGTATTA**

**8001 CAGTGTTACC ACACCCAGTC TCAATGCCTA CGATCTCCAG CCAGACTTGG**

**8051 GCCAGTAACA TCCTAAAGAA GCTGAGTGCA TAGACTGTAG CAGGCTCTCT**

**8101 GCTGTCATTT GTTTTTGGTT TGGTTTGGTT TTTTTTTTTT TTTTTTCTAT**

**8151 GTAACAGACC TTGCTGGTCT GCAATTCACT TTATAGACCA CACTGGCCTC**

**8201 AAACTCACAG AGATCCTCCT GCCTCTGCTT CCCAAGTTCT GGGAATAAAC**

**8251 ACATACGCCA CCATGCCTGG TTTAAACATT ATCTTTGAGT GTGTGTGCTG**

**8301 GGGGTGGGTG GGTGATGTGG GCATTACTGT AGGTGCATGC ATGCTACTGT**

**8351 GTTTGCCAGG GGACATCTTT GAGGAGTTAT TTTTCTCGTT CTACCGTGTA**

**8401 GTTTCCGGGA ATCAGGCTTA GCAGCAAGTG CCTTTACCCA CTGAGCCATC**

**8451 TCGCCAGCCC CGATGGCAGA CTGACTTCTG TTCACACGTG TCCCTTAAAC**

**8501 AGAACACCCC AGGGGTGCCC AATTCATGGG ATACCACACT TGTGTCTGCC**

**8551 CATCAGCTGA CAGACAGATG TCGGGGCTCT GTGTGGCTGG GCTGTAGGCC**

**8601 AGCCAGCAGC TGGGGCAGCA ATAGGGCCAT TTCTTCTCAG CTCTGTGCCT**

**8651 GTGAATGGAT TCAATCCTAT CACCCCCTAA TAGTTCTTTA AAAACCAAGA**

**8701 ACAAAGCCCC CATAAGAGTA TGAACCATGG ATCTGAAGAA GCATAATACA**

**8751 CCGGAGGGGC TGGGGGCTCG GCTTGGCTCG GTTGGTGGAG TGCTTGCTTA**

**8801 GTGCACAGGC CCCTAGAGTC AGTCTCCTGC ACCACACACA ACAACGGGCA**

**8851 TAGCAAGGCT TGGTGGCACC CACTCCTCCC AACGCTTGCA AGGTCACTGC**

**8901 GAGTTCTCAT GCTGAGACCC TGTCTCAAAG AAAGAAAAGA AAAGCTGGGC**

**8951 ATGATGGCAA TGCTGGGCAC TTAAAGATGG AAGCAGGGTG ATTGGAAGTT**

**9001 CAAGGTGATC CTTGGCTGGC TGCACAGTGA ATTTAATGCT TGCCTGACGT**

**9051 CCATGAGATC CTGTCCAAAA CAAACAAACA AAAAAACCGT TATTTAGTGC**

**9101 AATTGACTCA AAGGAGCAGA CTTTAGGCTT TTGTGTGAAC AAAGCAGGAT**

**9151 GGTGAAGGGA TGTGTACAGC TAGAAACAGG TCCTAGCCAG TCATTGGAGC**

**9201 TCCTGGGAGG CTGAGGAGGG AGGTACAGGA TTCCAGGCCA GCCCAGGGTA**

**9251 CATAGGGAGG CACTGACTCA AAACCAGAGA AACCAGCAGA CATGCTGGTG**

**9301 CACACCACAA CACCAGCACC CAAGAGGTGG AGCCAGGAGG CTCCGAAGTT**

**9351 CAAGGTCAGC CTTAGCTACA TAGAAGGCTC AAGGTCAGCC TTGGCTATAT**

**9401 GAAACCTTGT CTCAAATTTT CTTTTTTAAG TGTGTGTGTG TGTGTGTGTG**

**9451 TGTGTGTGTA TAAGCATGAG TGTAGATGTA CAGATGCCCT CAAAGGTCAG**

**9501 AGGCACAGGA TCCATGGAGC TGGAGTTAAC AGGTGGCTGT GAGCTCTGCC**

**9551 TGACAGGGGT GCTGGAAATT GAACTCAGGA CCTCTGGAAA AGGAGGAGGT**

**9601 GTTCTACCCA CTGAGCCATC TATGTAGCCC TCATCTGTGT GTGTGTGTGT**

**9651 GTGTGTTTTG AACAAGGTTT CTCTGTGTGT CTTTGGCTGT CTTGAAACTC**

**9701 ACTCTGTAGA CCAGGCTGGC CTCAAACCCA CAGAAATCTG TCTGCCTCTG**

**9751 CCTCCCGAGT GCTTGGATTA AAGGTGTACA CAACTACCTT CTGACCCAAA**

**9801 AGTAATTTTT TTGAAGTCTT ACAGTATTGT ACCTAGAAGA TTAGTCATTG**

**9851 TCAGAATTTG GTTTGGATTG ATTGTCAAGG ACTCAAACTA GCCGTCTCTG**

**9901 TACTGCCGAA CTTTGCAAGG CGTCCCCACC TCACCCCACC CCCCATCCCT**

**9951 CTCCCTTCTC CTGGGTGGCT GTGTCTCCTC ACCTGAAATG TGAGGTTGCC**

**10001 AATGCCATCC TGCCCGGCAC CAGCAGTACG TCTAGGAGGG AAACCTTAGC**

**10051 ATGTGCCATC AATCCTCTGT TCCAGGCCGC AAGGTCATCT GTTTGGTGGG**

**10101 AGCCGGAATC TCCACGTGTA AGTTCCCCGT CCTTCAGCCA CTCCTGGGGC**

**10151 TGGGGGAGGC TGGAGGGGTT CGTGCACACG TGGGCAGAGC CTCTGACTGC**

**10201 GTTGCCTTCC TAGCCGCGGG TATCCCTGAC TTCCGCTCCC CGTCCACTGG**

**10251 CCTCTATGCA AACCTGGAGA AGTACCACCT TCCTTACCCA GAGGCCATCT**

**10301 TTGAGATCAG CTACTTCAAG GTAGACGGCT AAGATGAGGG GAGGTGGCTC**

**10351 CCAACTCCAG TCCTTGTCTT ACCTGGGACA TGTGGCCCTC TGCTCCTCTC**

**10401 TCCCACAGAA ACATCCGGAA CCCTTCTTTG CCCTTGCCAA GGAGCTCTAT**

**10451 CCCGGGCAGT TCAAGGTGAG GTCATCTTCT GCAGGGAGCC TGGGAAGAGT**

**10501 AGGCATGGAA GCAGGCTCTT TATAGGCCCT CCAGGCAAGC ATAACATGAT**

**10551 TAAGAACCTA GCTTCTGGGG CCAGCAGGAT GGTTCCATGG GTAAAGGTGC**

**10601 TTGCTACCAA ACCTAATGAC CTGAGTTCAA TCCCTGGAAT CCTCATGACT**

**10651 GAAGGAGAGA ACTGACTCCA TCATATTCTC TGACCTCCAC ATGTGCACTG**

**10701 TGGCAAGTGC ATATCCCCAG GTAGATGGTT GGATGGAGGG AGAGACAGAT**

**10751 GGGTGGGTAG GTAGATAGAT GGATGGATGG ATAGATGGAT GGATGGTGGG**

**10801 TAGATAGATG GATGGATGAT AGATAGATAG ATAGATAGAT AGATAGATAG**

**10851 ATAGATAGAT AGACAGATAG GTAGATGGGT AGATGGGTAG ATAGAGATAT**

**10901 TGTTAAAGAA AAAAGAAAAG AATTACATAA GTTCTGGCCA GATTTGATAG**

**10951 TTCATCCCCA TAACTGTAGC ATTTGGGAGG CAGAGACAGG AAACTTTCAA**

**11001 ATGCAAGGTC AGCCAAAACT ACCTATCTCA AAAGTAAAAA AAAAAATAAA**

**11051 AATTAAAGAT GAAAGAGGTG AAAGTGGGAG AGATGGCTCA GGGTAAAAGA**

**11101 GCAGATAGTA CTCTTCCAGA GGACCTGGGT TCAATTCCCA GGACCCACAT**

**11151 CAGGATGTTC ATAGCCCCCT GTAACTACAG CTCCGGGGAG CCTGACACCT**

**11201 CTGGCCTCTG TGGGCACTAG AACTCACCTC CATATACCAA CAGAGACCCA**

**11251 CACACATATC TGTAACTAAA AATAATTGGA ATGGTAGCTT AGCAGTTCAG**

**11301 TGCACTGGCT CCTCTTCCAG AGGACCTGGG TTCAATTCCC AGACCCACAT**

**11351 GGCAGCCTAC AGCTATCTGT AACTCCAGTT CCAGGGGATC TGACACACAC**

**11401 ACACACACAT GCAGACAAAA CACCAGTCAA TGCACATAAA ATAAAAATAA**

**11451 ATAAATCATT TAAAAAATTC CAGCTCGGGG GCCGGGCGGT GGTGGTGCAG**

**11501 GCCTTTAATC TCAGCACTTG GAAGGCAGAG GCAGGCAGGT TTCCGAGATC**

**11551 GAGGCCAGCC TGGTCTACAG AGTGAGTTCC AGGACAGCCA GGGCTATACA**

**11601 GAGAAACCCT GACTCGAAAA CAAACAAGCA AACAAACAAA CAAACAAACA**

**11651 AAAACAAGAA ACAAAACAAA AAAGAAAACA AACAAAAAAA TCCAGCTCTT**

**11701 GGTGAGAGGC AGGAAGATTT CTGTGAGTTT GAGGCTAGCC AATTCCAGGC**

**11751 TTACCTAGAG AGACCCTATC TCAAAAACCA AAAAAATAAA TAAATAGTAA**

**11801 AATAAACAAT TTTAAGAAAT TAAAAGAAAG GAGGAGTGGG TTGCCTGGCC**

**11851 CCTCGTCTCT TTATCCTGTG GCTCTGGGCC TCAGTTTACC CAACTCTAAG**

**11901 AAAGCTGCTG AGACTATCAG GAAGGGCTGA GACTGAAGCC TGGCTGTGAG**

**11951 CCTGACTCTG CAGTTTACCT ATTGGTGATG CCAAAAAGCC GCCACCATGC**

**12001 CCTTGCCTGG GTCTCCTCAT TTGTGGTACG GGAGAAGGCA GGCTCACCCT**

**12051 CACTGGGCCA CTGAGATGGG GCCATGGGGC AGAATGGGCC CACAGCATGG**

**12101 CCTGGGACAT TCACATCAGC ACTGTTACTC TTCTCTCAGC CAACCATCTG**

**12151 CCACTACTTC ATCCGCCTGC TGAAGGAGAA GGGGCTGCTG CTGCGCTGCT**

**12201 ACACGCAGGT AGGCGGGGCC ACGGGCAGGT GTGACCATGG GTAGGCAGGA**

**12251 CCTTGGGCAA GCGTGACCTT GGGTGGCAGG GCTCGAGGGC AGTAGGCCAG**

**12301 ATGGGTCTTG GAACACTCGG TCAGAGCTGG GCTAACACAG GCAGGCACAG**

**12351 GACCAGGGCT CAGCTAATTA TAGTGAATGA CCTACAATGG GCCACTTCCT**

**12401 CTTGGCCTCA GTTTCCCCTT TGTAAAATAG GGGACGGAAA CAGGATAGAT**

**12451 TGGTACTCAT TGGAATCAGG AGAAAGGTCA GAAATTCAAG GTTGCTGGAT**

**12501 GGTAGTGGCA TATGCCTTTA ATTCCAGTAC TCAAAAGGCA GATGCATGCA**

**12551 GATCTCTGTG AGTTTGAGGT CAGCCTGGTC TACATAGAAA GTTCTAGAAG**

**12601 AGCCAGGGCT ACACAGAGAA ACCCTGTCTT GAAAGACCAA AAAAAAAAAA**

**12651 ACCCAAACAA CAACAAAAAC CAAAGAAAGG TTATCATTGG CTACAAAATG**

**12701 AGTTTAAGGC CAACCTTCAG GATGTGAGAC TGTATCTCTA AATTAATTGA**

**12751 TTAGTCGATT AATTACAATA TAAACTAATA GAAGAATGTT TAAATATAAA**

**12801 GAAGTCAGAA CAGCTTCAAG CCCTCTCAAG TCTGCTGTGA GGTGACAGGT**

**12851 GACAGGACAT CCCAGACTTA CTGCAAAAGG CCTCTGTATC TGAGGGGACA**

**12901 CCGTCCATAC CACAGCCACT CCAAATCAGG AAAGTGACAC TGTATCGTAA**

**12951 GGAAGGTGGG CTTCTTATTC ATACACTTCT GAGCCTGGCC TTGGATACGG**

**13001 TGGCTAATGG GACACTGTGG CCACACTGAG GTTCAGACGG ATGTTCAGGC**

**13051 TGCTGGCTTC TATGTGTCTC TGTGCGTTTT GTGGATGTGT TGAAGTTCAG**

**13101 ACTGCTCTGT GTATACATGT GTGCTAAAAA ATTGTCTCAG AATACAAAGT**

**13151 GGCCATTACA TTACACACTT GTTATCCCAG TGCTTGGGAA GCCGAGGAAG**

**13201 GAGCTGGTCA TGAGTTCAAG GCCAGCCTGG GCTAATAGTG GATTTAAGGT**

**13251 CAGACTGGCT CCATAGCAAA CAATCTCCCC CGCCCCCCGC CCCCTCCCCC**

**13301 GCCCTGCCCA AGAAGAAAAA GTTAGAAAGG AAAAAGAGAG AACTCCAAAT**

**13351 TGTACAGAAG AGTTAATGCT TGAATACAAA TAGGTTTGAA GCTCTTGCAA**

**13401 TGTCAGAAAT GGCCACCAGG GGGAGGATGG GTTCAAACTT TTGGGTCCAG**

**13451 ATGGCTGGAG GCCCCAGCTG GGGAAGAGAC AGCTTAGTCC TTCCAGGCAC**

**13501 CGCCCAAGCT CAGAGTCAGC CTCATTCACA GAAAGAGGCT CAGGCCTGAT**

**13551 ACTAACAGCT TCCTTGAAGC CCCATACTTC CTGCTTGTTG TTGCATGTCC**

**13601 CTCCTGCACC CTTGTTTGCT GGCCTCTATG ACAACAGGTG TCCACCCAAG**

**13651 TACCAGGCCC TACCAGTGAG CCAAGCAGAC ACCGCTGCCC TCCTGGTGCC**

**13701 AATTCATGTG TGTGACAAGA ACAAGAGGGA CGATGAGTCA TAGACCTAGG**

**13751 GTGTCAGATG GGGCTGAGAC CAGCCTGAAG TTTGGAAGTC AGAGGACAAG**

**13801 GTGCAGGAGT CAGTCCTTCC TTTCCATCGT GTGGGTCTCA GGGATGGAGC**

**13851 TCAGGTCATC AGGCTTGGTG GCCTTTACCT GAGCATCTCA CTGGCTCCAC**

**13901 ACATAAACTT TAAAGGGAGA TTTATTTTTA TTTCATGTTT ATGGATGTTC**

**13951 TTCCTGCTTG TATGTCCATG ACCTATGTGT ATGCAGTGCC CACAGTAGCC**

**14001 AAGAGAGGCT TTAACAGATG TGTGCTGCCA TGTGGGCGCT GGGTCCCAAC**

**14051 CCGGGGTCTT CTGGGAGAGC ATCCAACACT CTTAACTGCT GAGCCATCCC**

**14101 TCCAGCCCCC ACACGTACAT TTTTGTAAAA TATCTGAAGG TGGAAAAAAA**

**14151 TGTTCCACTT GCCCCCTTTG TGGTCAGCTT TTTTTCCTTC GTGGCCTCTT**

**14201 CTCCTCAGTC ACCGTTTGAA TCACTGACCC ACTTTCTGGA ACACAGTGAC**

**14251 TCCGCCTCTG AGTTGTGCCG ACACAGCATC TTTGAAGCTG TGGTGCTGTT**

**14301 TTTAGCTCTG CACAGGAACC CATCGCTCAA GCCACTGTGG TCCCCACAGA**

**14351 AGAGTGGCTA TAGACCTTCA AGTTGCATAC ATGAAATTCA CCCTCCAGGA**

**14401 AGTTCTCTGT ATGCCCCAAA CCCAGCCTCT TGTCTGGGGA CCCCTCGATA**

**14451 CTCACACCAT AGAGCAGCAC CATGCTTTTT AAAATCGAAG AGTAGTCTTT**

**14501 TTCTTTTTTT CTTTTTCCTT TTATTTTGGG TGTGTGTCTG CTGCGTTAGT**

**14551 TATATAGGCC GCAGGGCTTC CGGCATGCTG GGGAAGTGCT CTACCACTGA**

**14601 GCCACACCCC CAGCCCAGGC AAGACCTGAG TTTGTAATCT TCTGTCTCAC**

**14651 CCTCCTGTAG CTGAGGTTAC AGGCTTGCTC TGCTAGCCCA GCTCTACTGT**

**14701 GCAGGCATTG GGTACAGGCG GCAGCCTACC ACGTCATCAT GAGGGGAAAC**

**14751 TGCAAACTTA CATCAGTGAT GTGCAGTAGG GTAGGTGTCA GAGGCCTGCT**

**14801 CCACTCAGGG TAGACCCAAC TCACGGTGGG CTTATGAGGA CAGAGCCCCA**

**14851 TTGCAAGGGA AGGAGCGTGT CTGTGGATAT AATCCAACAT GATCAGAGAT**

**14901 CTCAGGCATC TCCCTCCACC CTAGACTATG TTTCCTTTGA TCACAGAGGC**

**14951 TGCCGGAGGT AATTCTGTGA TCCCTTAGTT AGCTGTTTGT GTGTGTGTGT**

**15001 GCTGCTCACT CTCCAATGAA GGGCTTGGTA GACTGAGCCA GCGACAAAGC**

**15051 AGATGCAGAG TGCGGGGTGT GGCAGTGCTC CCCTGTCATC CTGGCACTCT**

**15101 GGAGGGAGGT TCTCCAGTTT GAGGCAGTCT CAAAAAATCA TATCCCCCCC**

**15151 CAAAGAAACA AATAAGTCAT TCTCTTGTTT TATTTATTCA TATTACATTA**

**15201 TTTAGTGTGT GTAAAGTTCA GCTGACAACT GAAGGAAGGC GTTTGCTTCT**

**15251 ACCATGTGAT GTCTGGGGCC CAAAGCCACG TTGCTATGTT GGTAGCAAGG**

**15301 ACCATTGTCA GCTGAGCCAT CTTGTCAGCC CTTACTGTGG CTTTTCCAGA**

**15351 CAGGTGCTAG TCTTGTCCCA GCTTGCCTGA ATCTCACCCT GTTCCTCAGG**

**15401 CTAGCCTCAC CTTTACAGCA GTCTAGAATT ATAGGCATGG CCTACCATGC**

**15451 CTGGCTTTAT TTACTTATTG CTTATTTATT TCACTTTTTT GAGGCAGGTC**

**15501 TTCCCATGTA GCCCTGGCTG GCCTGACATT AGCTATGTAG CCTCAGCTGA**

**15551 CTTTAAACTT AAAGCAGTCC TCCTACCTCA GCCTCTAATT GCAGGGCTTA**

**15601 TAGGCAGTCA CCACCACTGC TGACCTGATT CAGTTTATTT ATGTTAATGC**

**15651 TTGGCCAGAG CTGTCACATA CAAGCTACTC AGGTGGGATC CCCACAAGGA**

**15701 TGCCACATCT GAGGGAGACG CTGAAGATAG GTACATTTAT TGCAATAAAT**

**15751 GTGTGGCAAA TGGAGGGACT GTGCGTGGCT CTCTAACAAA AGAACTTCTC**

**15801 TGAAAATTAG AGGCAGTGAC CCAAGTTTGT AGAAGAATAT CCCATGGGCA**

**15851 TCCTTTAGCA ACCTTCTGTG GCCCCACTGG GGAAGCGGGC ACTTTCCATA**

**15901 TATCCTCACC CTTCCTCATC CCCCGCAATG CCACTTCTCA GAGCCACCAG**

**15951 GGCTGGCTGT CTTCCTGACC TCCCCTCCCC CTCCAGAACA TAGACACGCT**

**16001 GGAACGAGTG GCGGGGCTGG AGCCCCAGGA CCTGGTGGAG GCCCACGGCA**

**16051 CCTTCTACAC ATCACACTGT GTCAACACCT CCTGCAGAAA AGAATACACG**

**16101 ATGGGCTGGA TGAAAGGTGA GGCTGGACTC TGCGGGCAGG CAGGCCTCGG**

**16151 GCAGGCCAGG GGGAGGTAGG GGGTGGGGGG CTCTCACTGC TTGCCTGCCA**

**16201 TAGCCAGCCT CCAAGGGCTG GGCCCCAGTG CAGTCTGCTC CATTCTTTGG**

**16251 CTCTTGCTAG TCAGTGAGTT GGTTGGTTTC CACTGAGGCT AGGCTTCCCT**

**16301 ATGTAGCCCA GGCTGGCCTG GAGCTTGCAC TTCTGCCTCA GCCTCTCTAG**

**16351 TACTGAGGTT GCTTGCAGGC GTGCACTACG GTACCCAGCT GTCTTGCTTT**

**16401 TTATGGTAGC TGTAGAAGCC TCAAGGATTC AAGGTGACAG CAGGTCTTTG**

**16451 CTCCCATGCC ACCCCTCTGC TGGGCTGTCA TCCATATGAA CCGCAGAGCA**

**16501 CGGCCAGCCA CGTGTGGACT CCGTGTAGAC TCACGTCCTC AACGGTCACA**

**16551 CAGCCTGTGT CTCTGAGGTG TTTACTATCT ATCTTACATG GCAAACACGG**

**16601 CTACCTTCTG AGGAAGGCAT TGCACAGTGG AACCTTCCGA GACGAGGAGG**

**16651 CCTGTGTCTA CGTCCAACAT GGCTCCACTT GCCCCATGTG CCTACTGAAG**

**16701 CAAAGTGTCT AACCTCTGCT GCCACCATGT GGGTCCCAGG AACTGGACTC**

**16751 AGGCGCTCAG GCTTGAGGAT GGACCACCTC GCTGGCCCAG AACAGTTTAA**

**16801 TTTTTATTAT ATTTTTTAAA GATAGGGTCT CTAATTTCAT TAAGTTCTTG**

**16851 TTGGTTTAAG TTGTAGAAGT CGCAGGAGGA ACCATGGTAC ATGCCTATTA**

**16901 GCCTATTGCT TGGGAGAGGC AAGAGGATCA GGAGTTCAGG GTTAGCCTTG**

**16951 GCTACATACT AAGTTCCAGG TCAGCCTGGG CTACATGACA ACATGAAAAT**

**17001 AAAAACTAAT AAATTTTATA TAAGTCTTAT GTAGCTCAGC CTTCCATGTT**

**17051 GGACAGTGCA GCAGGAGCCT GTGGAGAGGG AAGCTGGGTG CTGTGCTGGG**

**17101 TAAGACAAGT GCTGGAGACA CACATGGTAG CTCACGGCTG TCACTGCTGT**

**17151 CACTGTCCTC ATTCATGTGA GGACACAGCA ACTGAGAGGA CAGCTAGTCC**

**17201 CCGCGCCTGG GTGCCAGCAC CAGTCTGTTC TGGCAGCCAT TGTGCTGAGC**

**17251 CTGAAGCTGC TGTGTCAACT GTGGGAATCC ATTTCTGTAT TCTTTATGCT**

**17301 GACTTCACAA AGCCTCTGGT GTCCCCTTGA GATCAGGACA GCTGTGGCCT**

**17351 TTATAGCAGG ACCCTGGAAG GTAGCTCTTC TGACAGAATG CCAGCCAGCT**

**17401 GCCCCCAGAC TGAGGGTTCC TGAGCAGCCC ATGTGTGCAC AGCTGGGGGT**

**17451 GACGGACAAT GGACATCACC CCCTTTATGT CCCTGTGGAA TCGCATCTTC**

**17501 CACCCACAGA ATAGCATATT TGTGCAGAAA TACCTCAGCA GCAGCCGTTA**

**17551 AAGCTTCGCC CTGTGACTGG CTGTATGAGT GGCTGGCGGA GTGAGCTCCA**

**17601 GTCCTGTGGG CTGCATGTGG CAGATGTGCT TTCAGACTCC GTGATCCATA**

**17651 AGTAAGAAGG AGTGTGACTT TCATAAGGGT GGTCTGTCCC ATAAGGAGTC**

**17701 TGTCACAGAA TCCGAGAGTG GGAAACAGTT GGGCTTGAGG TTTGATCCAA**

**17751 CCCAAGGCTA TCTGTATGTC CTTTCCTGAC CCAGGGCAGA TAGGACTGTC**

**17801 CCCAGGACCC ATTGGATGAC TCCCATTCCC CAGATGGGGG TCTGGTGGAG**

**17851 TCCAGGGGCA CCTGTCTGGG GCAGATGGGT TTGCCCCTGT ATATGCCCTT**

**17901 TCACTCACTA GCTGTGGTGC TGCCAGCCAC CCCTGGATGC CTGGAGGTGA**

**17951 AGGGGACGGC TCTCTAAGAA GGGAGCTCCT CTCTGTGAAG CAGGAAAGAC**

**18001 CACTCAGGAA ATGCCTGAGT AGTGAGCTGT GCTGTTTGTT TTGTGGCGCT**

**18051 GCGGATCAGA CTAGGGGACT CGGGCATTCG AGGTCAGAGG CTCTTCCACT**

**18101 GGGCCACACC CACAGCCCCC ATACTGGGAG ACTCAAAGCA GGCATCTCCT**

**18151 GCTGAGTCGT GCCCAAGCCC TCTTGGTTCC ACCGCTGAGC TAGCCTAGCT**

**18201 CTTGGGGTTT GTGTGCGCAT GTGTGTAGGT GTGCACATGG CATAGCATGC**

**18251 GTGTGCATGT CAGGGCCATC TGCAGGAGCT GCTTCTCTCC TTTTAGCATG**

**18301 TGGGATCTGG GGACGGAACT CAGGCCACCA GGCTTGGCAG CAGGTCCCTT**

**18351 TACACACTGA GCCACCTTGA AGCCTCAGCT CTTATTTTTT TGAGATGTGG**

**18401 TTTTGTTGTG TAGCCAAGGC TGGCCTCAAA CTCACAATTC TCCTGTTTAA**

**18451 TTCTCCCCCA AGCAGGCTTG CGGCATGCAC CACTGCCACC TGCCTGGGAT**

**18501 GCATTTCTGT TGACAGGAGG GAGGGGCAGA AGAGGGCTTC TCCAGCAGCC**

**18551 CTTGGGGGAG CCTCACAACT CACCCCCCCT TCCTCTTTCC CACTCAGAGA**

**18601 AGATCTTCTC AGAAGCAACT CCCAGGTGTG AGCAGTGTCA GAGTGTGGTA**

**18651 AAGCCTGGTG AGTCCTGGGC CCGGGGCTGG AGCTGGGACC CCTTCCTCCA**

**18701 CGGCCTCACC CCCCAATCTG GCCACTCTGC CACCTGGCCC GTCCCCTACT**

**18751 GTTCAGCAGA GGGTGCCCCG CCCCCCAAGC CTCAGTTGGG CCCCTGACTC**

**18801 CTGACTGTTC TAGCTCTGTC TCCCATATCT CCTGTCCTCT GTCCCTCTGT**

**18851 CCCTCTGTCC CTCTGTCTGT GTCTACCGCA GATCTGAAGC TTCTGATGGA**

**18901 ATTAGAACTT GGCAAAACAA TACTGAGAAT GAAGTGTATG TGGAACAGAG**

**18951 GCTGCTGATC TCGTTCTTCA GGCTATGAAA CTGACACATT TGGAAACCAC**

**19001 AGTACTTAGA ACCACAAAGT GGGAATCAAG AGAAAAACAA TGATCCCACG**

**19051 AGAGATCTAT AGATCTATAG ATCATGAGTG GGAGGAATGA GCTGGCCCTT**

**19101 AATTTGGTTT TGCTTGTTTA AATTATGATA TCCAACTATG AAACATTATC**

**19151 ATAAAGCAAT AGTAAAGAGC CTTCAGTAAA GAGCAGGCAT TTATCTAATC**

**19201 CCACCCCACC CCCACCCCCG TAGCTCCAAT CCTTCCATTC AAAATGTAGG**

**19251 TACTCTGTTC TCACCCTTCT TAACAAAGTA TGACAGGAAA AACTTCCATT**

**19301 TTAGTGGACA TCTTTATTGT TTAATAGATC ATCAATTTCG ATCCGCTCCT**

**19351 GGGCACCGAA CTGCGCCGCG TGTTCAGCAG GGTCGGCGTG TTCGGTGTGT**

**19401 CCCCCGCGGT GGGCCTCGGG GGCGGGTGCG GGGTCGGCGG GGCCGCCCCG**

**19451 GGTGGCTTCG GTCGGAGCCA TGGGGTCGTG CGCTCCTTTC GGTCGGGCGC**

**19501 TGCGGGTCGT GGGGCGGGCG TCAGGCACCG GGCTTGCGGG TCATGCACCA**

**19551 GGTGCGCGGT CCTTCGGGCA CCTCGACGTC GGCGGTGACG GTGAAGCCGA**

**19601 GCCGCTCGTA GAAGGGGAGG TTGCGGGGCG CGGAGGTCTC CAGGAAGGCG**

**19651 GGCACCCCGG CGCGCTCGGC CGCCTCCACT CCGGGGAGCA CGACGGCGCT**

**19701 GCCCAGACCC TTGCCCTGGT GGTCGGGCGA GACGCCGACG GTGGCCAGGA**

**19751 ACCACGCGGG CTCCTTGGGC CGGTGCGGCG CCAGGAGGCC TTCCATCTGT**

**19801 TGCTGCGCGG CCAGCCGGGA ACCGCTCAAC TCGGCCATGC GCGGGCCGAT**

**19851 CTCGGCGAAC ACCGCCCCCG CTTCGACGCT CTCCGGCGTG GTCCAGACCG**

**19901 CCACCGCGGC GCCGTCGTCC GCGACCCACA CCTTGCCGAT GTCGAGCCCG**

**19951 ACGCGCGTGA GGAAGAGTTC TTGCAGCTCG GTGACCCGCT CGATGTGGCG**

**20001 GTCCGGGTCG ACGGTGTGGC GCGTGGCGGG GTAGTCGGCG AACGCGGCGG**

**20051 CGAGGGTGCG TACGGCCCGG GGGACGTCGT CGCGGGTGGC GAGGCGCACC**

**20101 GTGGGCTTGT ACTCGGTCAT GGTGGCGGCT GGATCGGTCG AAAGGCCCGG**

**20151 AGATGAGGAA GAGGAGAACA GCGCGGCAGA CGTGCGCTTT TGAAGCGTGC**

**20201 AGAATGCCGG GCCTCCGGAG GACCTTCGGG CGCCCGCCCC GCCCCTGAGC**

**20251 CCGCCCCTGA GCCCGCCCCC GGACCCACCC CTTCCCAGCC TCTGAGCCCA**

**20301 GAAAGCGAAG GAGCAAAGCT GCTATTGGCC GCTGCCCCAA AGGCCTACCC**

**20351 GCTTCCATTG CTCAGCGGTG CTGTCCATCT GCACGAGACT AGTGAGACGT**

**20401 GCTACTTCCA TTTGTCACGT CCTGCACGAC GCGAGCTGCG GGGCGGGGGG**

**20451 GAACTTCCTG ACTAGGGGAG GAGTAGAAGG TGGCGCGAAG GGGCCACCAA**

**20501 AGAACGGAGC CGGTTGGCGC CTACCGGTGG ATGTGGAATG TGTGAGGCCA**

**20551 GAGGCCACTT GTGTAGCGCC AAGTGCCCAG CGGGGCTGCT AAAGCGCATG**

**20601 CTCCAGACTG CCTTGGGAAA AGCGCCTCCC CTACCCGGTA GAATATCGTG**

**20651 TTTTTCGGTG AGAACCTTCC ATCGCGCTTC TTCTCCTGCA TGCAGTCAGT**

**20701 AAGTGTCCAC TCTGGGCTGG GCCCATGTGA GGGGGACAAA GCCAGAGTAG**

**20751 TGGGGTCCAA GGCCTTCCTG GTTACTCCCC GCAGGACTTC TCCAAGGTGG**

**20801 ACCTCCTCAT CATCATGGGC ACCTCCCTGC AGGTGCAGCC CTTCGCCTCC**

**20851 CTCATCAGCA AGTAGGTTGA GGAGGATGGG ACTGGGTGTC AGCTCGGGAC**

**20901 AGGGCACTAG GGCAGATCCT GAATCTCAGC TCCCCCTTCC CAGGGCACCA**

**20951 CTAGCCACCC CACGGCTGCT CATTAACAAG GAAAAGACAG GCCAGGTAAG**

**21001 TCTGCCTCAG CTTCCCTCCC CCTCTCCTCC CCCCCTCCTT CCCTGTCCCC**

**21051 CTCCCCCTCC CTCCACTTTC TTTGCCCCCT TCCTCATTTG GCCACTTCCT**

**21101 CAGGGGACAG GGCAGGTCTC TGTCCCACCT GGAAAGCACC AGATCCTTAG**

**21151 ACTTGCCAGT TCAGTCAGGA GAACATGGGA GCTGGCCTGT GCACTGGGCC**

**21201 TCTGTCATTC TAGACTCCAC TGAGCACAGA ACAGAACCGT GCCTTGAACC**

**21251 TGCTGAAGCC CAAGTGCTTC CCCGGACCCA GGAGACAGTC CAGACACTAC**

**21301 CATTCTAGTG CCCTCTCACA TATCAAAATC TGGGGACCCT CAAGTCCCTC**

**21351 CTATGAAGTG GCAGAGTGTT TATATAGAGC AATCTCTGAA GATGCTAAGC**

**21401 TGTGTCTCCG TGACGTGTGC GATACCATGT GAACTCAGGA GACAGGCTCC**

**21451 CATGCCCCCC TGCCCTCTCA GACACGCCAC GGAATTTAAG ATGACCTTGA**

**21501 ACTCCTAGTC CTCCTGCCTC TGCCTTCCAA GTTCTGGCAT GACAAGCCTG**

**21551 TGCCACCACT CCTGGCTGTG TTGTTGTTTG TTTAGCAGAA GGATAAGTTG**

**21601 CTTGCCACGC ATGCACTAGG TCCCGAGTTT AACTGCTTGC ACCCACATAA**

**21651 AAATCCAGGC ATGGTGTTTG TAGTCCCACA CTGGAGAGGC AAAGATTAGT**

**21701 GGGTCCCTGG GGCTCACCGG GTAGCCAGTC TCACATAATC CTGAATTCCA**

**21751 GGCCAGTTGA GCAATGTCTC AGAAAGAATG TGTCTGGGCC GAGGCTGTAA**

**21801 CTCAGCGGCA GTGCTTGCCT GGCGTATGCG CGGCCCTGAG TTCAATCCTC**

**21851 AGCACCACCA AAGACGAAGA CGGTTATTAG TTTTGGACTA GAGAGATGGT**

**21901 TTACTGATTA AGACCTTCTG TCCCCAGCAC CCATGTCAGG TGGCTCACAA**

**21951 CTGCCTTTAA CCCCGGCTCC TGGGCATCTG ACGCCTCTCA GTCCTCTGAG**

**22001 CACTCACACA TAGAGAGCAC ACATGCAGTC AGATAAAAAG AAGCCAAGTG**

**22051 GTGGTGGTGC ACACTTTAAT TCCAGCACTT GGGAGACAGA GGCAGGTGAA**

**22101 TCTGAGTTTG AGACCAGCCT GGTCTCCTTA GTAAATTCCA TATTAGTCAG**

**22151 GACTATATAC ATATAGACAC CATCTAATCA ATCAATCAAT AACAAGTTGG**

**22201 CTAACTTCTA AGAAACAGTA ATTATGGTTG GCCTCTTGGT TGACCTCTAG**

**22251 CCTGACCTCC AGGTCCACAT GTGTACAAAT ATAGACACAT AGCCCACTAC**

**22301 CATACACACA CACACACACA CACACACACA CACACACTAA TTAGTAAATA**

**22351 GGAAAATAAG GCTAGGTATG GTGGTGCACA TCTTTTATCC CAGCACTGAG**

**22401 GAGGCAGAGG AAAGGTGGAT CTTTGTGAGT TCAAGGCCAG CCTGGTCTAC**

**22451 AGAGCAAGCC CCAGGACAGC CAGGGCTCCA CAGAGAGACC CTGTTTTAGA**

**22501 AATAAGCATA AGGCAGGTCT GCTGGCTCAG GGCTGTGCCC TTAGCTGTCT**

**22551 TCTCCCCACA GACGGACCCC TTCCTGGGCA TGATGATGGG CCTGGGAGGT**

**22601 GGCATGGATT TTGACTCCAA GAAGGCTTAC AGGTGAGGCT GGGCCTGGGT**

**22651 GAGCAGCTCG AGAAGGGTGG GTGAGGAGGG AGAGAAGACA GAAGGCCGGC**

**22701 TGGTCCTCAG CCTGTTCTCT TACTCCTGCT CACCCTGCAG GGACGTGGCC**

**22751 TGGCTGGGTG ACTGTGATCA AGGCTGCCTG GCTCTCGCTG ACCTCCTCGG**

**22801 ATGGAAGGTG AGGAGCTGGG CCACCCCAGC CCACCCTGAG CCCAGGCCCA**

**22851 TGGGGTTTAC GGTAACCTGA CTTGCGTCAA CTTTGCCCCC TACAGAAGGA**

**22901 ACTGGAAGAC CTTGTCCGGA GGGAGCATGC CAACATAGAT GCCCAGTCAG**

**22951 GGTCACAGGC CCCCAACCCC AGCACTACCA TCTCCCCTGG AAAGTCCCCA**

**23001 CCGCCTGCCA AGGAGGCGGC CAGGACCAAA GAGAAAGAGG AACAGCAGTA**

**23051 ACAGTAACCA TGACCTCCCG CAGGACAGCG GAGCCCGGCC AGCACTGGGC**

**23101 CCTCTTAACA TGCAGCTTGT GTGAGCTCAA AGACCCTTCG TTCTTTAACC**

**23151 ACGTTCTTGA AATCAGGGTC CCCAACTCAA TCCCAGAAAA GCCTAATATA**

**23201 CCTAGGGGCT GAGGCCTGTG CAGTCTGTAG CTGGGGCCTC TAACCACCAT**

**23251 AGCCTCTAAC CACCATAGCC TCTAACCACC ATAGCCTCTA ACCACCCAGG**

**23301 CAAGAAGCAG CCTTCCCTAA CTTCTAATTA TTCCCAGACA ACAGGCTACC**

**23351 CCAAAACCCC TAACAGTGCC AGAATAAGGC ATTTCTCTAT TGTTTTCAGG**

**23401 GGGCCTATGG CTAAATCAAA TTAACCTACC CCGCATAGGG GCTGGACTCT**

**23451 ACAAATAGAA CTTCACCCAA GGGGGTGGGG CCTTGTGGGA TCTCTGAGCC**

**23501 TGAAGGCCTG CCAACTCTCT GCCTCCAACA AAGTGGGTAC TAGGCTCCCT**

**23551 TTCCTGGGGA CCCACTTGCC AGCTGTTGGT GGATGAGCAA GAGACCTTGC**

**23601 TTATTAGAAA CAAATTAAAA AACAAAACAA AGCAACTAA**
