# Supplementary figures and images for: SIRT2 Ablation Has No Effect on Tubulin Acetylation in Brain, Cholesterol Biosynthesis or the Progression of Huntington's Disease Phenotypes In Vivo
Source: PLoS One. 2012 Apr 12;7(4):e34805. doi: 10.1371/journal.pone.0034805 (PMC3325254; doi:10.1371/journal.pone.0034805)

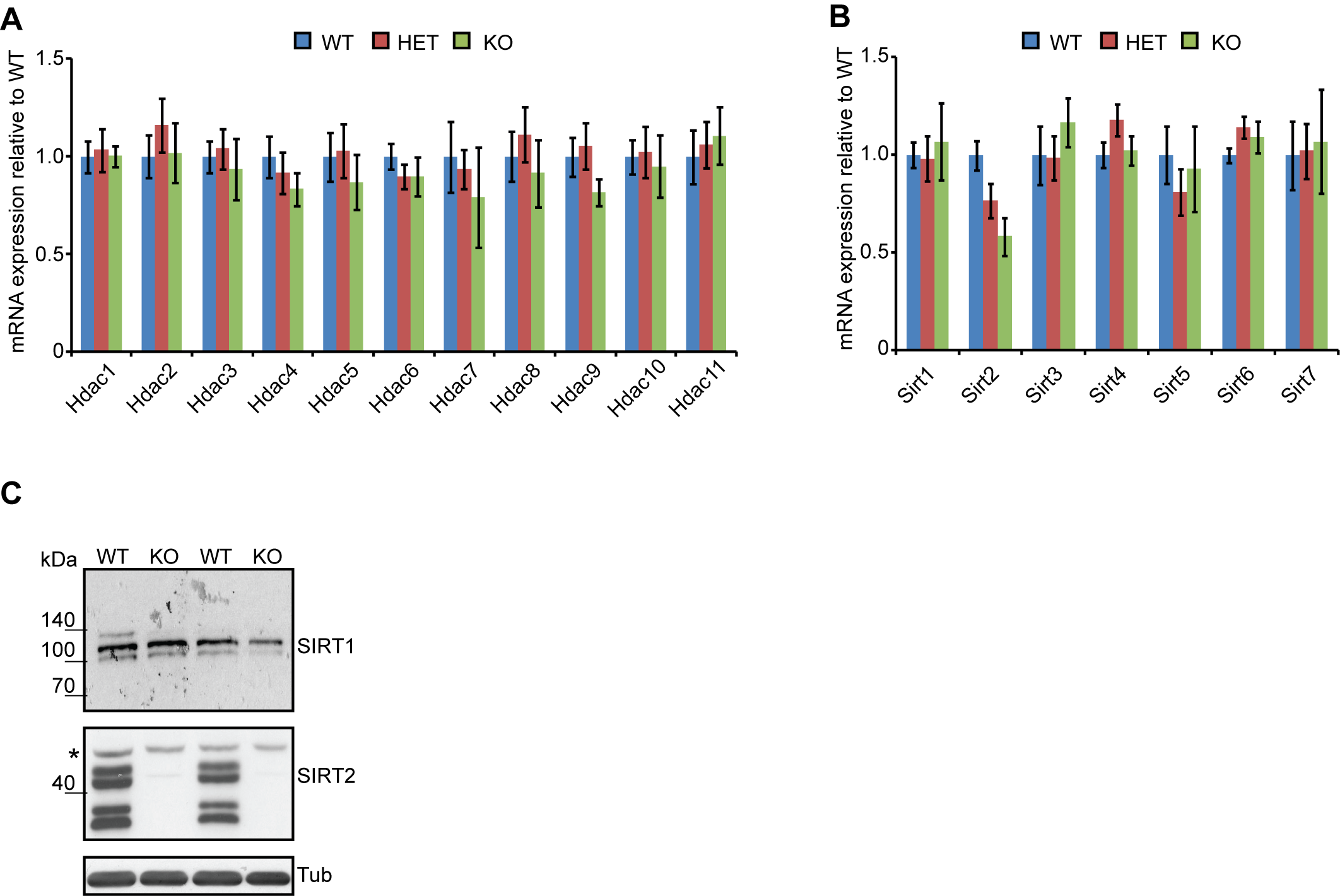

Supplement: Figure S2 — Expression of Hdacs 1-11 , Sirt1-7 and SIRT1. (A–B) Taqman qPCR assays measuring the mRNA expression of all known deacetylases in the cortex of 4 week old wild type (WT), Sirt2HET (HET) and Sirt2KO (KO) mice. Expression was normalised to the housekeeping genes Atp5b and Canx and expressed as fold change of WT ± SEM. n≥6/genotype. (C) Representative western blot probed with SIRT1 antibody showing SIRT1 expression between wild type (WT) and Sirt2KO (KO) brain lysates extracted from cortices of 9 week old mice in RIPA buffer. * denotes a non-specific band. Tubulin (Tub) was used as loading control. n≥3/genotype. (TIF) [file pone.0034805.s002.tif]

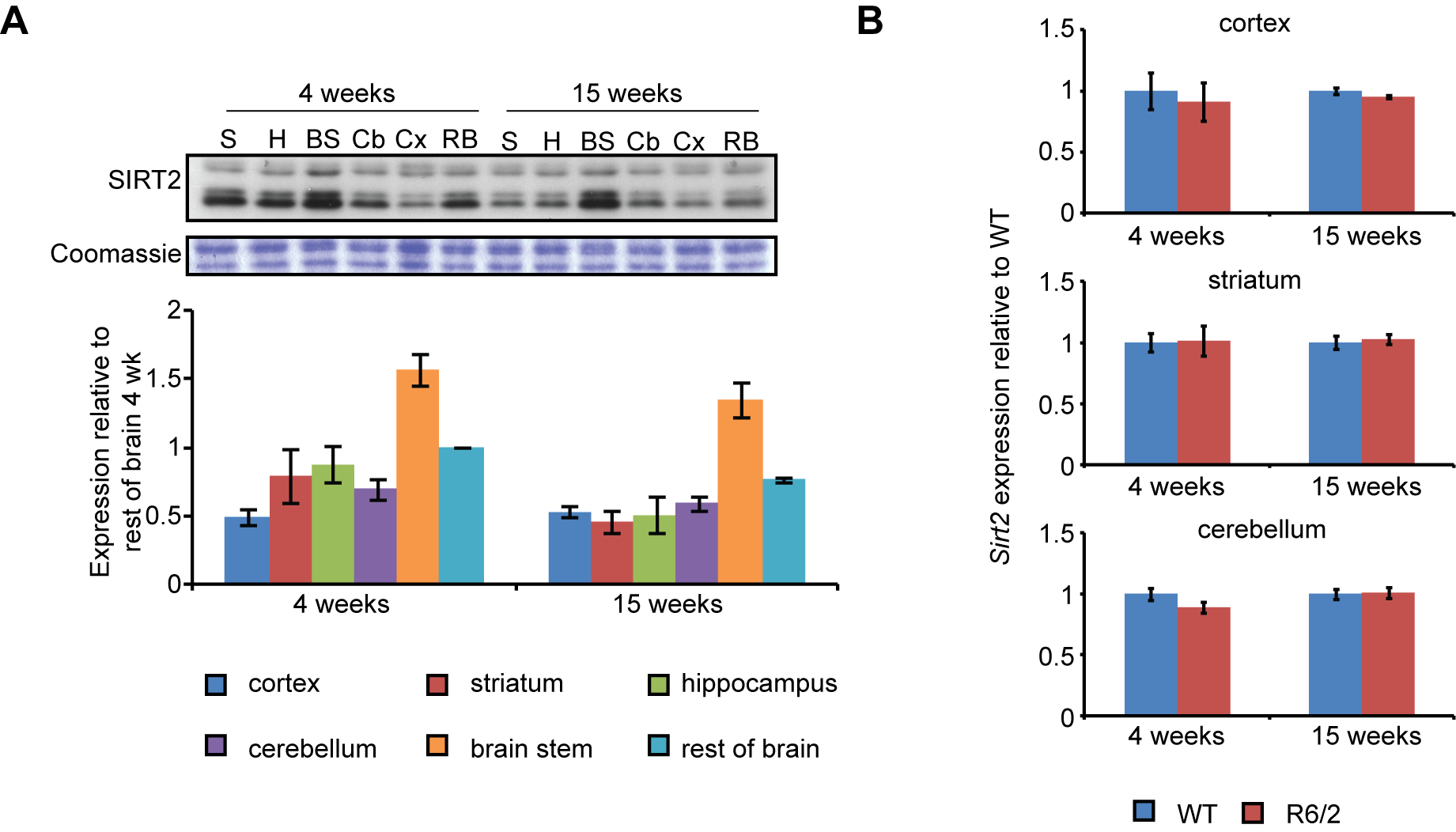

Supplement: Figure S3 — SIRT2 expression is comparable between 4 and 15 weeks and not affected by HD progression. (A) Representative western blot showing SIRT2 expression in different brain regions at 4 and 15 weeks of age in wild type mice. The signal for SIRT2 was normalised to total protein loaded (Coomasie stain) and expressed as fold change of rest of brain (Rb) (containing all regions except cortex (Cx), striatum (S), hippocampus (H), cerebellum (Cb) and brain stem (BS)) ± SEM. n = 4/brain region/time point. (B) Taqman qPCR assay measuring levels of Sirt2 mRNA between wild type (WT) and R6/2 mice at 4 and 15 weeks of age in cortex, striatum and cerebellum. Expression was normalised to the housekeeping genes Atp5b and Canx, and expressed as fold change of WT ± SEM. n≥6/genotype. (TIF) [file pone.0034805.s003.tif]

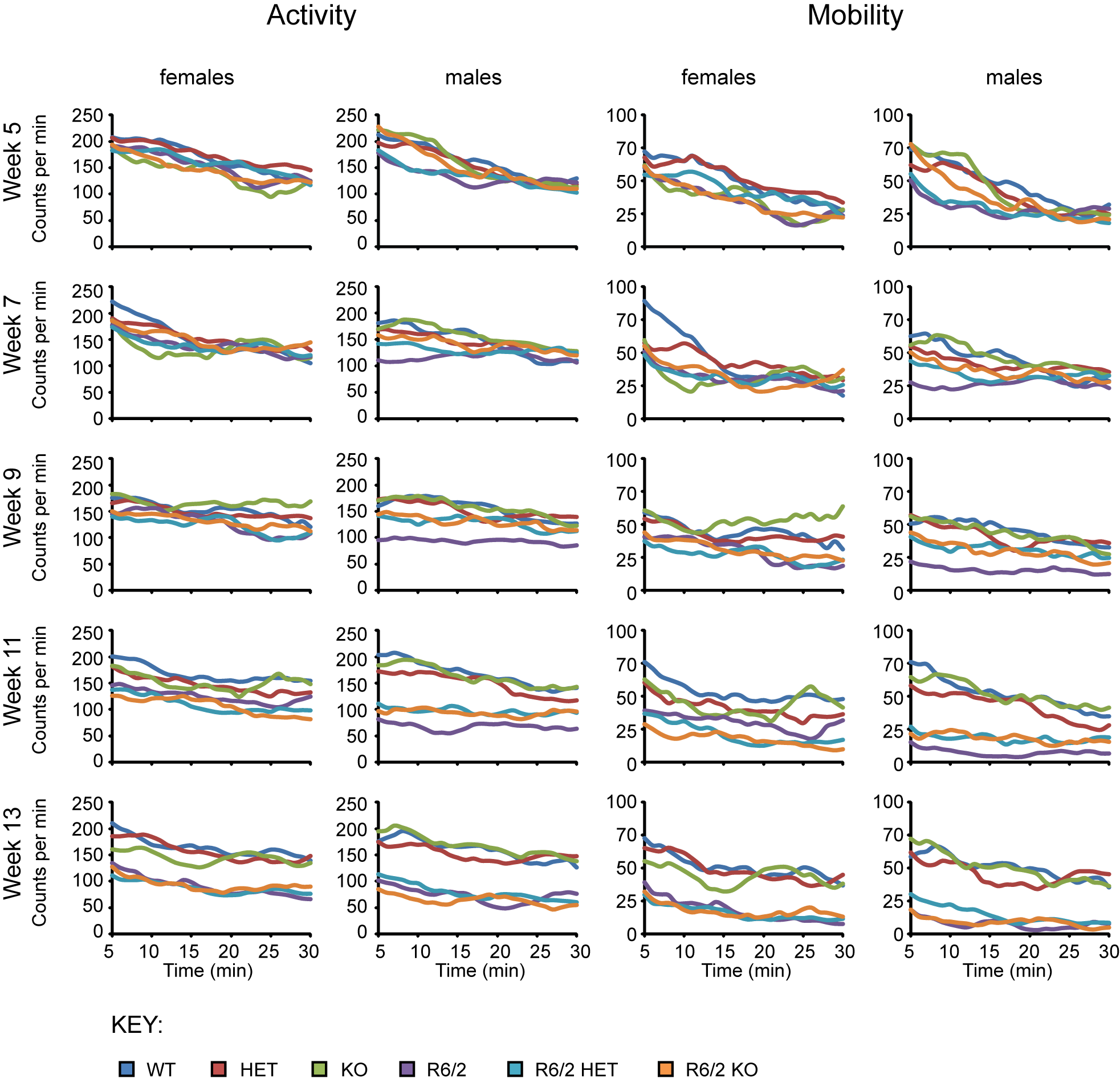

Supplement: Figure S4 — Activity and mobility. Activity (single lower beam break) and mobility (consecutive lower beam breaks) were recorded for wild type (WT), Sirt2HET (HET), Sirt2KO (KO), R6/2, Sirt2HETxR6/2 (R6/2 HET) and Sirt2KOxR6/2 (R6/2 KO) mice over the course of 30 min at 5, 7, 9, 11, and 13 weeks of age. Activity parameters measurements were visualised by plotting 5 min moving averages. (TIF) [file pone.0034805.s004.tif]

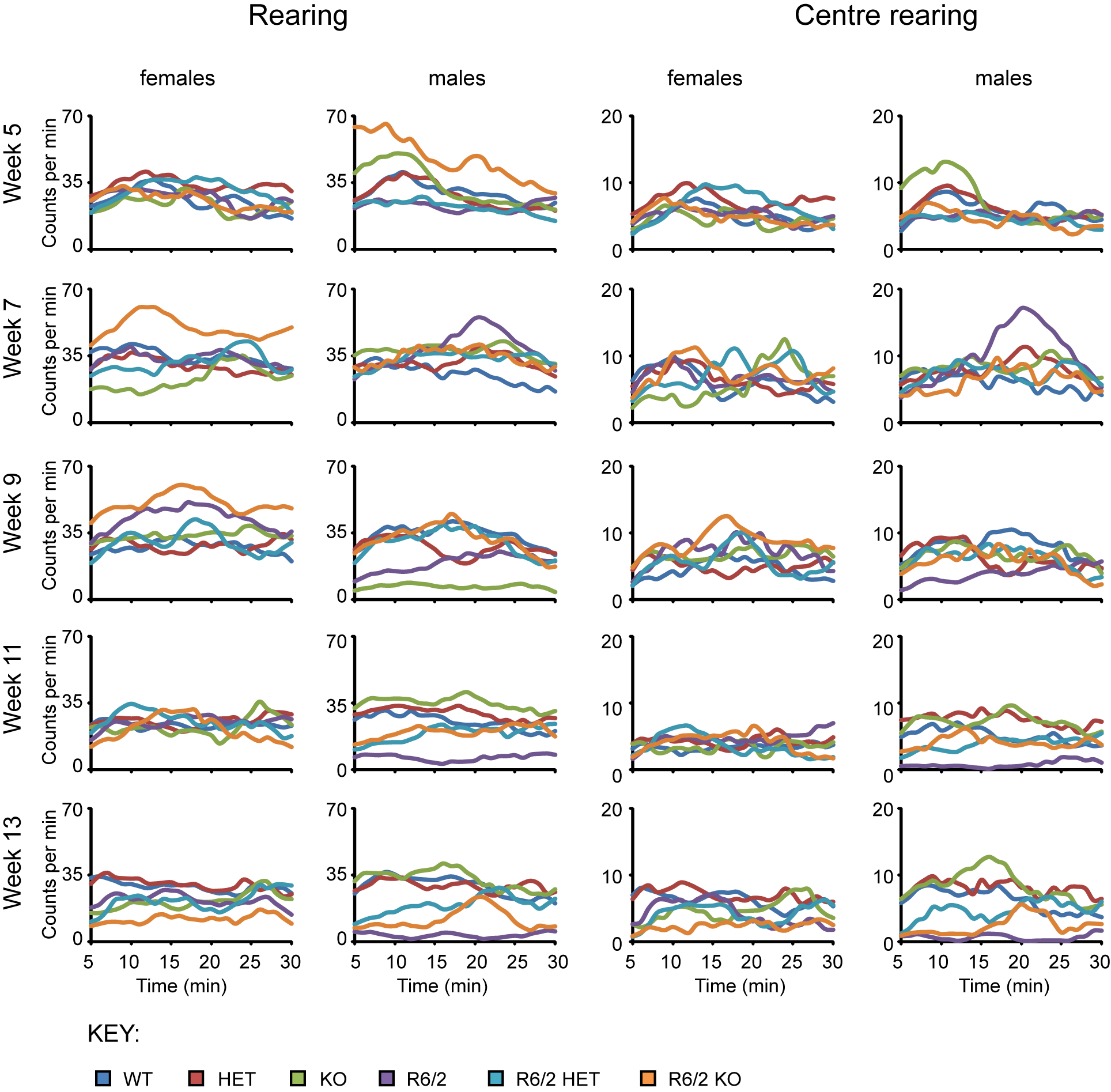

Supplement: Figure S5 — Rearing and centre rearing. Rearing (upper beam break) and centre rearing (unsupported upper beam break) were recorded for wild type (WT), Sirt2HET (HET), Sirt2KO (KO), R6/2, Sirt2HETxR6/2 (R6/2 HET) and Sirt2KOxR6/2 (R6/2 KO) mice over the course of 30 min at 5, 7, 9, 11, and 13 weeks of age. Activity parameters measurements were visualised by plotting 5 min moving averages. (TIF) [file pone.0034805.s005.tif]

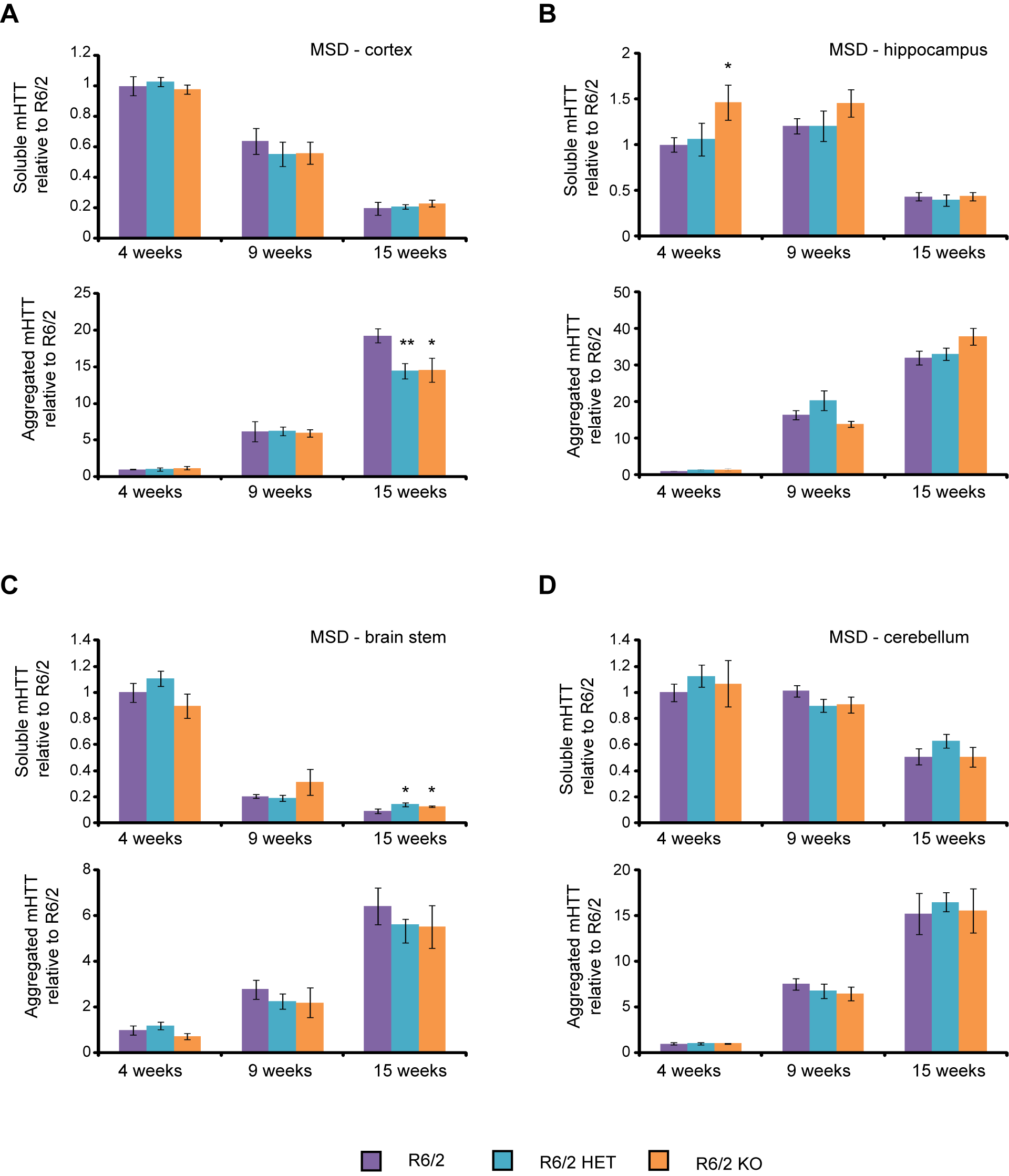

Supplement: Figure S6 — Soluble and aggregated mHTT in the cortex, hippocampus, brain stem and cerebellum measured by MSD. Levels of soluble (upper panels) and aggregated (lower panels) mHTT as measured by Mesoscale Discovery (MSD) in (A) cortex, (B) hippocampus, (C) brain stem, and (D) cerebellum at 4, 9 and 15 weeks of age in R6/2, Sirt2HETxR6/2 (R6/2 HET) and Sirt2KOxR6/2 (R6/2 KO) mice. Soluble mHTT was measured with 2B7-MW1 and aggregated mHTT with MW8-MW8 antibody combinations. The signal for each tissue was expressed as fold change of R6/2 at 4 weeks of age. A signal was not observed above background for mice without the R6/2 transgene. n≥4/genotype/tissue/time point (except for R6/2 KO at 4 weeks where n = 3). Error bars represent SEM. *p≤0.05, **p≤0.01 (to the R6/2 at the respective time point). (TIF) [file pone.0034805.s006.tif]

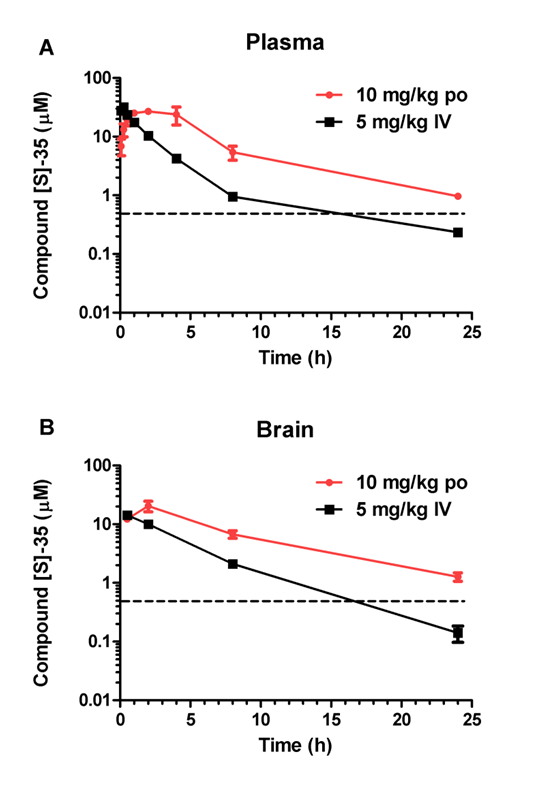

Supplement: Figure S7 — Pharmacokinetic properties of [S]-35 in mouse brain after acute dosing. Plasma and brain levels of [S]-35 following intravenous (IV), or oral gavage (PO) administration. Male, 6–7 weeks old C57BL/6 mice were dosed with 5 mg/kg (IV) or 10 mg/kg (PO) of [S]-35 and plasma and brains were harvested at various timepoints after compound administration. Concentration of [S]-35 in the analyte was measured by LC/MS/MS. n = 4 per timepoint, mean ± standard deviation shown. (TIF) [file pone.0034805.s007.tif]
