# Supplementary material for: SIRT2 Ablation Has No Effect on Tubulin Acetylation in Brain, Cholesterol Biosynthesis or the Progression of Huntington's Disease Phenotypes In Vivo
Source: PLoS One. 2012 Apr 12;7(4):e34805. doi: 10.1371/journal.pone.0034805 (PMC3325254; doi:10.1371/journal.pone.0034805)
Supplement: Table S1 — Genetic depletion of SIRT2 does not modify R6/2 exploratory behaviour. The p values displayed in Table S1 indicate whether the activity measures: activity, mobility, rearing and centre rearing are significantly influenced by R6/2 genotype, Sirt2 genotype, Sex and the duration of the 30 min test (Time) at 5, 7, 9, 11 and 13 weeks of age. Significant p-values are highlighted in blue for p<0.05, orange for p<0.01 and pink for p<0.001. (DOCX) [file pone.0034805.s008.docx]

|  | **Week** | **Activity** | **Mobility** | **Rearing** | **Centre Rearing** | p = < 0.001 |
| --- | --- | --- | --- | --- | --- | --- |
| **Time** | **5** | <0.001 | <0.001 | <0.001 | <0.001 | p = < 0.01 |
|  | **7** | <0.001 | <0.001 | <0.001 | <0.001 | p = < 0.05 |
|  | **9** | <0.001 | <0.001 | <0.001 | <0.001 |  |
|  | **11** | <0.001 | <0.001 | 0.008 | 0.006 |  |
|  | **13** | <0.001 | <0.001 | 0.013 | 0.004 |  |
| **R6/2 Genotype** | **5** | 0.212 | 0.044 | 0.708 | 0.655 |  |
|  | **7** | 0.055 | 0.005 | 0.061 | 0.557 |  |
|  | **9** | <0.001 | <0.001 | 0.487 | 0.910 |  |
|  | **11** | <0.001 | <0.001 | 0.147 | 0.004 |  |
|  | **13** | <0.001 | <0.001 | <0.001 | <0.001 |  |
| **Time*R6/2** | **5** | 0.041 | 0.043 | 0.134 | 0.025 |  |
|  | **7** | 0.012 | 0.058 | 0.167 | 0.243 |  |
|  | **9** | 0.697 | 0.561 | 0.036 | 0.158 |  |
|  | **11** | 0.078 | 0.039 | 0.352 | 0.464 |  |
|  | **13** | 0.248 | 0.300 | 0.060 | 0.375 |  |
| ***Sirt2*KO Genotype** | **5** | 0.651 | 0.685 | 0.160 | 0.314 |  |
|  | **7** | 0.663 | 0.766 | 0.669 | 0.874 |  |
|  | **9** | 0.133 | 0.243 | 0.158 | 0.216 |  |
|  | **11** | 0.660 | 0.565 | 0.954 | 0.136 |  |
|  | **13** | 0.938 | 0.933 | 0.195 | 0.168 |  |
| **Time**Sirt2*KO** | **5** | 0.143 | 0.293 | 0.054 | 0.063 |  |
|  | **7** | 0.607 | 0.545 | 0.366 | 0.094 |  |
|  | **9** | 0.735 | 0.756 | 0.763 | 0.794 |  |
|  | **11** | 0.469 | 0.240 | 0.222 | 0.271 |  |
|  | **13** | 0.204 | 0.350 | 0.191 | 0.395 |  |
| **Sex** | **5** | 0.536 | 0.556 | 0.129 | 0.166 |  |
|  | **7** | 0.598 | 0.381 | 0.059 | 0.117 |  |
|  | **9** | 0.185 | 0.161 | 0.081 | 0.496 |  |
|  | **11** | 0.090 | 0.406 | 0.309 | 0.165 |  |
|  | **13** | 0.346 | 0.423 | 0.535 | 0.726 |  |
| **Time*Sex** | **5** | 0.110 | 0.166 | 0.004 | 0.025 |  |
|  | **7** | <0.001 | <0.001 | 0.276 | 0.138 |  |
|  | **9** | 0.654 | 0.469 | 0.648 | 0.579 |  |
|  | **11** | 0.088 | 0.123 | 0.836 | 0.765 |  |
|  | **13** | 0.559 | 0.494 | 0.199 | 0.240 |  |
| **Sex*R6/2** | **5** | 0.846 | 0.952 | 0.865 | 0.670 |  |
|  | **7** | 0.277 | 0.569 | 0.518 | 0.783 |  |
|  | **9** | 0.298 | 0.755 | 0.043 | 0.009 |  |
|  | **11** | 0.012 | 0.032 | 0.007 | 0.001 |  |
|  | **13** | 0.194 | 0.319 | 0.071 | 0.165 |  |
|  | | | | | | |
| **Sex**Sirt2*KO** | **5** | 0.071 | 0.147 | 0.004 | 0.056 |  |
|  | **7** | 0.395 | 0.440 | 0.477 | 0.803 |  |
|  | **9** | 0.496 | 0.575 | 0.645 | 0.286 |  |
|  | **11** | 0.209 | 0.209 | 0.055 | 0.040 |  |
|  | **13** | 0.909 | 0.836 | 0.094 | 0.206 |  |
| **Sex*R6/2**Sirt2*KO** | **5** | 0.908 | 0.904 | 0.335 | 0.804 |  |
|  | **7** | 0.706 | 0.704 | 0.772 | 0.575 |  |
|  | **9** | 0.600 | 0.506 | 0.449 | 0.428 |  |
|  | **11** | 0.079 | 0.078 | 0.259 | 0.524 |  |
|  | **13** | 0.423 | 0.665 | 0.505 | 0.683 |  |
| **R6/2**Sirt2*KO** | **5** | 0.431 | 0.391 | 0.279 | 0.872 |  |
|  | **7** | 0.836 | 0.894 | 0.151 | 0.201 |  |
|  | **9** | 0.546 | 0.662 | 0.640 | 0.688 |  |
|  | **11** | 0.800 | 0.648 | 0.652 | 0.729 |  |
|  | **13** | 0.934 | 0.840 | 0.483 | 0.855 |  |
| **Time*R6/2**Sirt2KO*** | **5** | 0.436 | 0.640 | 0.549 | 0.617 |  |
|  | **7** | 0.008 | 0.026 | 0.043 | 0.064 |  |
|  | **9** | 0.598 | 0.579 | 0.282 | 0.148 |  |
|  | **11** | 0.789 | 0.890 | 0.690 | 0.821 |  |
|  | **13** | 0.812 | 0.893 | 0.293 | 0.632 |  |
| **Time*Sex*R6/2* *Sirt2*KO** | **5** | 0.660 | 0.587 | 0.562 | 0.186 |  |
|  | **7** | 0.232 | 0.222 | 0.225 | 0.040 |  |
|  | **9** | 0.683 | 0.554 | 0.672 | 0.780 |  |
|  | **11** | 0.516 | 0.423 | 0.382 | 0.849 |  |
|  | **13** | 0.457 | 0.603 | 0.422 | 0.818 |  |

**Table S1 Genetic depletion of *Sirt2* does not modify R6/2 exploratory behaviour**

The *p* values displayed in Table S1 indicate whether the activity measures: activity, mobility rearing and centre rearing are significantly influenced by R6/2 genotype, *Sirt2* genotype, Sex and the duration of the 30 min test (Time) at 5, 7, 9, 11 and 13 weeks of age. Significant *p*-values are highlighted in blue for *p*<0.05, orange for *p*<0.01 and pink for *p*<0.001.
