# Supplementary material for: SIRT2 Ablation Has No Effect on Tubulin Acetylation in Brain, Cholesterol Biosynthesis or the Progression of Huntington's Disease Phenotypes In Vivo
Source: PLoS One. 2012 Apr 12;7(4):e34805. doi: 10.1371/journal.pone.0034805 (PMC3325254; doi:10.1371/journal.pone.0034805)
Supplement: Table S2 — Summary of primer and probe sequences designed in-house. (DOCX) [file pone.0034805.s009.docx]

**Table S2.** **Summary of primer and probe sequences designed in-house.**

| Name | Application | 5’ – 3’ sequence |
| --- | --- | --- |
| *Hdac1* forward | RT-qPCR | TCTGAATACAGCAAGCAGATGCA |
| *Hdac1* reverse | RT-qPCR | ACAGAACTCAAACAAGCCATCAAAC |
| *Hdac1* probe | RT-qPCR | AGATTCAATGTTGGTGAGGACTGTCCGG |
| *Hdac2* forward | RT-qPCR | AGAAGATTGTCCGGTGTTTGATG |
| *Hdac2* reverse | RT-qPCR | CACAGCCCCAGCAACTGAA |
| *Hdac2* probe | RT-qPCR | TTGAGTTTTGTCAGCTCTCCACGGGTG |
| *Hdac3* forward | RT-qPCR | TCAGCCCCACCAATATGCA |
| *Hdac3* reverse | RT-qPCR | GAACTCGAAAAGTCCTGGAAACA |
| *Hdac3* probe | RT-qPCR | CCTTAATGCCTTCAACGTGGGT |
| *Hdac4* forward | RT-qPCR | CTGGCATCCCTGTGTCATTTG |
| *Hdac4* reverse | RT-qPCR | ACACAAGACCTGTGGTGAACCTT |
| *Hdac4* probe | RT-qPCR | CTGCCACCTTCCCCATGTCAGTCC |
| *Hdac5* forward | RT-qPCR | CCAGAGCCGGCATAACT |
| *Hdac5* reverse | RT-qPCR | GCAGGATTTCCAAGATGGTT |
| *Hdac5* probe | RT-qPCR | TCCCAACGAGTCGGATGGCATGT |
| *Hdac6* forward | RT-qPCR | GGAGACAACCCAGTACATGAATGAA |
| *Hdac6* reverse | RT-qPCR | CGGAGGACAGAGCCTGTAG |
| *Hdac6* probe | RT-qPCR | T ATCTGCATCC GAACTCATAT TCCTGTGCCTG |
| *Hdac7* forward | RT-qPCR | CCCACCTGTCAGACCCAAGT |
| *Hdac7* reverse | RT-qPCR | AGTCATAGACCAGCCCTGTAGCA |
| *Hdac7* probe | RT-qPCR | CTCAACAGCTCAGAGACA |
| *Hdac8* forward | RT-qPCR | GGCCCATCCATCCCTGTAG |
| *Hdac8* reverse | RT-qPCR | TTTAGATCGCCGGAGACAGTTT |
| *Hdac8* probe | RT-qPCR | TGGACGAGGGACCAGG |
| *Hdac9* forward | RT-qPCR | TGGCAGAATCCTCGGTCAGT |
| *Hdac9* reverse | RT-qPCR | CCCAGCAGGGCCATTGT |
| *Hdac9* probe | RT-qPCR | TCTCCAGGGTCAGGTCCCAGTTCACC |
| *Hdac10* forward | RT-qPCR | CCGCTATGAGCATGGAAGCT |
| *Hdac10* reverse | RT-qPCR | CAACTGCATCTGCATCAGACTCT |
| *Hdac10* probe | RT-qPCR | CTGGCCGTTTCTC |
| *Hdac11* forward | RT-qPCR | TGGGCATGAGCGAGACTTC |
| *Hdac11* reverse | RT-qPCR | GCGGTTGTAAACATCCATGATG |
| *Hdac11* probe | RT-qPCR | TGGGTGACAAGCGAG |
| *Sirt1* forward | RT-qPCR | TGTTGGTTGACTTCATCTTCCTT |
| *Sirt1* reverse | RT-qPCR | TCCAATGGCTTTTGAAAACTTTA |
| *Sirt1* probe | RT-qPCR | TTCATTTGTATGATACATTCGTATGTATG |
| *Sirt2* forward | RT-qPCR | TCCTGCAGAAAAGAATACACGAT |
| *Sirt2* reverse | RT-qPCR | CGATATCAGGCTTTACCACACTC |
| *Sirt2* probe | RT-qPCR | AGAGAAGATCTTCTCAGAAGCAACTCC |
| *Sirt3* forward | RT-qPCR | ACAAGAACTGCTGGATCTTATGC |
| *Sirt3* reverse | RT-qPCR | TCTTGCTGGACATAGGATGATCT |
| *Sirt3* probe | RT-qPCR | ACGTGGCAAGCTG GATGGACAGGA |
| *Sirt5* forward | RT-qPCR | CAGAGGCGCTGGAGGTTACTGGAG |
| *Sirt5* reverse | RT-qPCR | CCGGGCTTCACACTGGGCAATGGC |
| *Sirt5* probe | RT-qPCR | AGGCTCAGGACCTGGCAACCCCTCAG |
| *Sirt7* forward | RT-qPCR | GCCTCCCTCTTTCTACTCCTTATC |
| *Sirt7* reverse | RT-qPCR | TGCTCAGACTGGAGGCTTAGTTA |
| *Sirt7* probe | RT-qPCR | TACAAGTGTTCACTTTATAGAAGCCT |
| *Sirt2* forward | Genotyping/  Sequencing | CAGGTGTGAGCAGTGTCAGAGTG |
| *Sirt2* reverse KO | Genotyping/  Sequencing | GACGTCGAGGTGCCCGAAGGACC |
| *Sirt2* reverse WT | Genotyping/  Sequencing | CCAGCCCAGAGTGGACACT |
| *Sirt2* forward Seq 2 | Sequencing | TCGTGCGCTCCTTTCGGTC |
| *Sirt2* forward Seq 3 | Sequencing | TCAACTCGGCCATGCGCGGG |

KEY: RT-qPCR - real time - quantitative PCR; Primer and probe mixes for *ActB, Atp5b*, *Canx*, *Gapdh,* *Rpl13a* and *Ubc* RT-qPCR assays were purchased from Primer Design. All RT-qPCR probes were labelled 5’ with FAM and 3’ with TAMRA.
