# Supplementary material for: SIRT2 Ablation Has No Effect on Tubulin Acetylation in Brain, Cholesterol Biosynthesis or the Progression of Huntington's Disease Phenotypes In Vivo
Source: PLoS One. 2012 Apr 12;7(4):e34805. doi: 10.1371/journal.pone.0034805 (PMC3325254; doi:10.1371/journal.pone.0034805)
Supplement: Table S3 — Summary of all antibodies used in this study. (DOCX) [file pone.0034805.s010.docx]

**Table S3. Summary of all antibodies used in this study.**

| Antibody | Catalogue number | Source | Dilution/ amount | Application |
| --- | --- | --- | --- | --- |
|  |  |  |  |  |
| S829 | N/A | In House | 1:1000 | WB |
| MW8 | N/A | P.Patterson | 1:5000 | SEP |
| MW8 | N/A | P.Patterson | 10 ng in 6µl | TR-FRET |
| MW8 | N/A | P.Patterson | 1ng in 6µl | TR-FRET |
| MW8 | N/A | P.Patterson | 10 µg/ml | MSD |
| MW8-Sulfo Tag | N/A | P.Patterson | 1 µg/ml | MSD |
| 2B7 | N/A | Novartis | 10 ng in 6µl | TR-FRET |
| 2B7 | N/A | Novartis | 30 µg/ml | MSD |
| MW1 | N/A | P.Patterson | 1ng in 6µl | TR-FRET |
| MW1-Sulfo Tag | N/A | P.Patterson | 10 µg/ml | MSD |
| SIRT2 | sc-20966 | Santa Cruz | 1:1000 | WB |
| SIRT2 | S8847 | Sigma | 1:1000 | WB |
| SIRT1 | 2028 | Cell Signalling | 1:1000 | WB |
| SREBP-2 | ab30682 | Abcam | 1:1000 | WB |
| Ac-H4K16 | 07-329 | Millipore | 1:1000 | WB |
| Ac-tubulin | T7451 | Sigma | 1:40 000 | WB |
| Histone H4 | 04-858 | Millipore | 1:5000 | WB |
| Histone H3 | ab1791 | Abcam | 1:30 000 | WB |
| α-tubulin | T9026 | Sigma | 1:30 000 | WB |
| Actin | sc-47778 | Sigma | 1:40 000 | WB |
| anti-Goat HRP | P044901 | Dako | 1:5000 | WB |
| anti-Mouse HRP | P0260 | Dako | 1:5000 | WB |
| anti-Rabbit HRP | 32460 | Pierce | 1:20 000 | WB |

Key: WB Western blotting, SEP Seprion ligand ELISA, TR-FRET time resolved Förster resonance energy transfer, MSD Mesoscale Discovery, HRP horse radish peroxidise conjugated.
