# Supplementary figures and images for: Correction: SIRT2 Ablation Has No Effect on Tubulin Acetylation in Brain, Cholesterol Biosynthesis or the Progression of Huntington’s Disease Phenotypes In Vivo
Source: PLoS One. 2021 Mar 25;16(3):e0248926. doi: 10.1371/journal.pone.0248926 (PMC7993610; doi:10.1371/journal.pone.0248926)

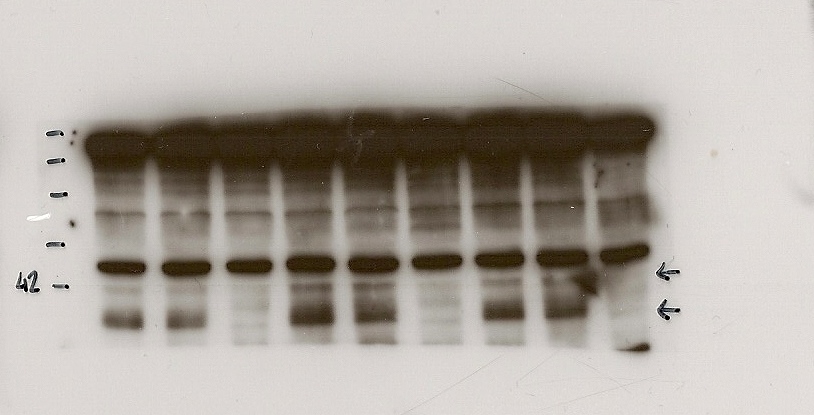

Supplement: S1 File — (ZIP) [file pone.0248926.s001.zip › S1/Fig1_C_H95_SIRT2.jpg]

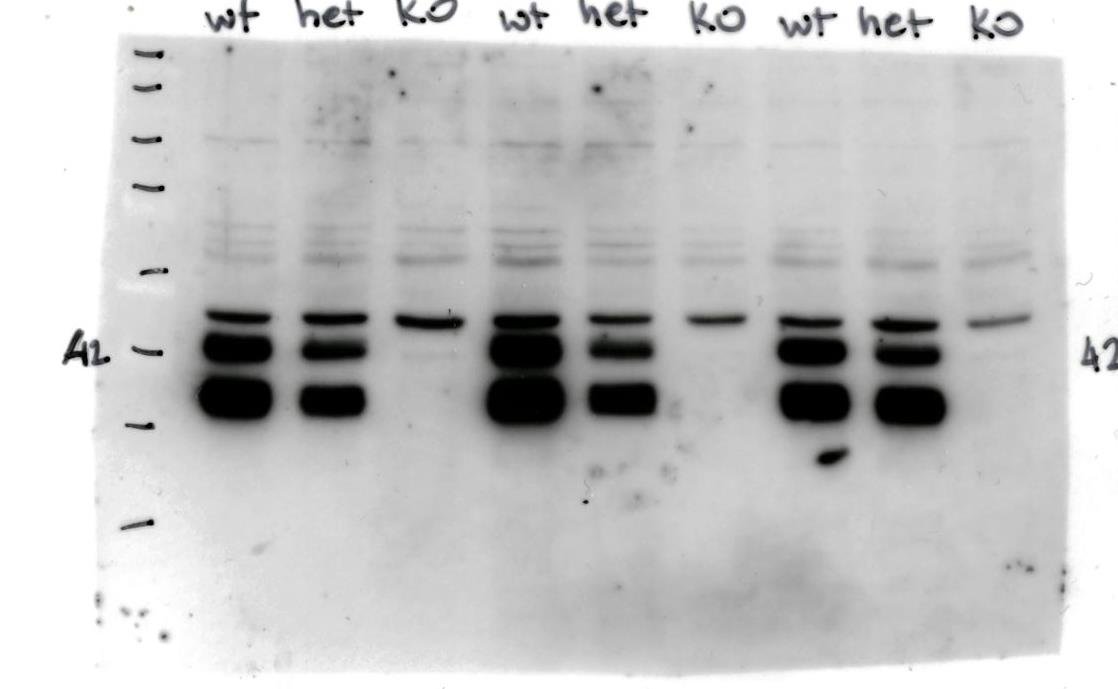

Supplement: S1 File — (ZIP) [file pone.0248926.s001.zip › S1/Fig1_C_S8447_SIRT2.jpg]

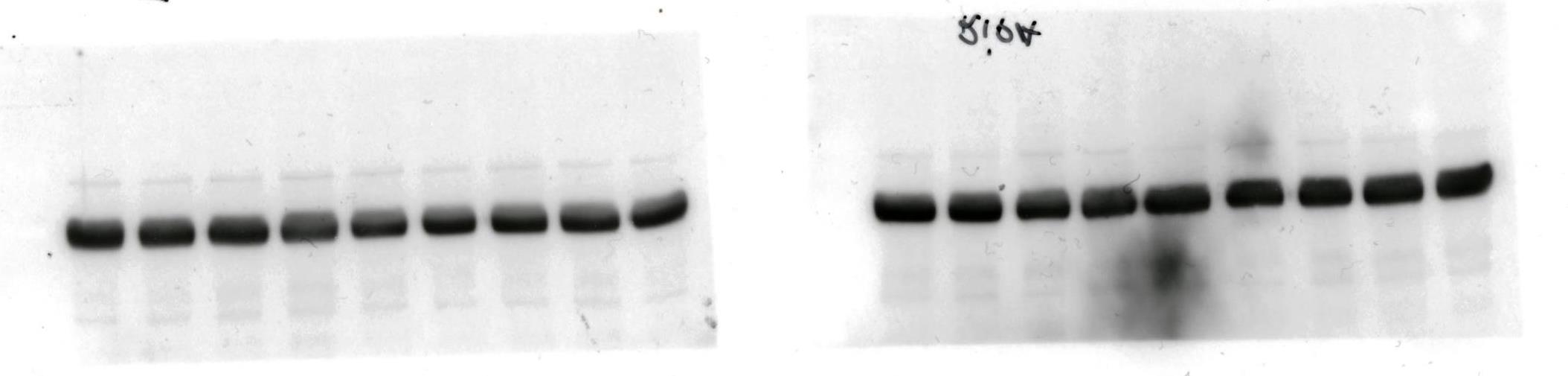

Supplement: S1 File — (ZIP) [file pone.0248926.s001.zip › S1/Fig1_C_Tubulin_H95(left)_S8447(right).jpg]

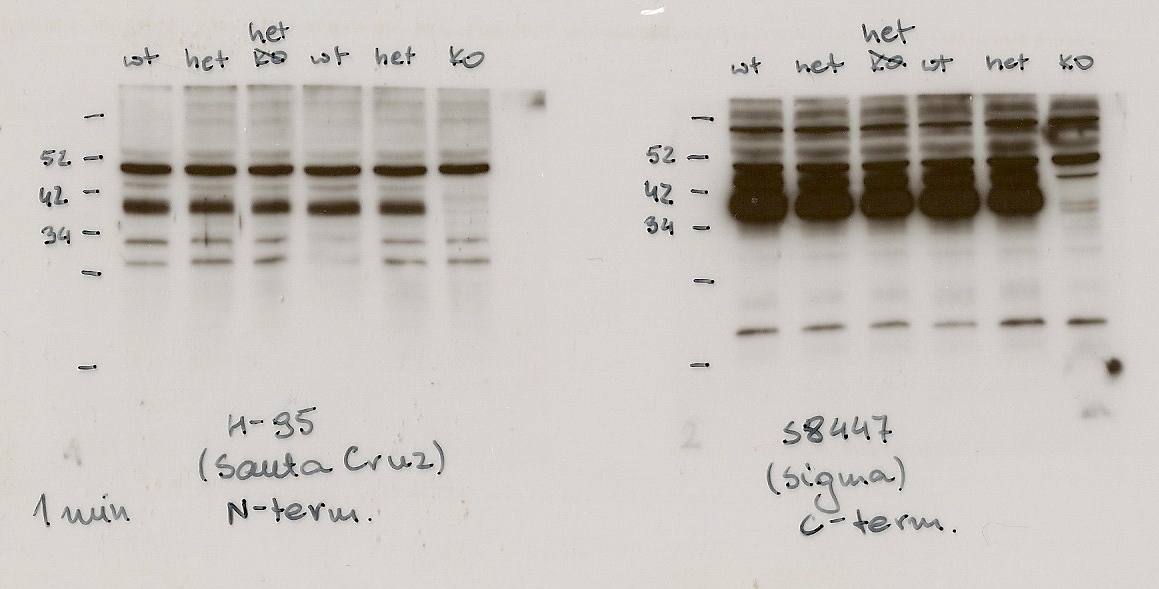

Supplement: S1 File — (ZIP) [file pone.0248926.s001.zip › S1/Fig1_D_H95_SIRT2.jpg]

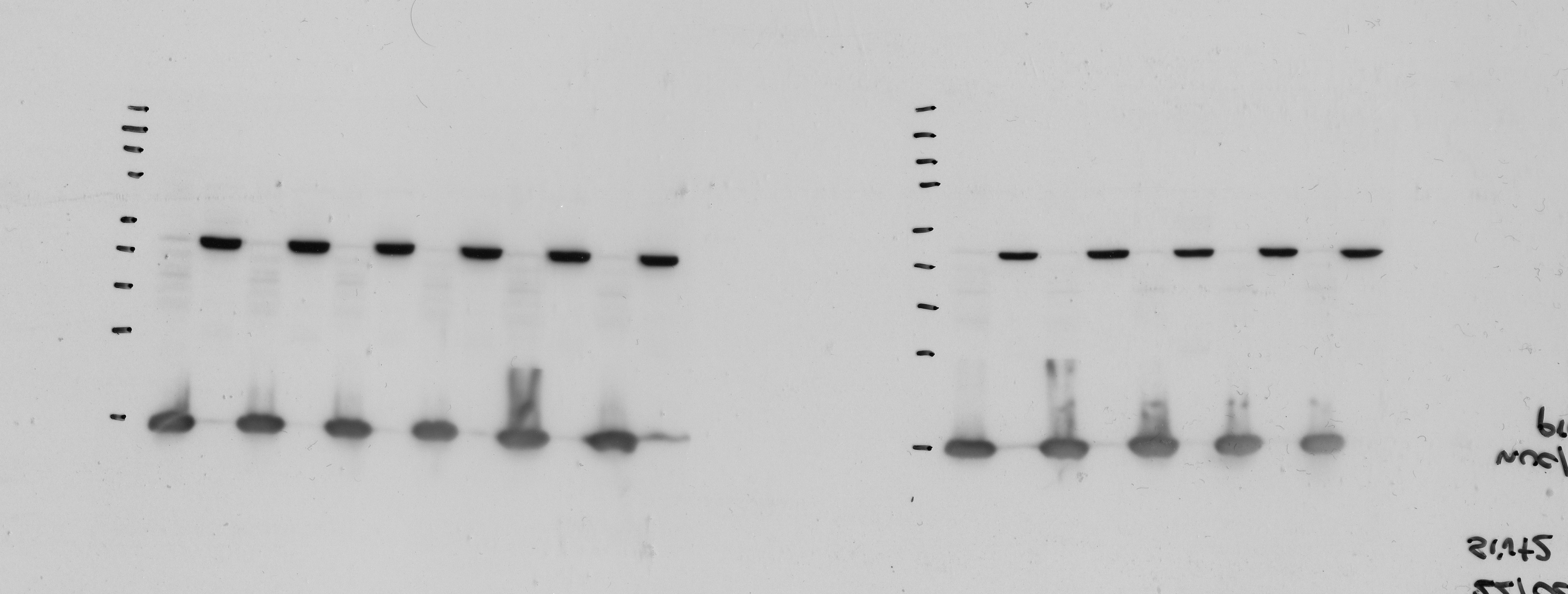

Supplement: S1 File — (ZIP) [file pone.0248926.s001.zip › S1/Fig1_E_Actin_H3.jpg]

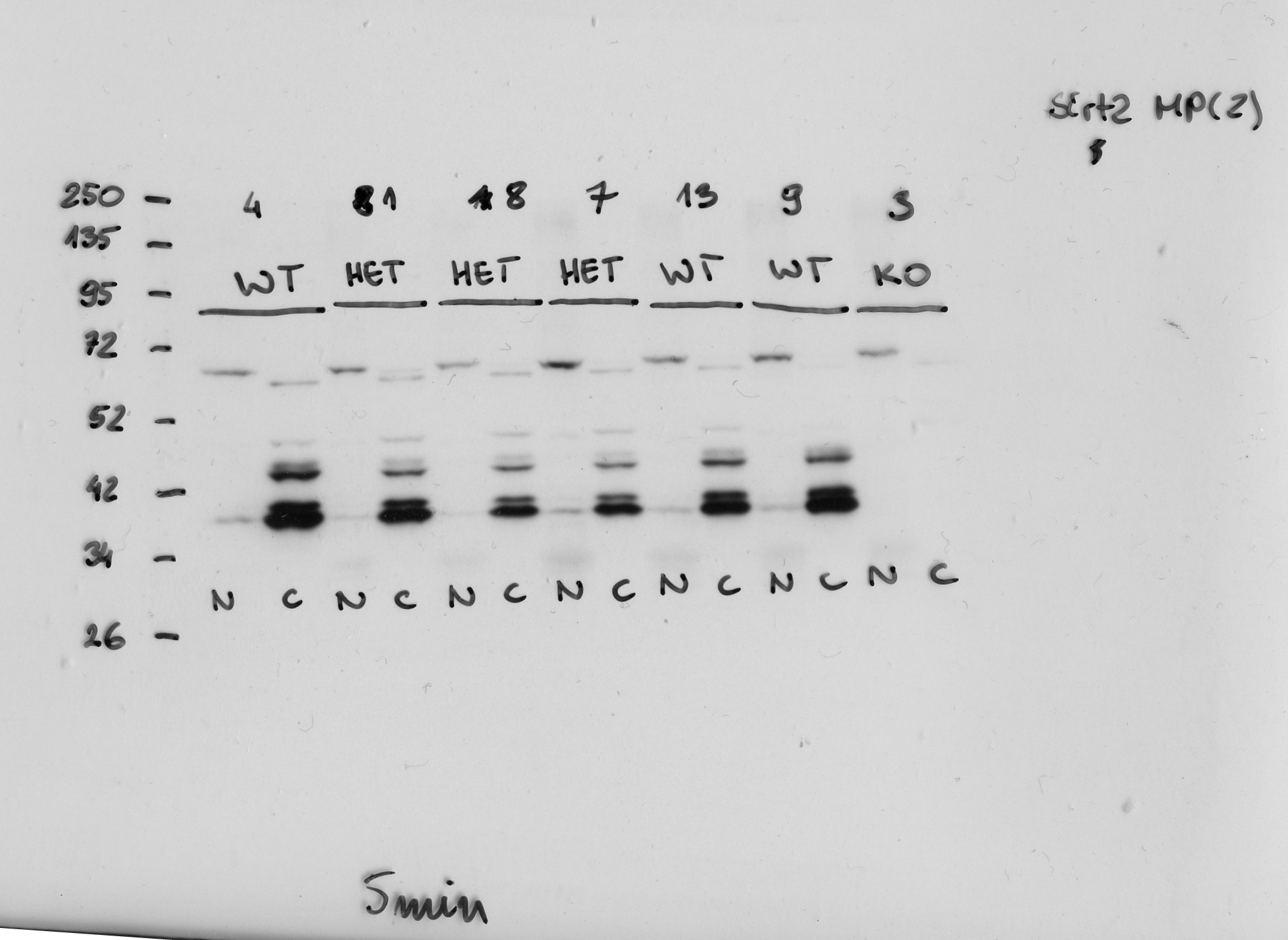

Supplement: S1 File — (ZIP) [file pone.0248926.s001.zip › S1/Fig1_E_SIRT2.jpg]

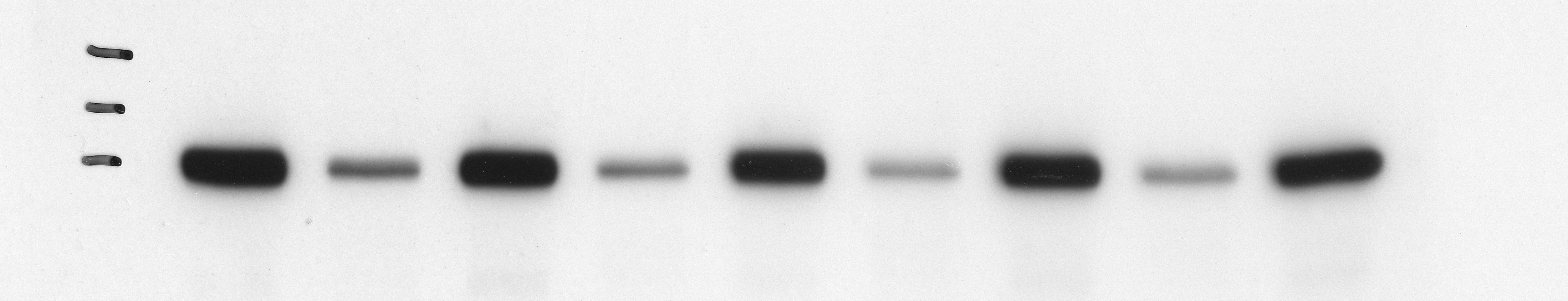

Supplement: S1 File — (ZIP) [file pone.0248926.s001.zip › S1/Fig2_A_HDAC6_Cerebellum_AcTub.jpg]

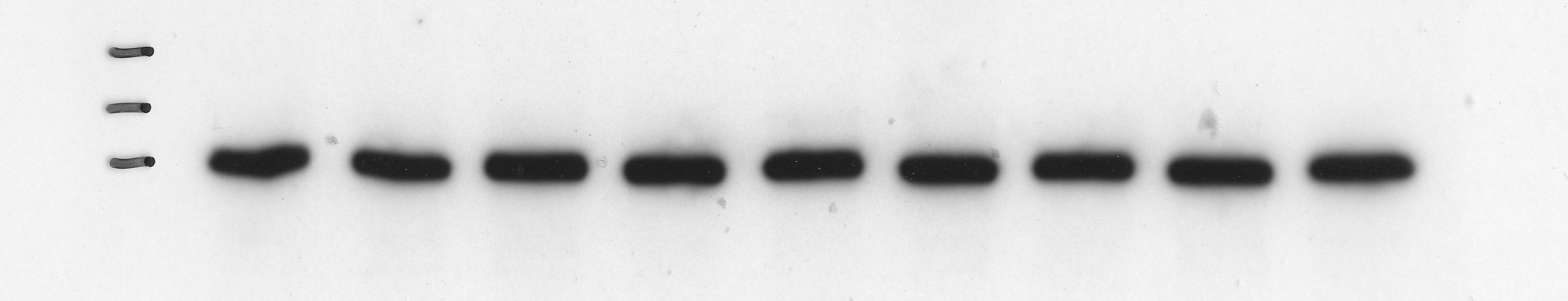

Supplement: S1 File — (ZIP) [file pone.0248926.s001.zip › S1/Fig2_A_HDAC6_Cerebellum_Tub.jpg]

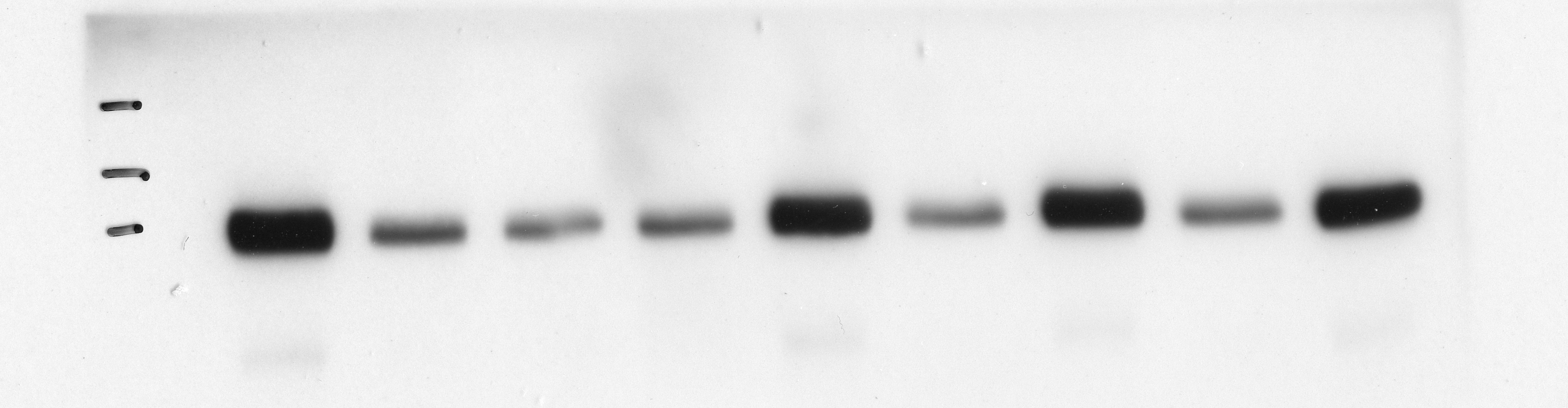

Supplement: S1 File — (ZIP) [file pone.0248926.s001.zip › S1/Fig2_A_HDAC6_Cortex_AcTub.jpg]

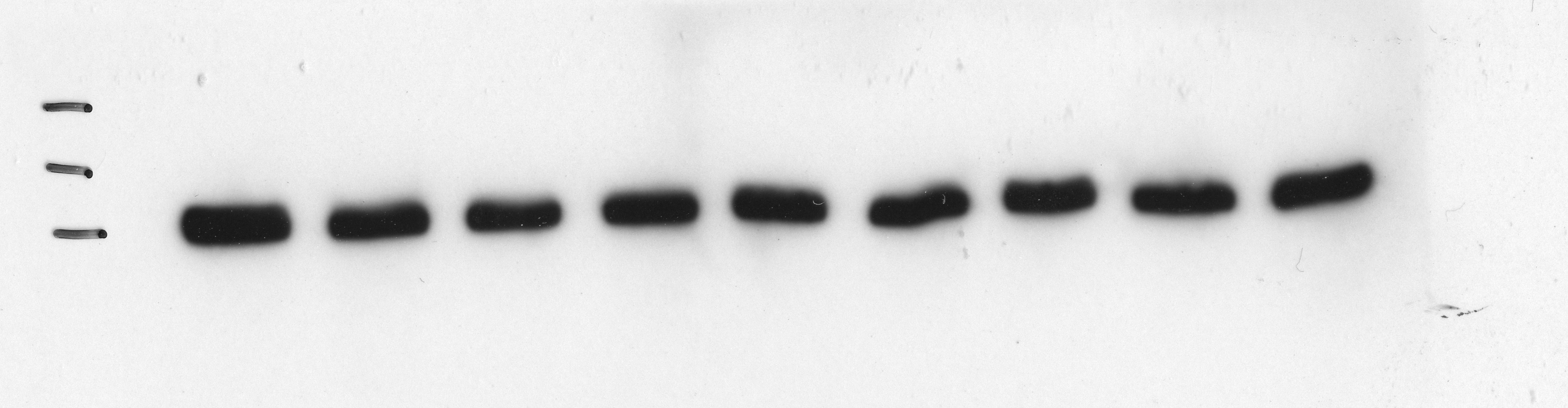

Supplement: S1 File — (ZIP) [file pone.0248926.s001.zip › S1/Fig2_A_HDAC6_Cortex_Tub.jpg]

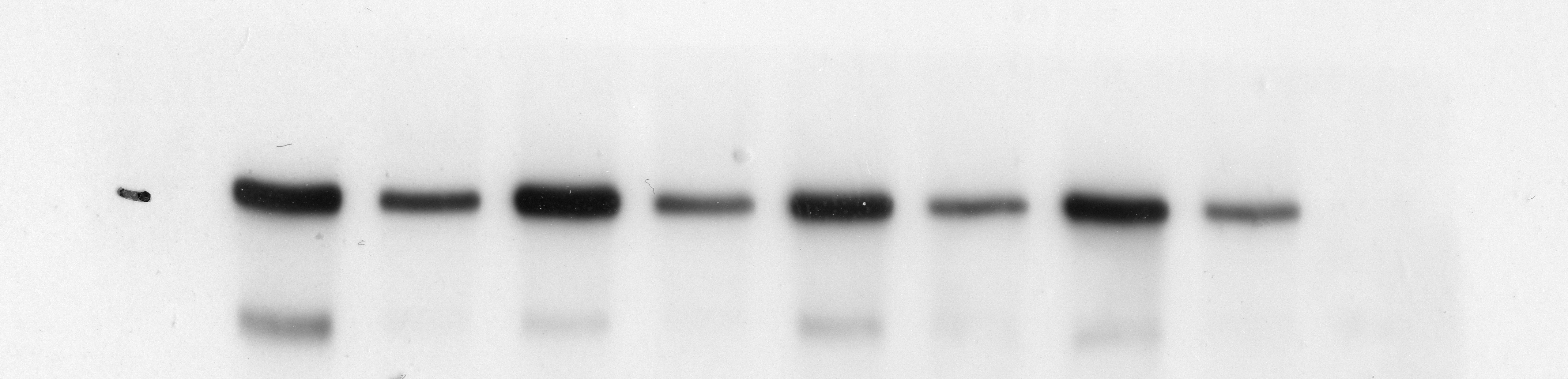

Supplement: S1 File — (ZIP) [file pone.0248926.s001.zip › S1/Fig2_A_HDAC6_Striatum_AcTub.jpg]

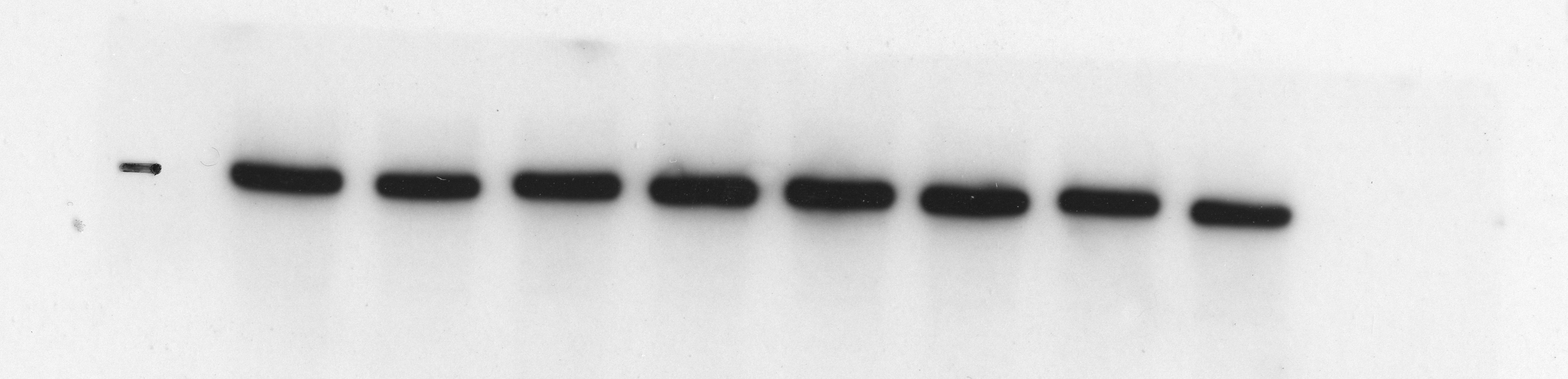

Supplement: S1 File — (ZIP) [file pone.0248926.s001.zip › S1/Fig2_A_HDAC6_Striatum_Tub.jpg]

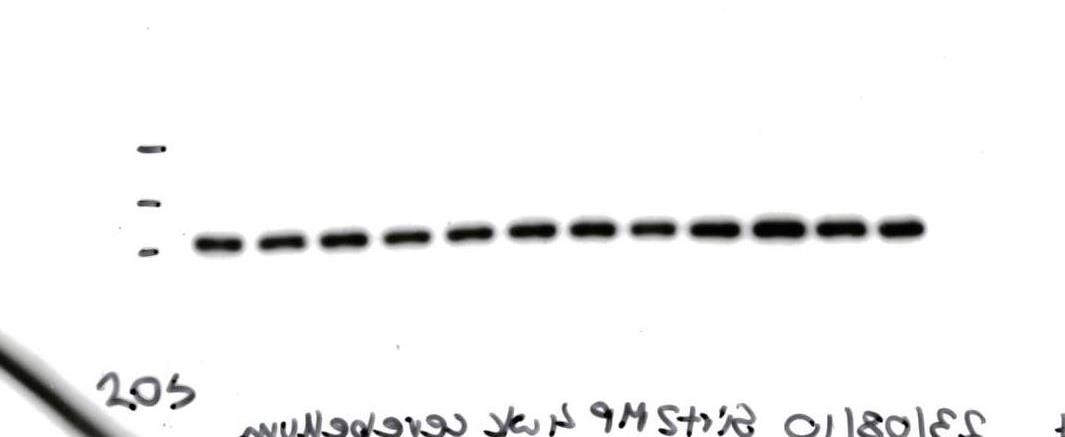

Supplement: S1 File — (ZIP) [file pone.0248926.s001.zip › S1/Fig2_A_SIRT2_Cerebellum_AcTub.jpg]

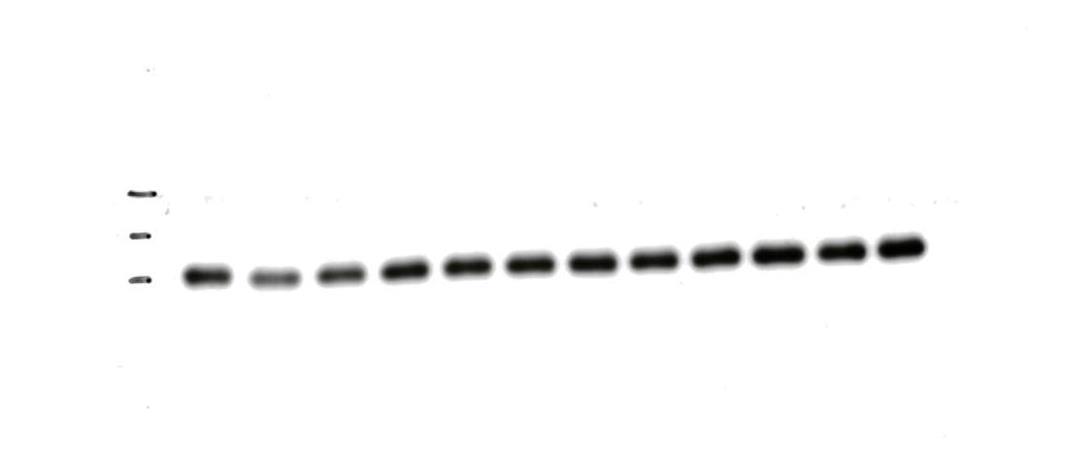

Supplement: S1 File — (ZIP) [file pone.0248926.s001.zip › S1/Fig2_A_SIRT2_Cerebellum_Tub.jpg]

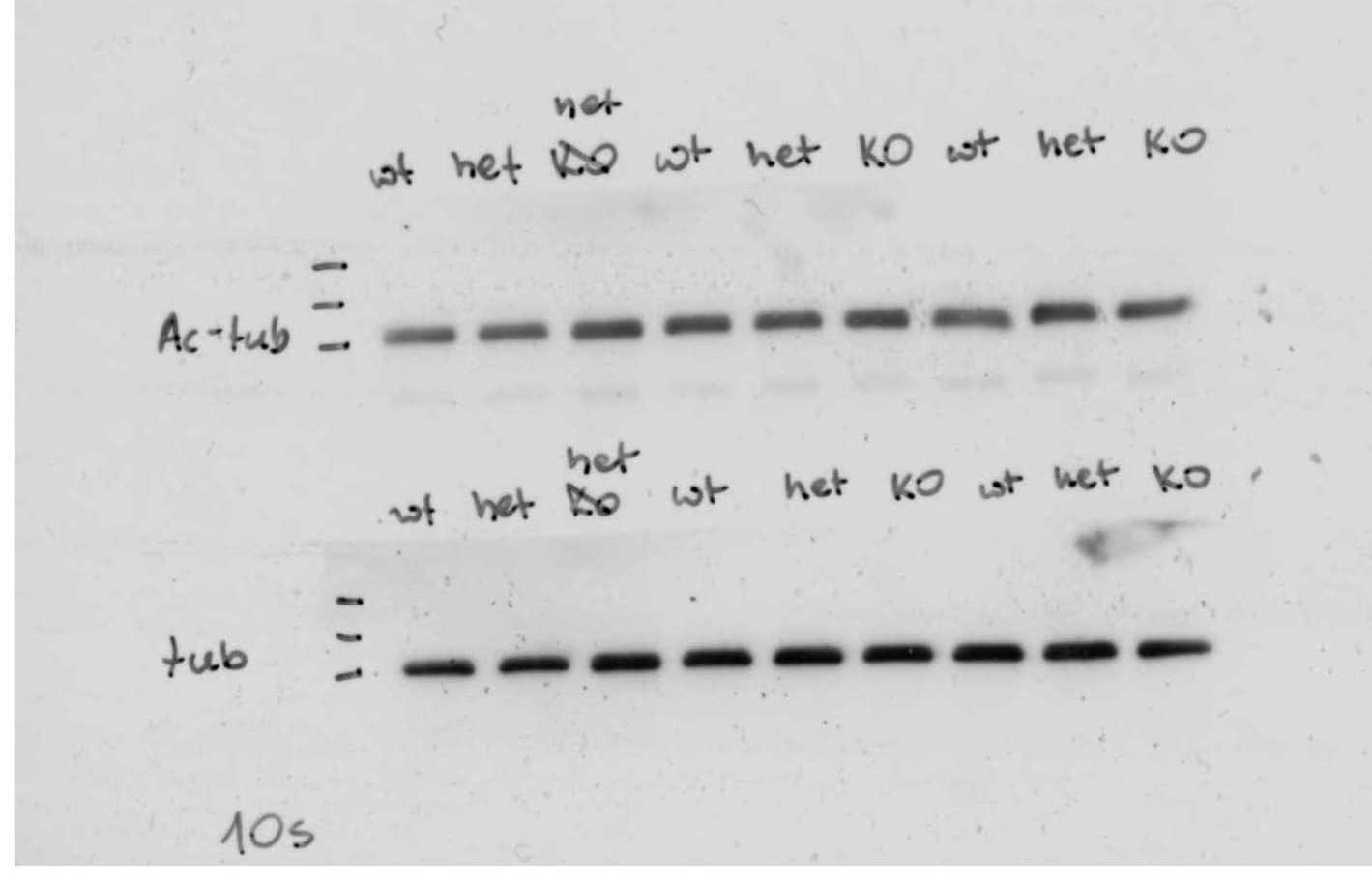

Supplement: S1 File — (ZIP) [file pone.0248926.s001.zip › S1/Fig2_A_SIRT2_Cortex_AcTub_Tub.jpg]

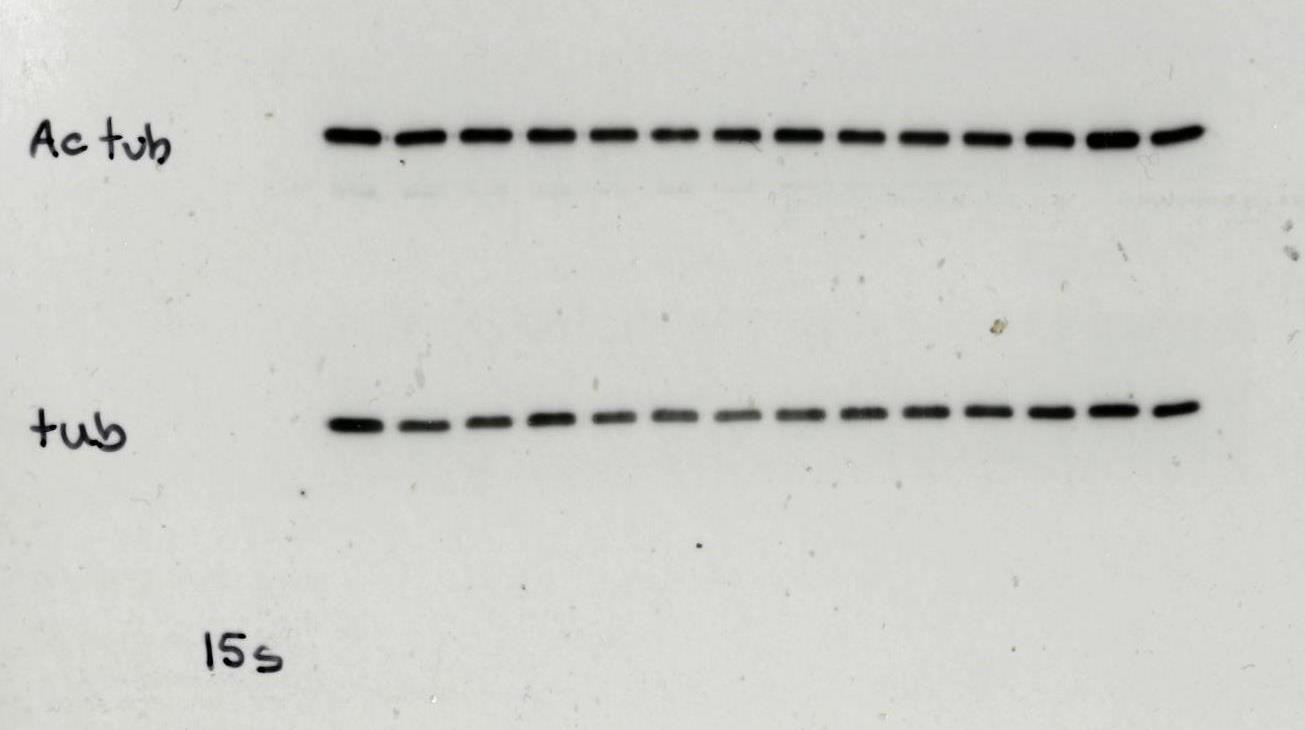

Supplement: S1 File — (ZIP) [file pone.0248926.s001.zip › S1/Fig2_A_SIRT2_Striatum_AcTub_Tub.jpg]

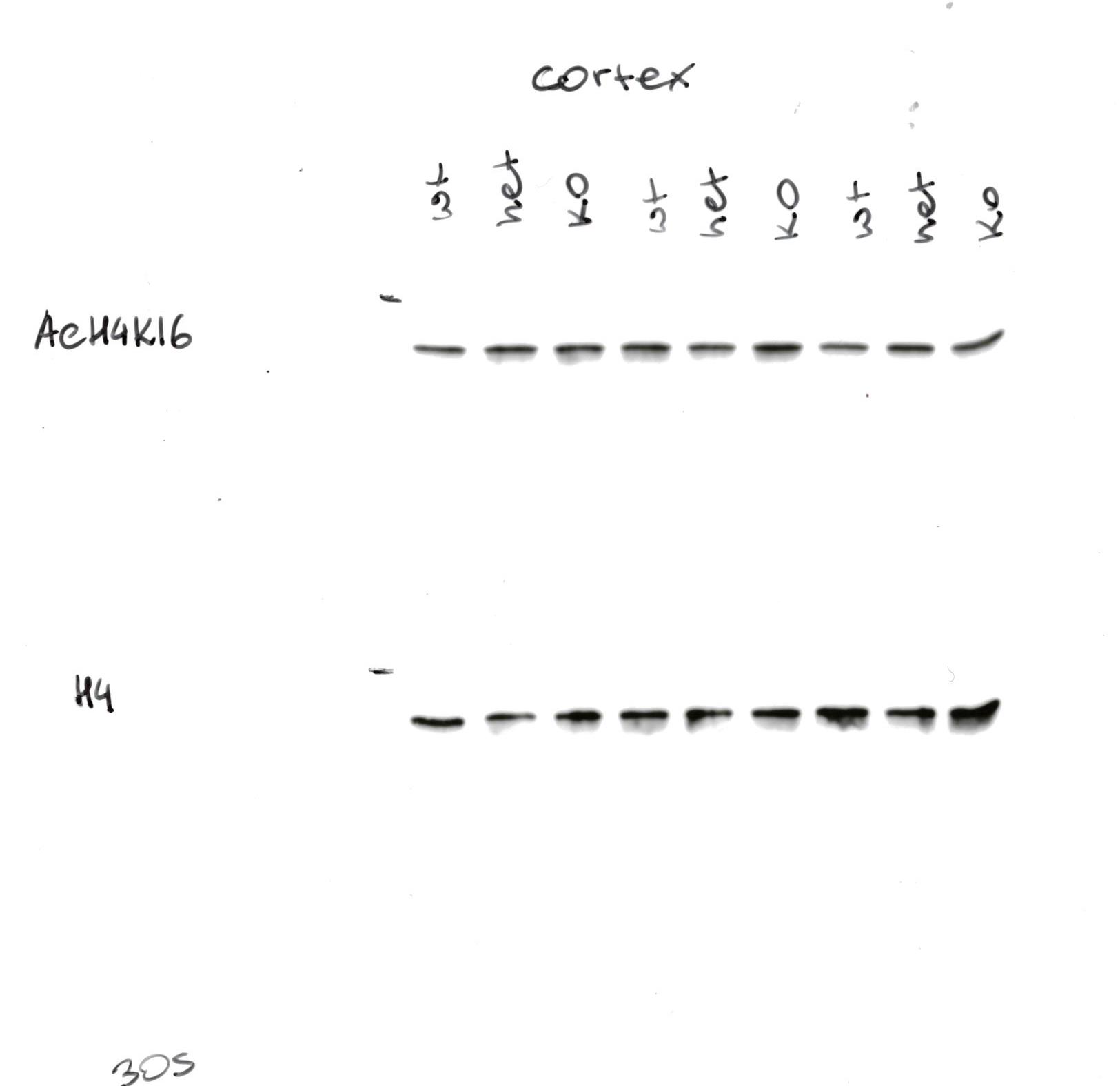

Supplement: S1 File — (ZIP) [file pone.0248926.s001.zip › S1/Fig2_C_Cortex_AcH4K16_H4.jpg]

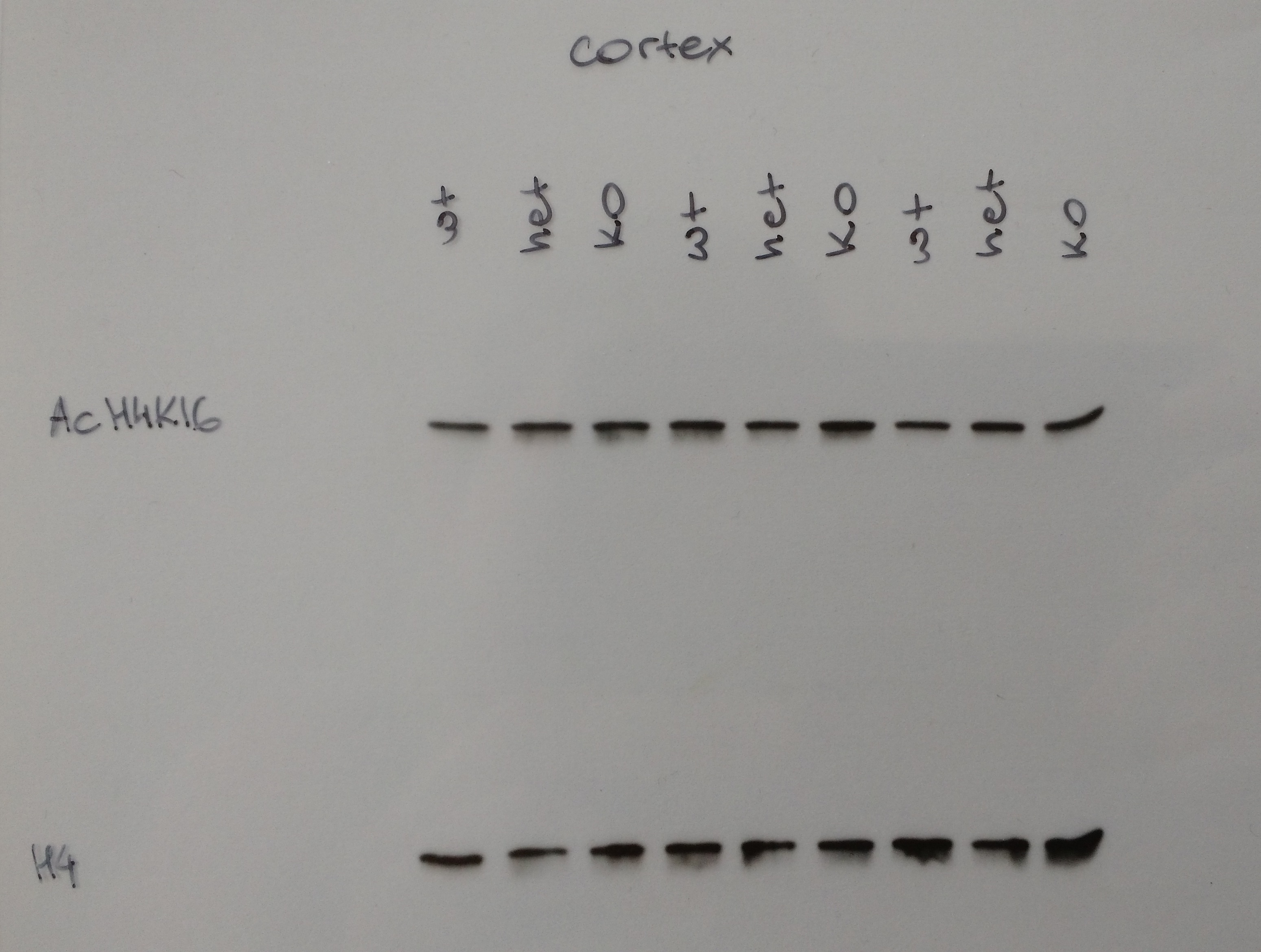

Supplement: S1 File — (ZIP) [file pone.0248926.s001.zip › S1/Fig2_C_Cortex_AcH4K16_H4_phonePic.jpg]

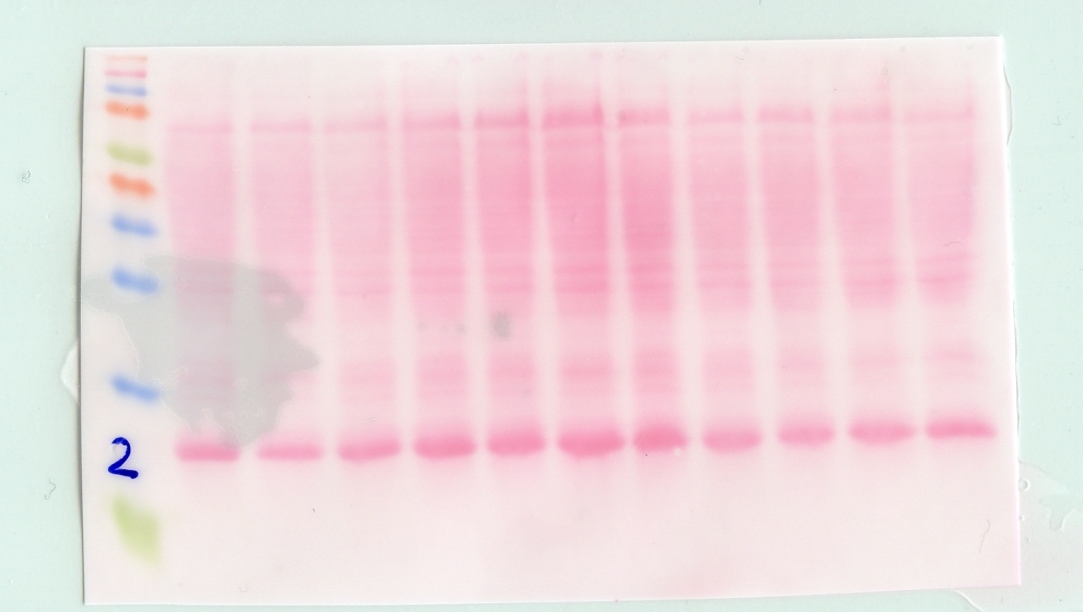

Supplement: S1 File — (ZIP) [file pone.0248926.s001.zip › S1/Fig2_C_Liver_H4.jpg]

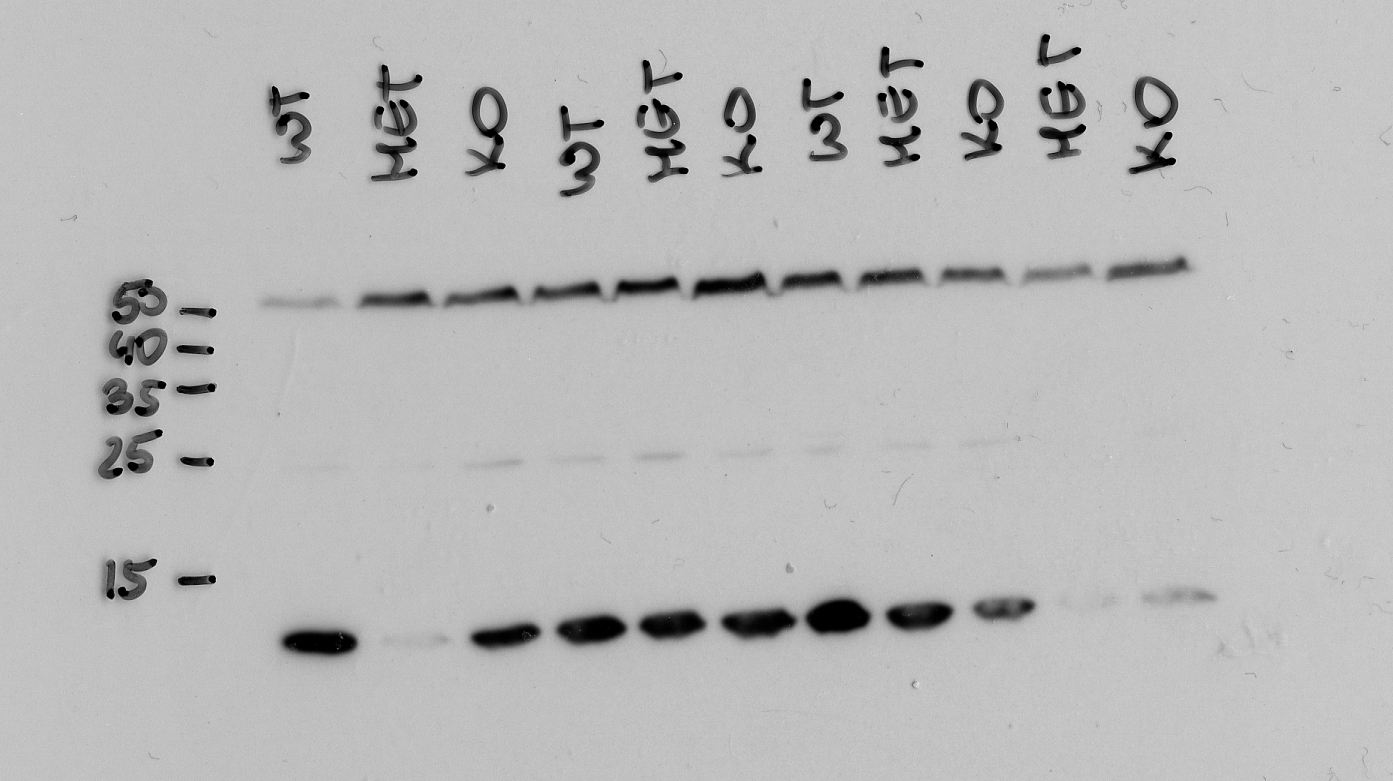

Supplement: S1 File — (ZIP) [file pone.0248926.s001.zip › S1/Fig2_C_Liver_H4K16Ac.jpg]

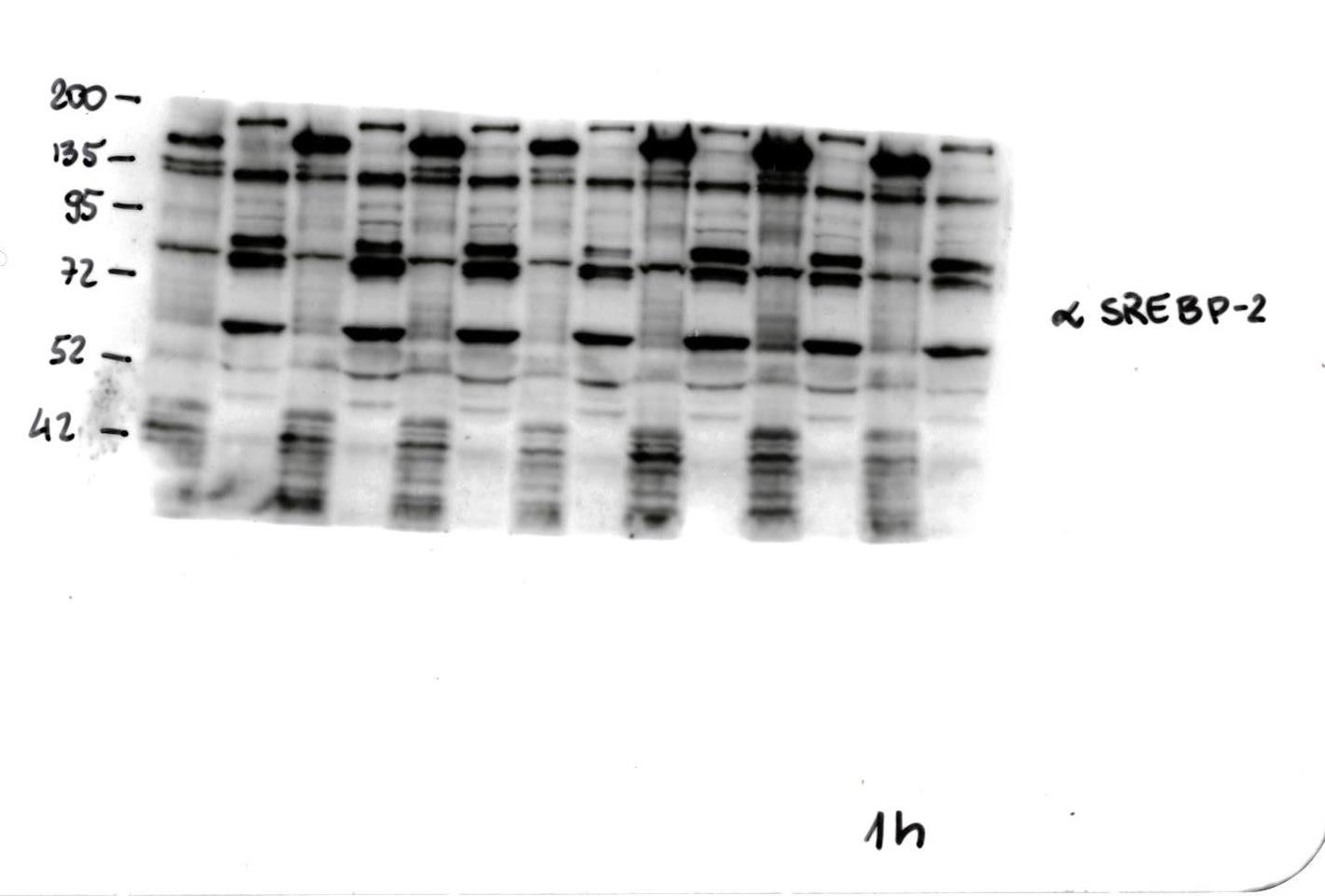

Supplement: S1 File — (ZIP) [file pone.0248926.s001.zip › S1/Fig2_H_SREBP2.jpg]

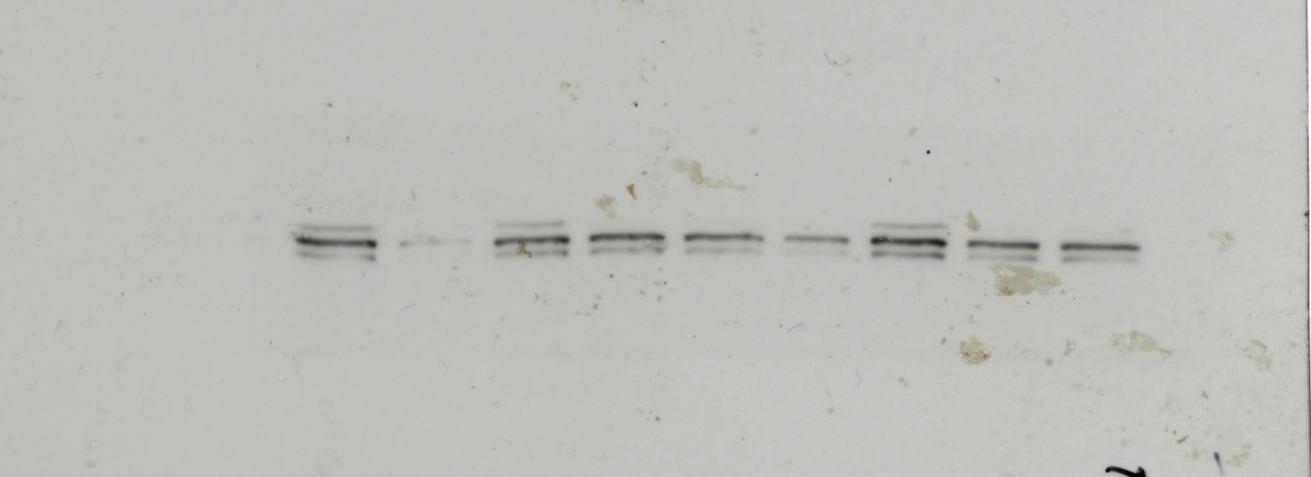

Supplement: S1 File — (ZIP) [file pone.0248926.s001.zip › S1/FigS2_C_SIRT1_1.jpg]

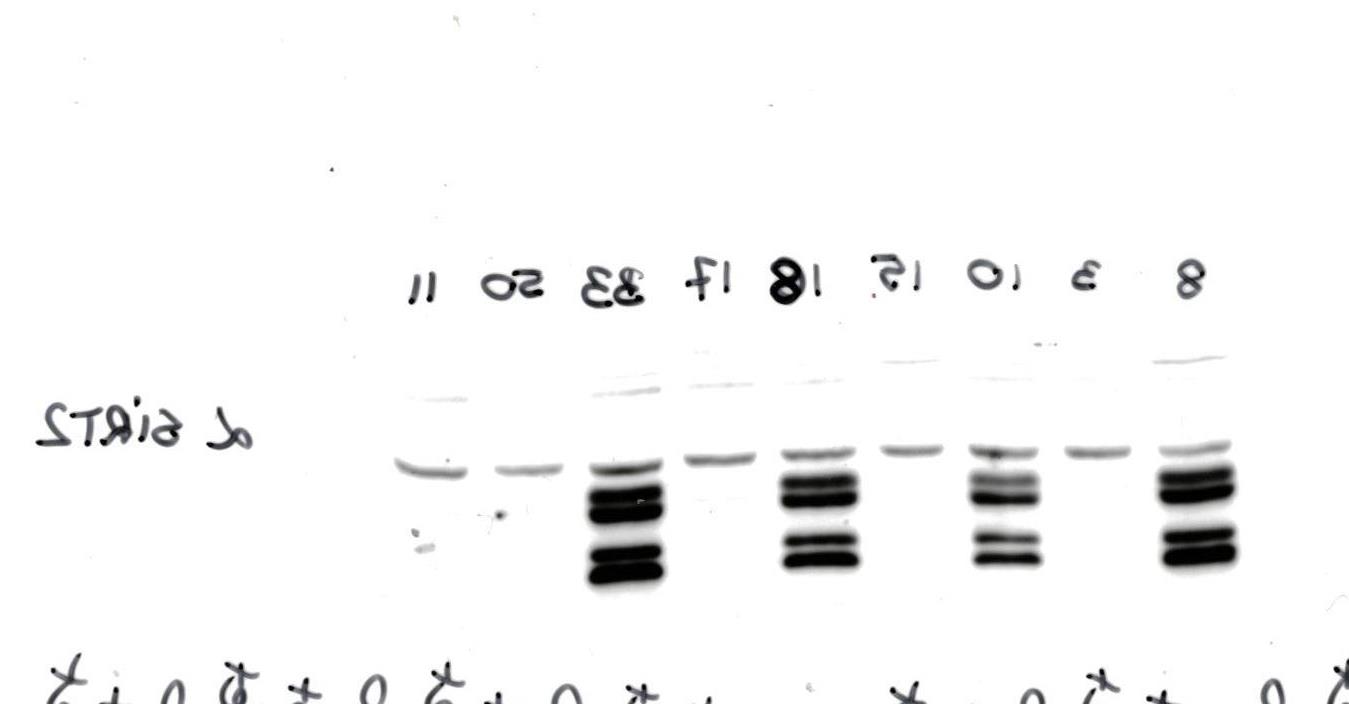

Supplement: S1 File — (ZIP) [file pone.0248926.s001.zip › S1/FigS2_C_SIRT2_2.jpg]

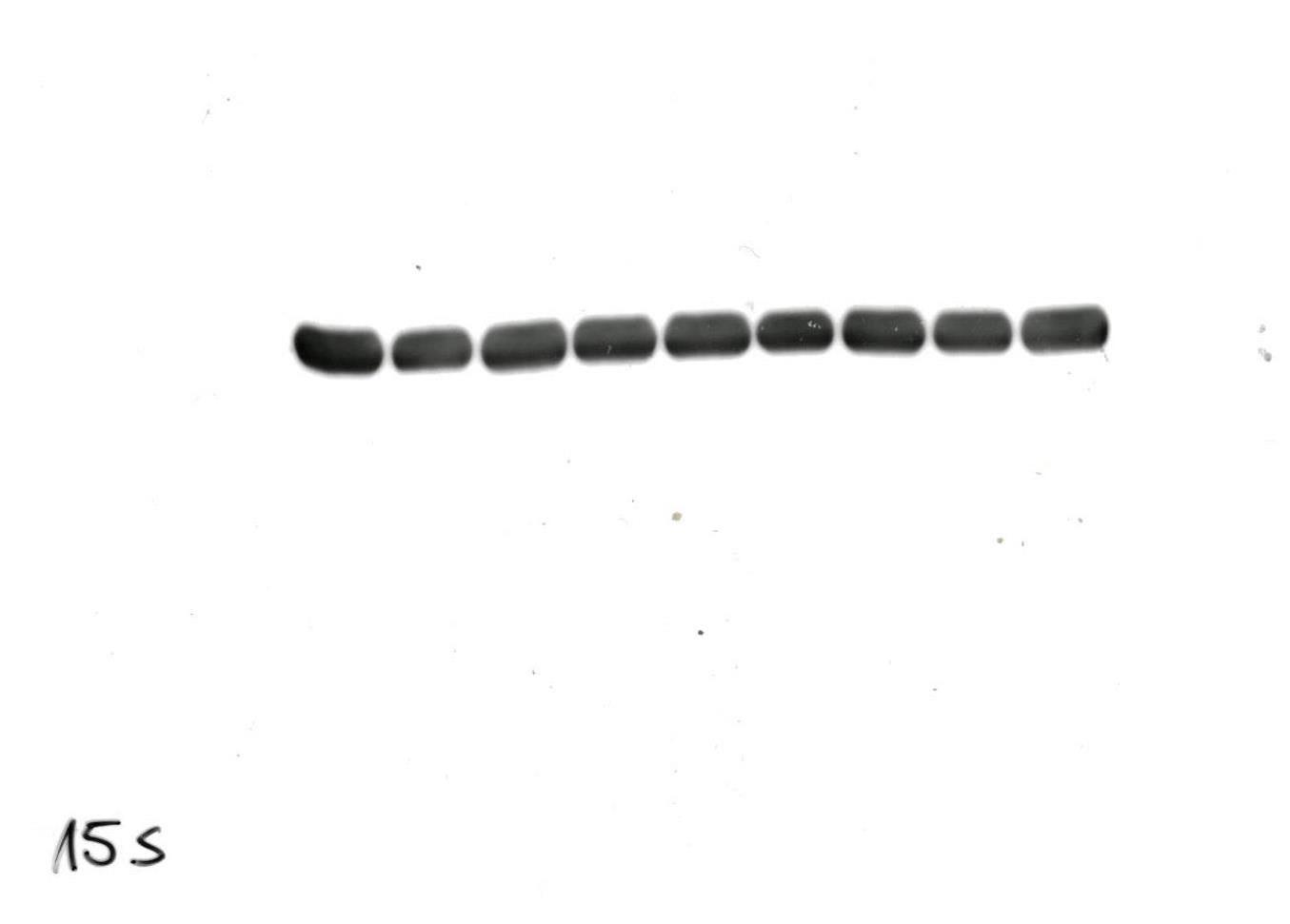

Supplement: S1 File — (ZIP) [file pone.0248926.s001.zip › S1/FigS2_C_Tubulin.jpg]

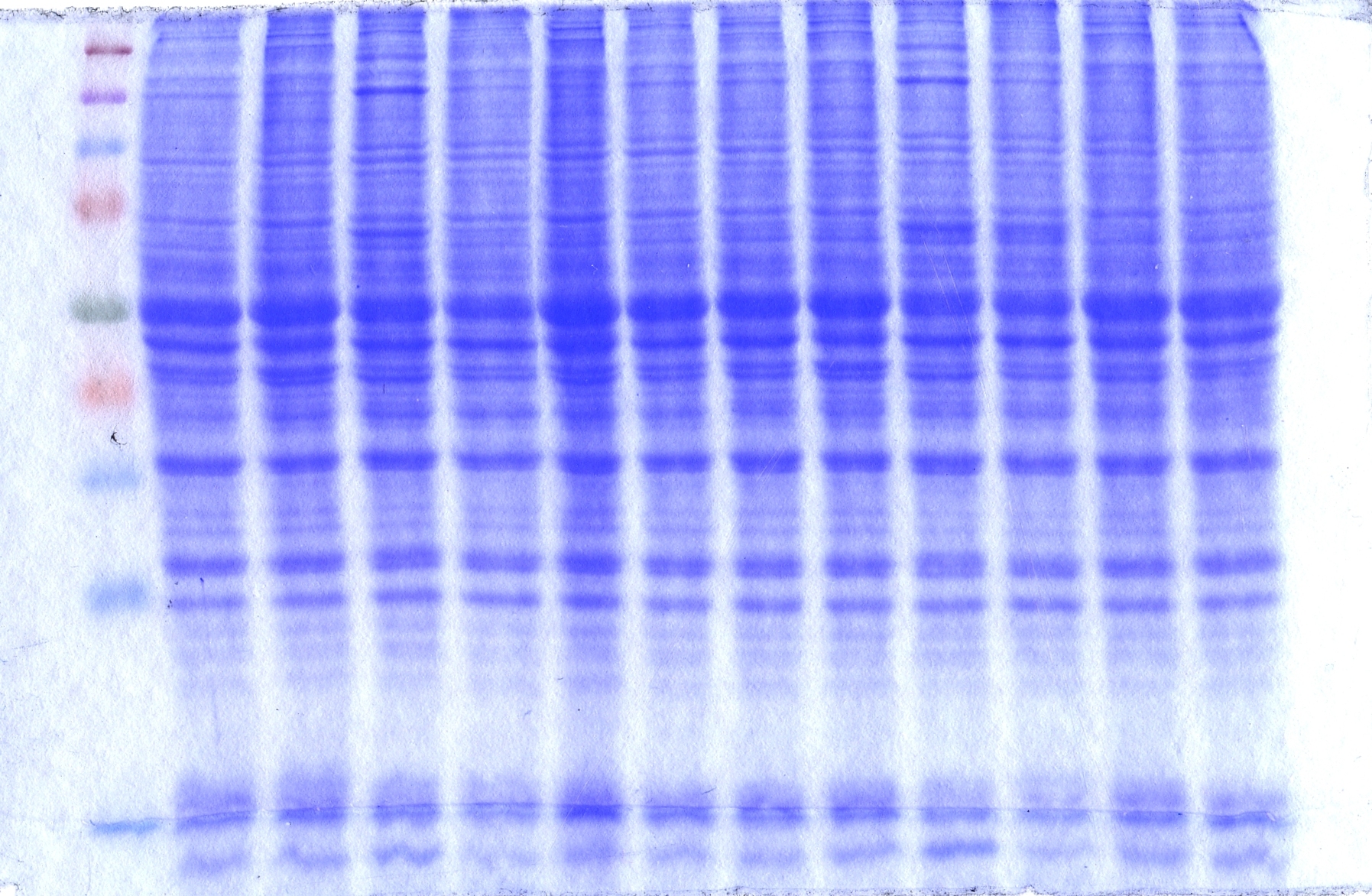

Supplement: S1 File — (ZIP) [file pone.0248926.s001.zip › S1/FigS3_A_Coomasie.jpg]

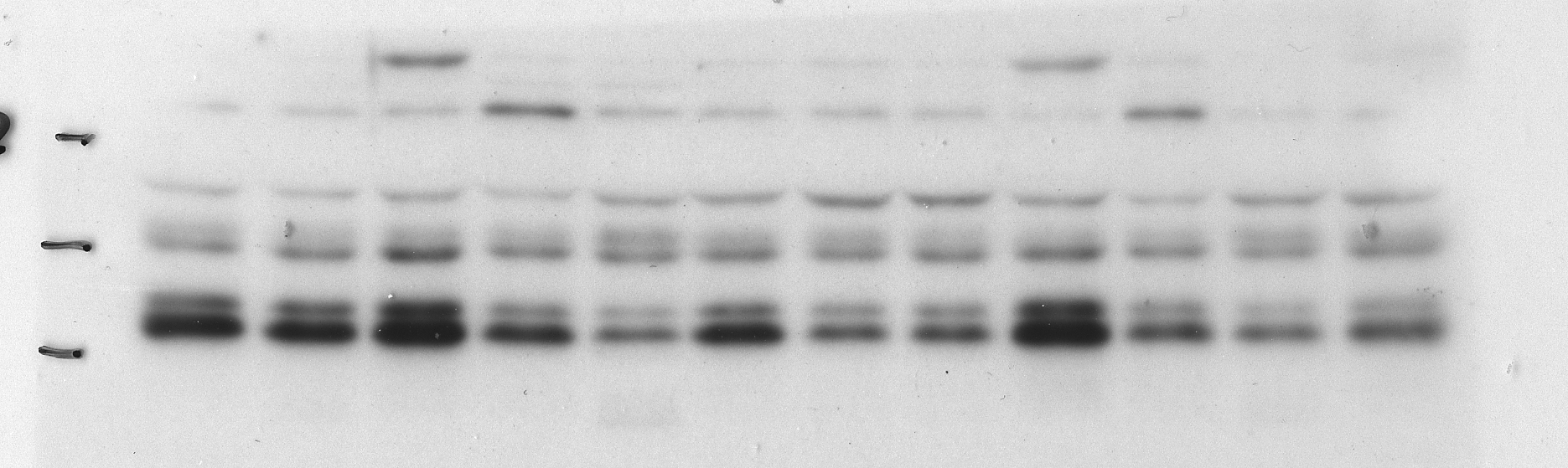

Supplement: S1 File — (ZIP) [file pone.0248926.s001.zip › S1/FigS3_A_SIRT2.jpg]

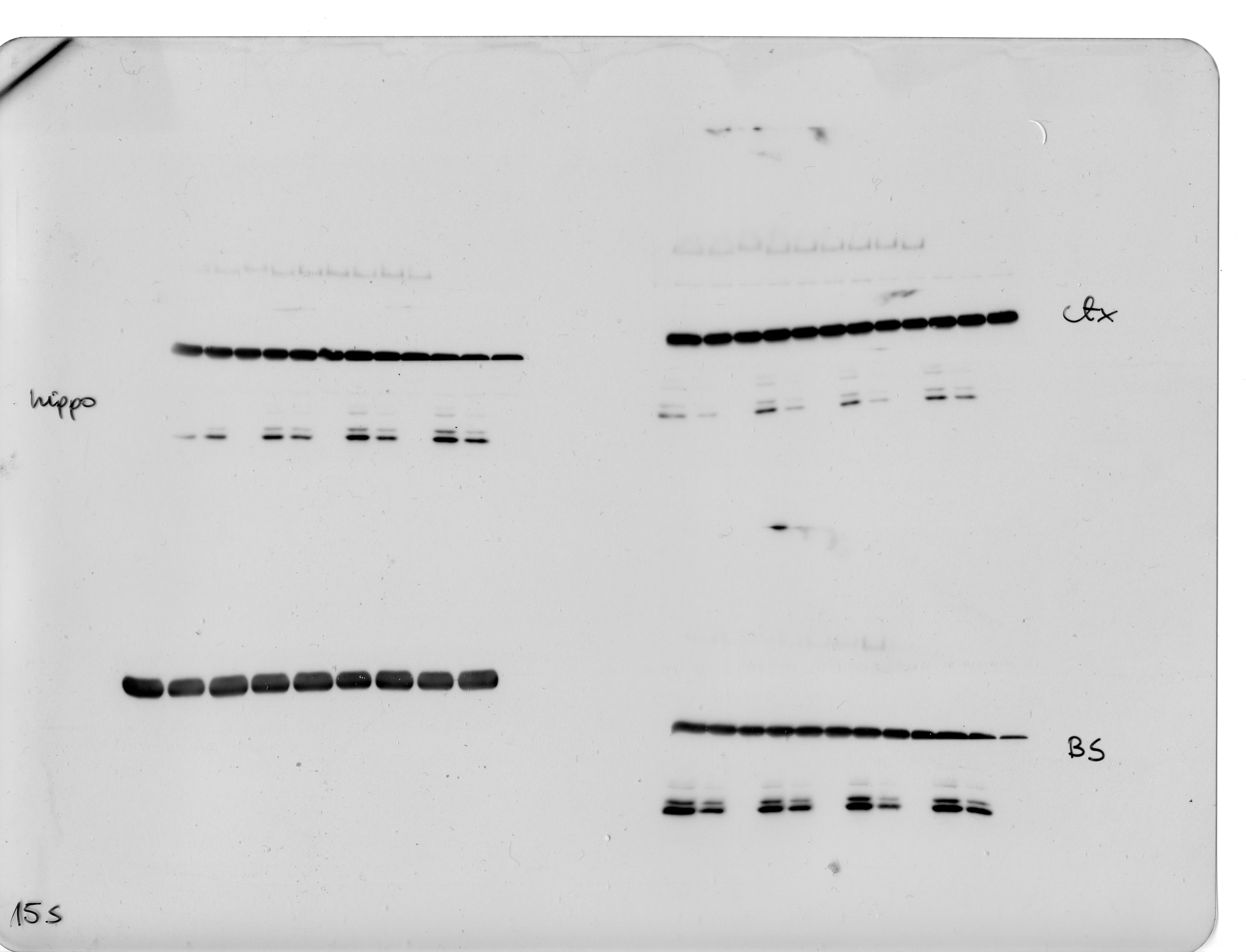

Supplement: S2 File — (ZIP) [file pone.0248926.s002.zip › S2/S2/1 Ctx Hippo BS 15 s - tubulin.jpg]

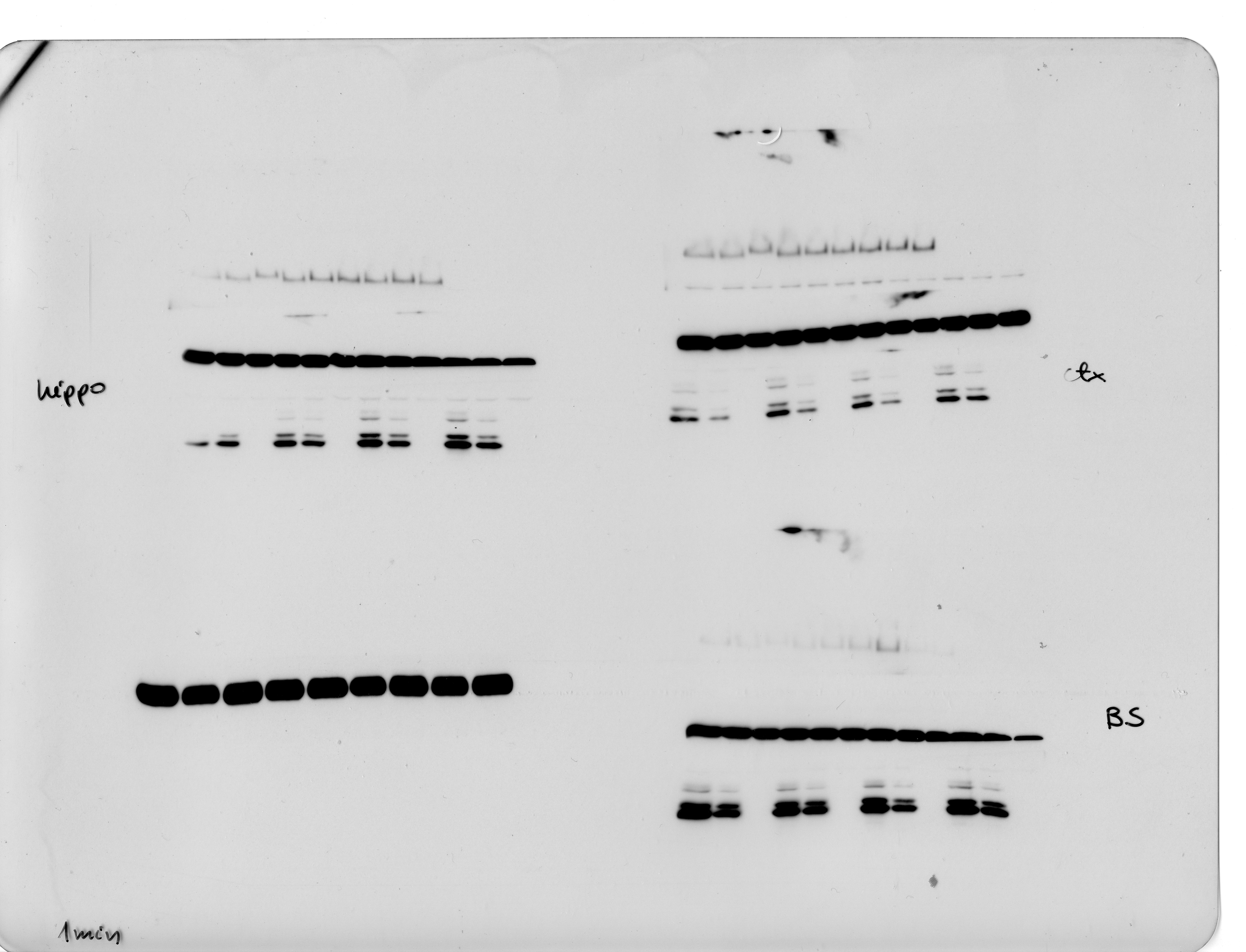

Supplement: S2 File — (ZIP) [file pone.0248926.s002.zip › S2/S2/2 Ctx Hippo BS 1 min - soluble ctx and hippo.jpg]

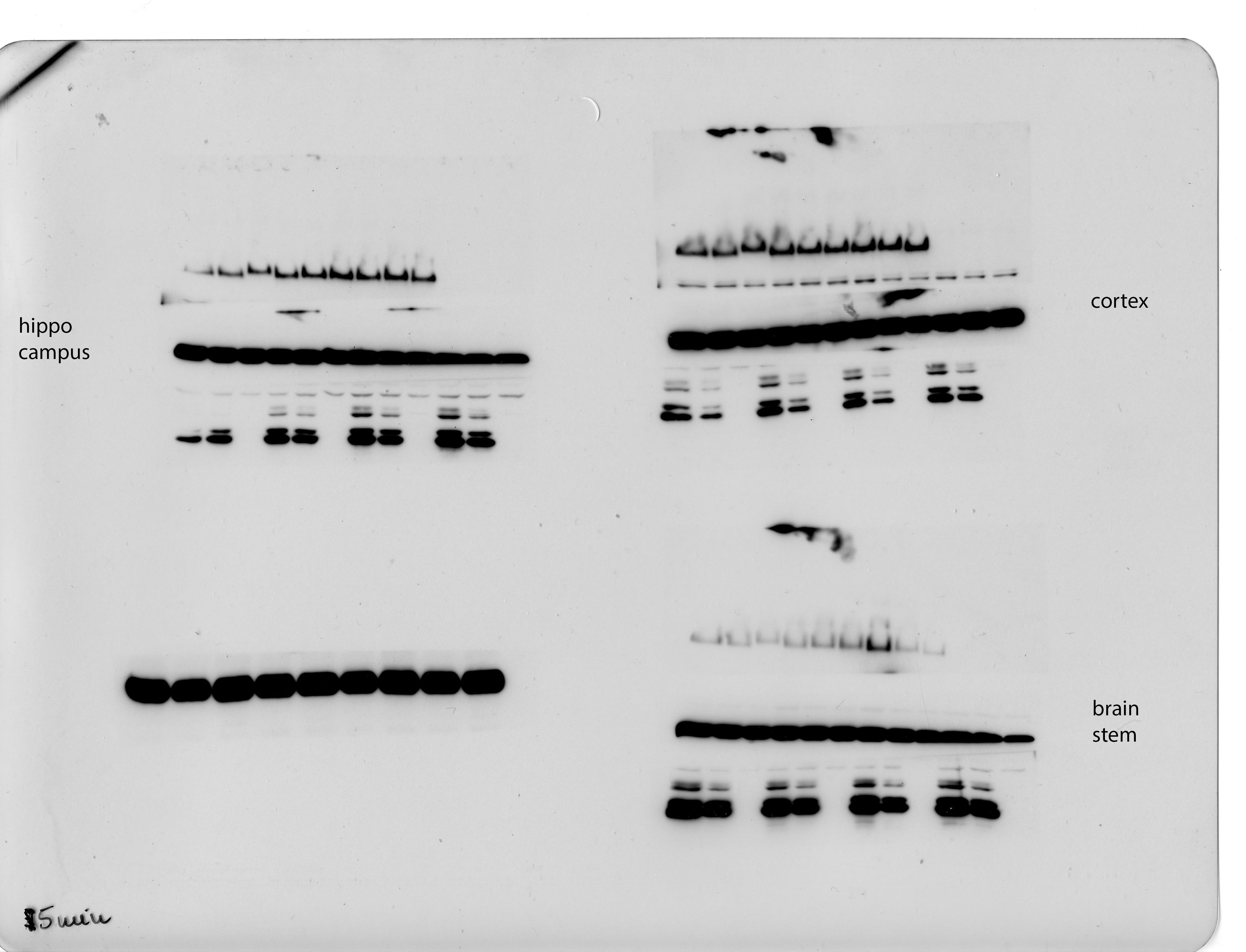

Supplement: S2 File — (ZIP) [file pone.0248926.s002.zip › S2/S2/3 Ctx Hippo BS 5 min.jpg]

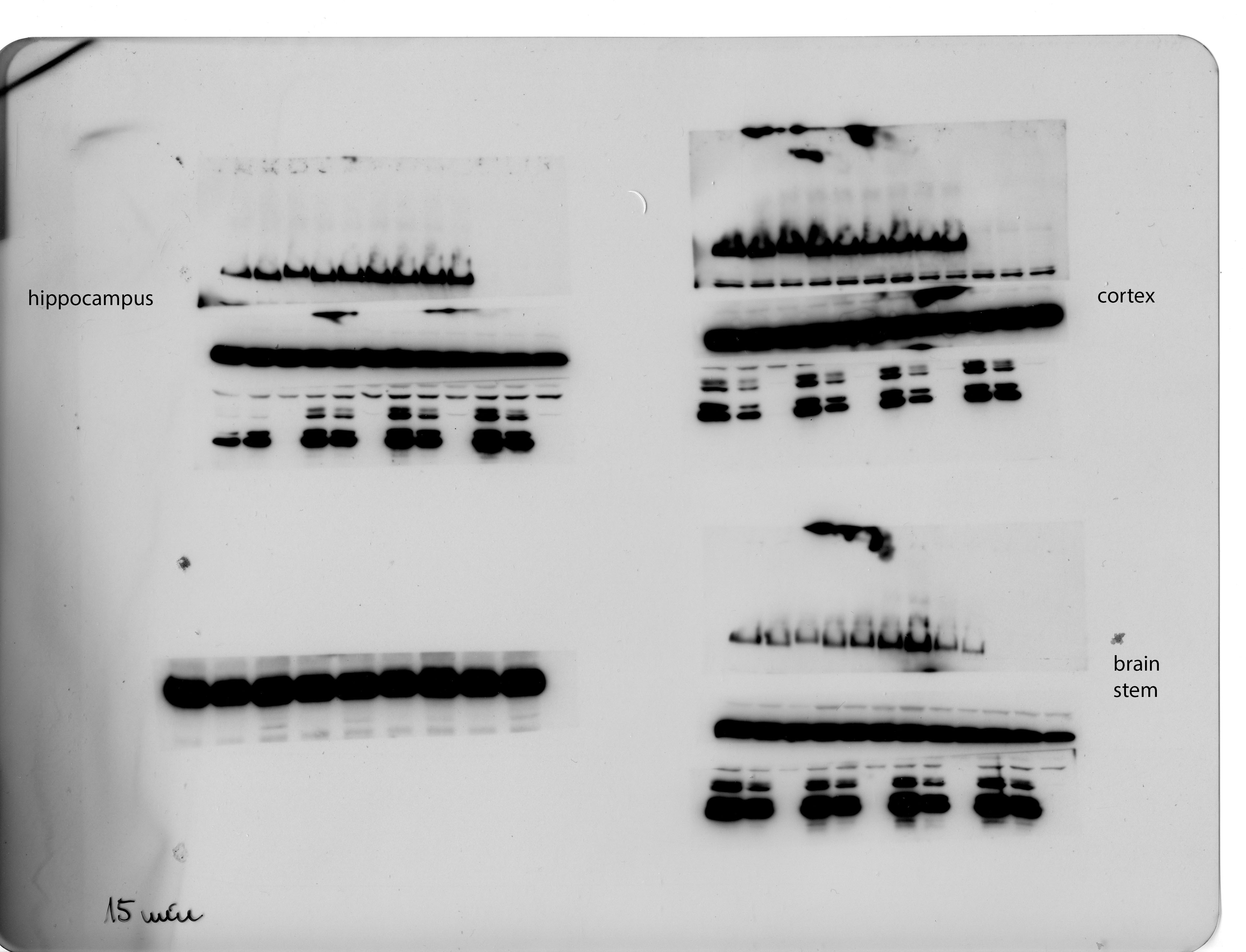

Supplement: S2 File — (ZIP) [file pone.0248926.s002.zip › S2/S2/4 Ctx Hippo BS 15 min.jpg]

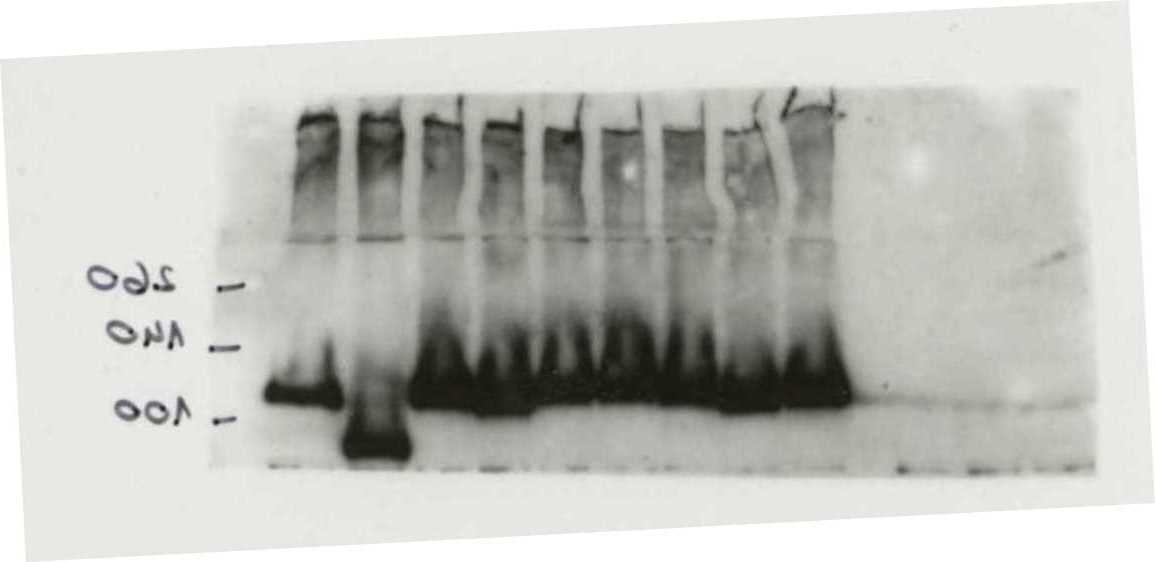

Supplement: S3 File — (ZIP) [file pone.0248926.s003.zip › S3/S3/1_Cortex 9 wks aggregates.jpg]

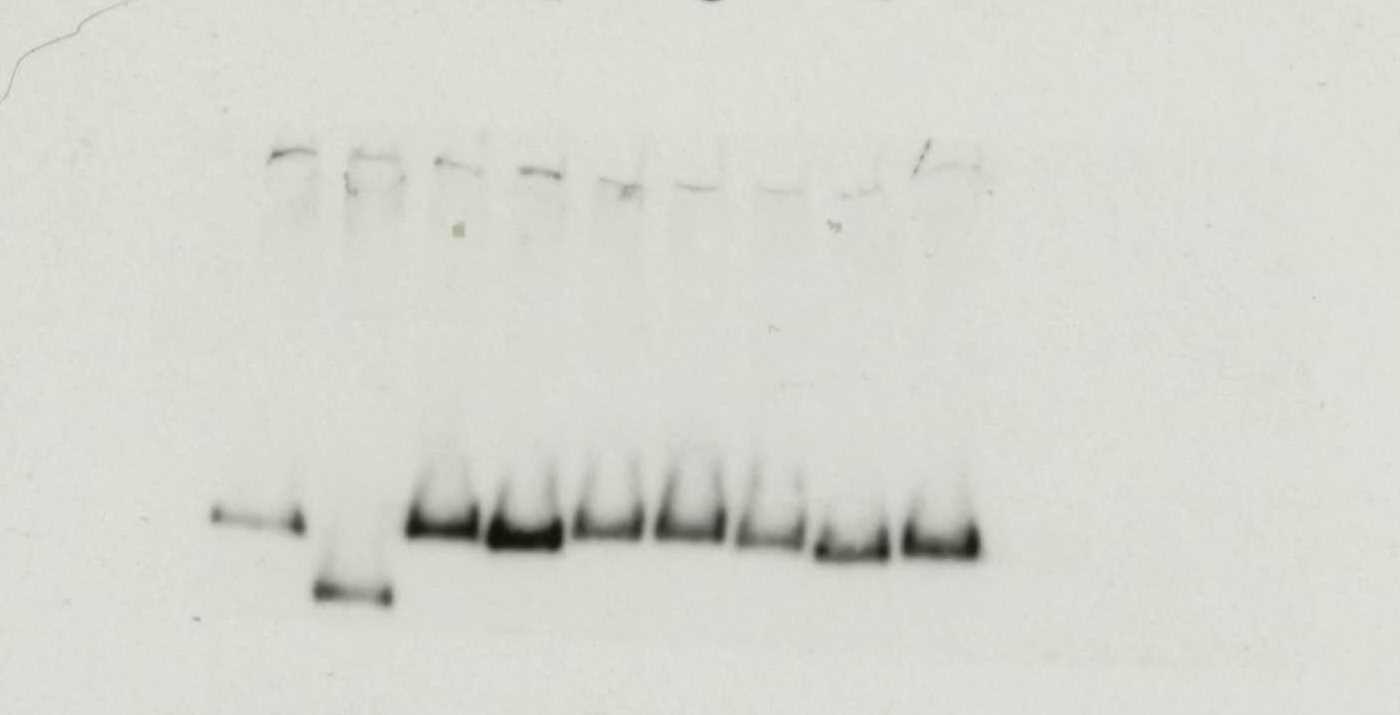

Supplement: S3 File — (ZIP) [file pone.0248926.s003.zip › S3/S3/1_Cortex 9 wks HTT soluble.jpg]

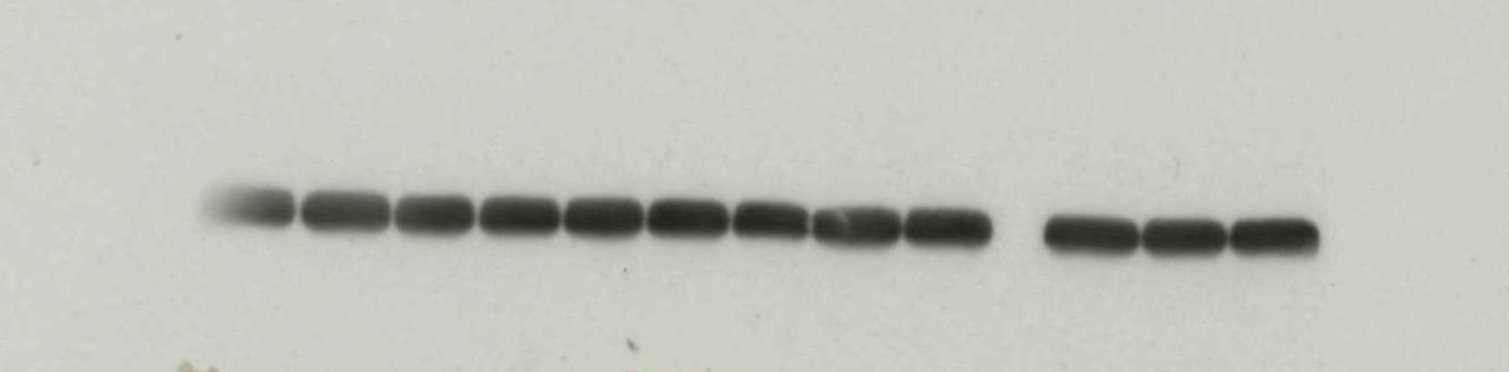

Supplement: S3 File — (ZIP) [file pone.0248926.s003.zip › S3/S3/1_Cortex 9 wks tubulin.jpg]

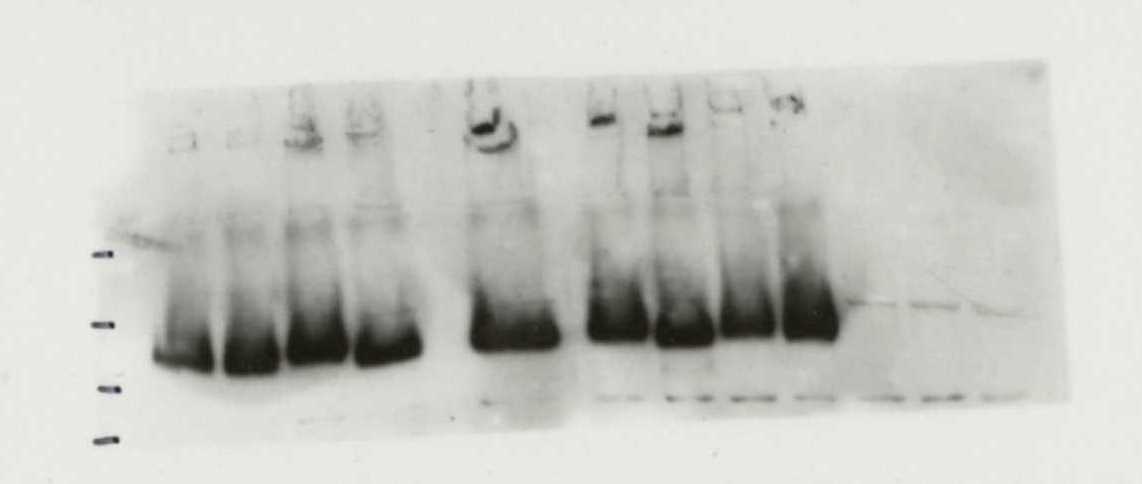

Supplement: S3 File — (ZIP) [file pone.0248926.s003.zip › S3/S3/3_Brain stem 9 wks HTT aggregates match soluble.jpg]

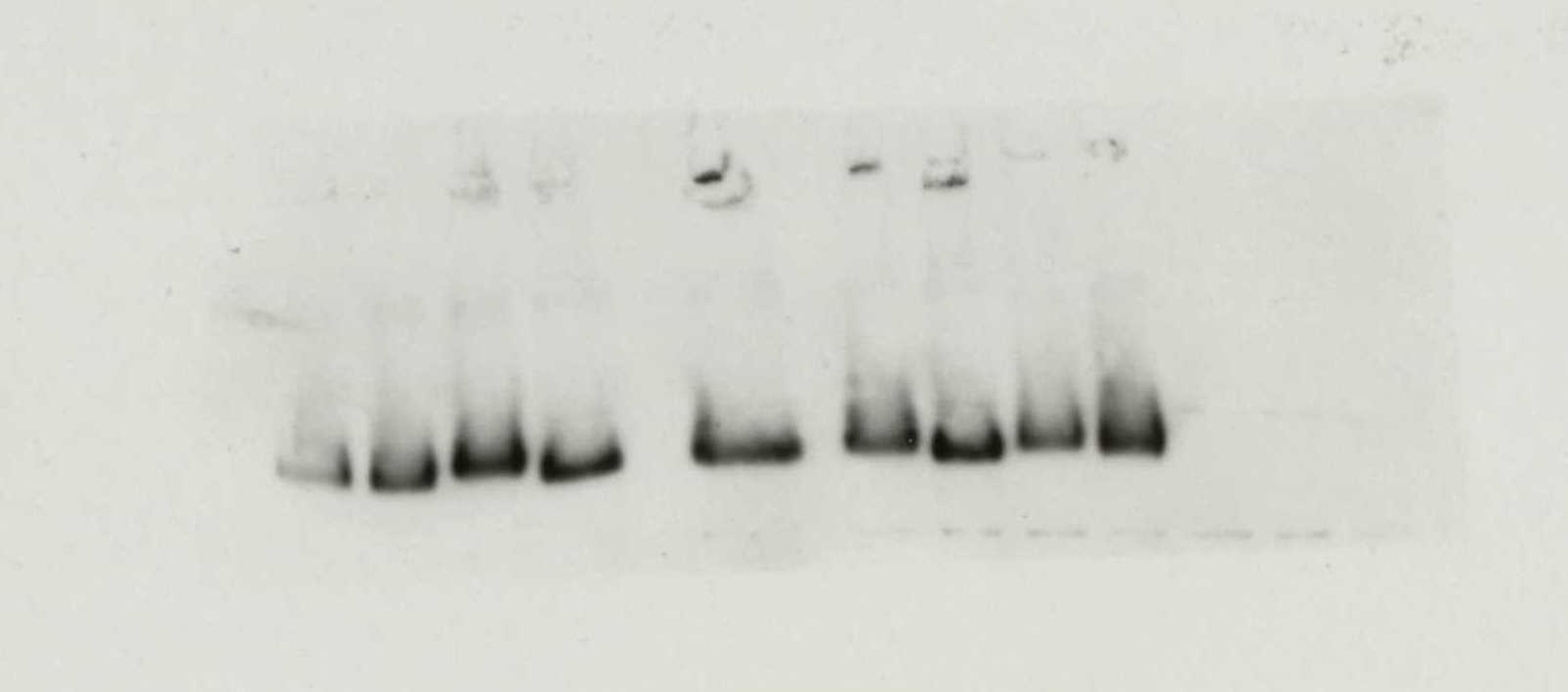

Supplement: S3 File — (ZIP) [file pone.0248926.s003.zip › S3/S3/3_Brain stem 9 wks HTT soluble.jpg]

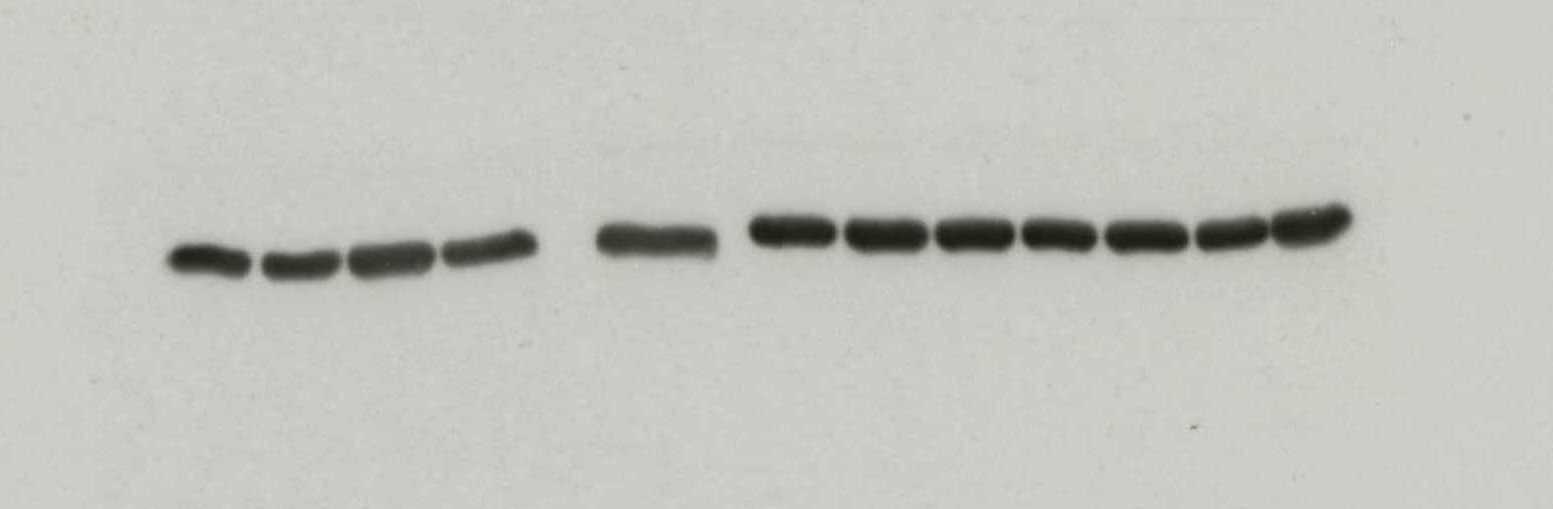

Supplement: S3 File — (ZIP) [file pone.0248926.s003.zip › S3/S3/3_Brain stem 9 wks tubulin soluble.jpg]

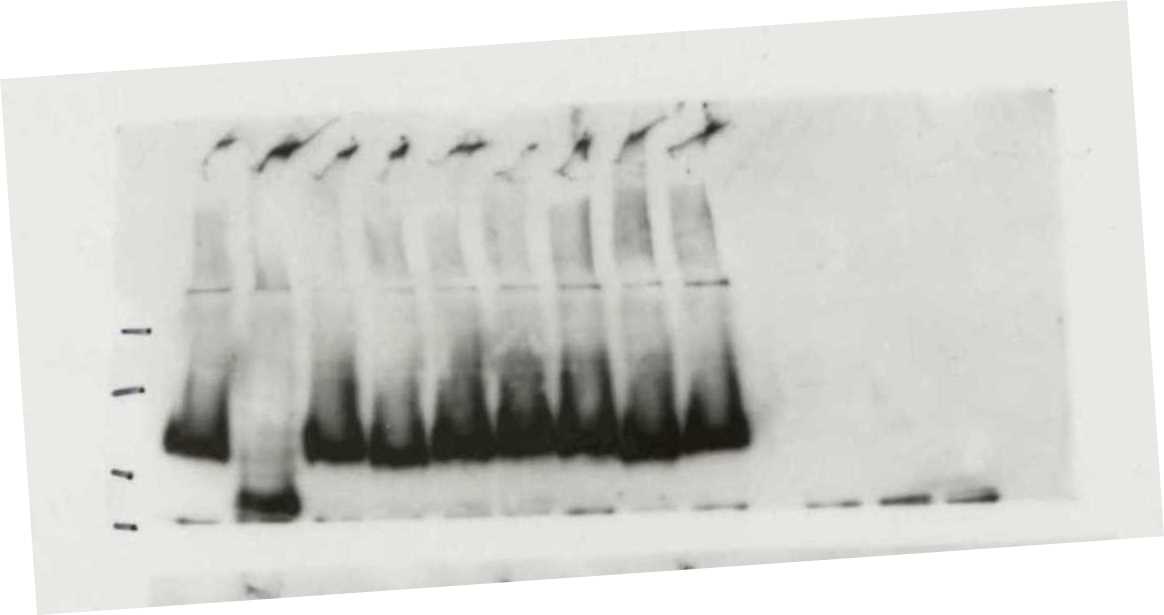

Supplement: S3 File — (ZIP) [file pone.0248926.s003.zip › S3/S3/4_Hippocampus 9 wks HTT aggreagates.jpg]

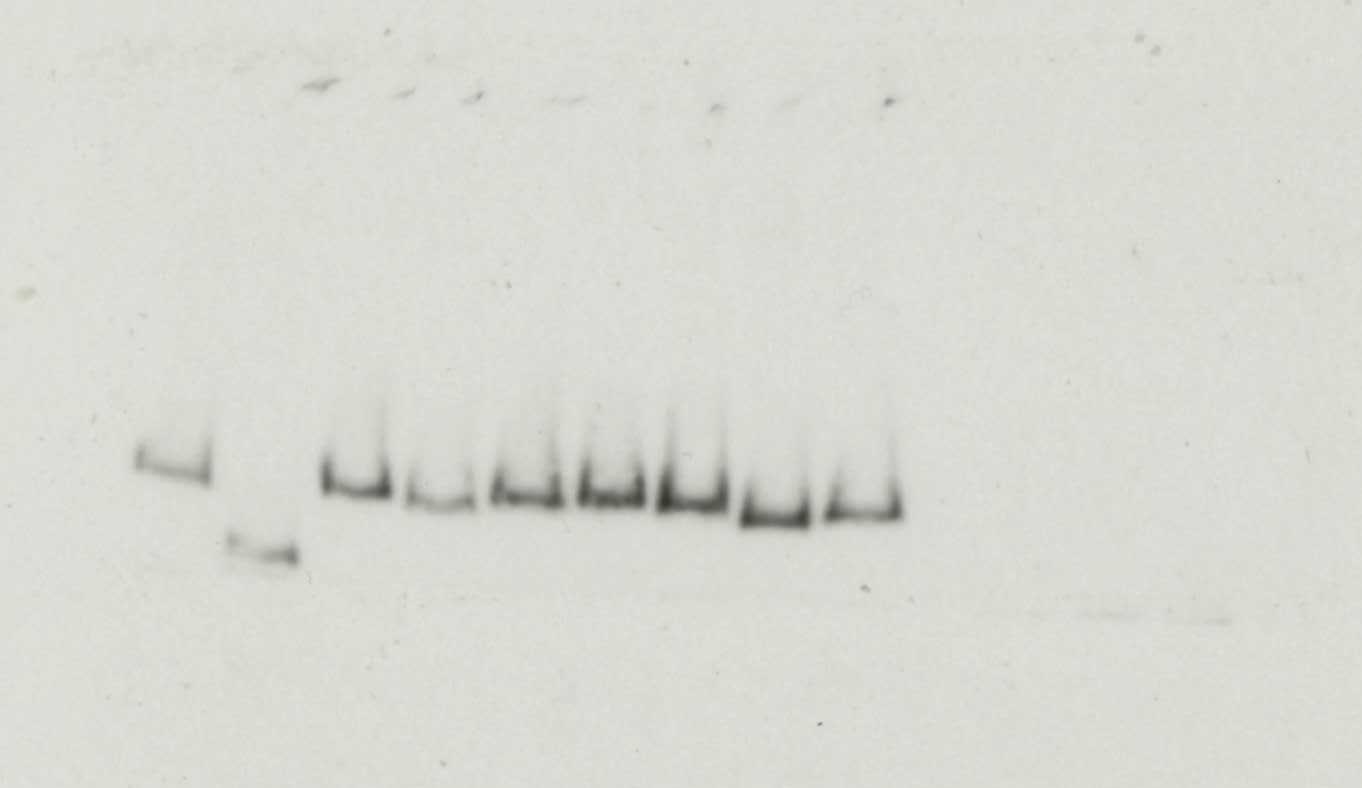

Supplement: S3 File — (ZIP) [file pone.0248926.s003.zip › S3/S3/4_Hippocampus 9 wks HTT soluble.jpg]

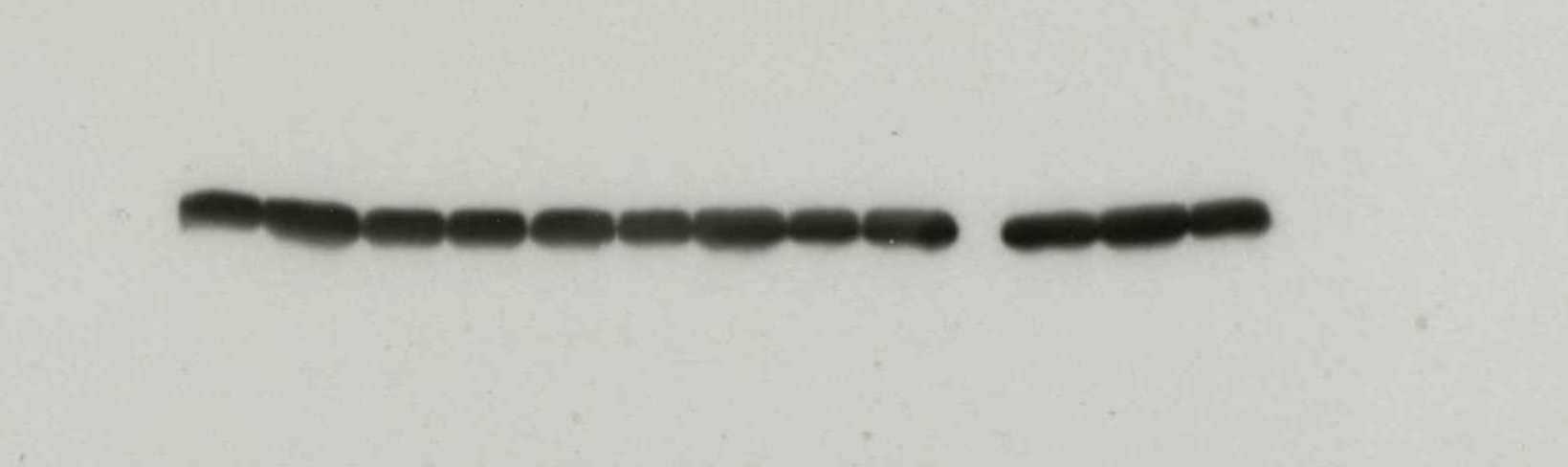

Supplement: S3 File — (ZIP) [file pone.0248926.s003.zip › S3/S3/4_Hippocampus 9 wks tubulin.jpg]

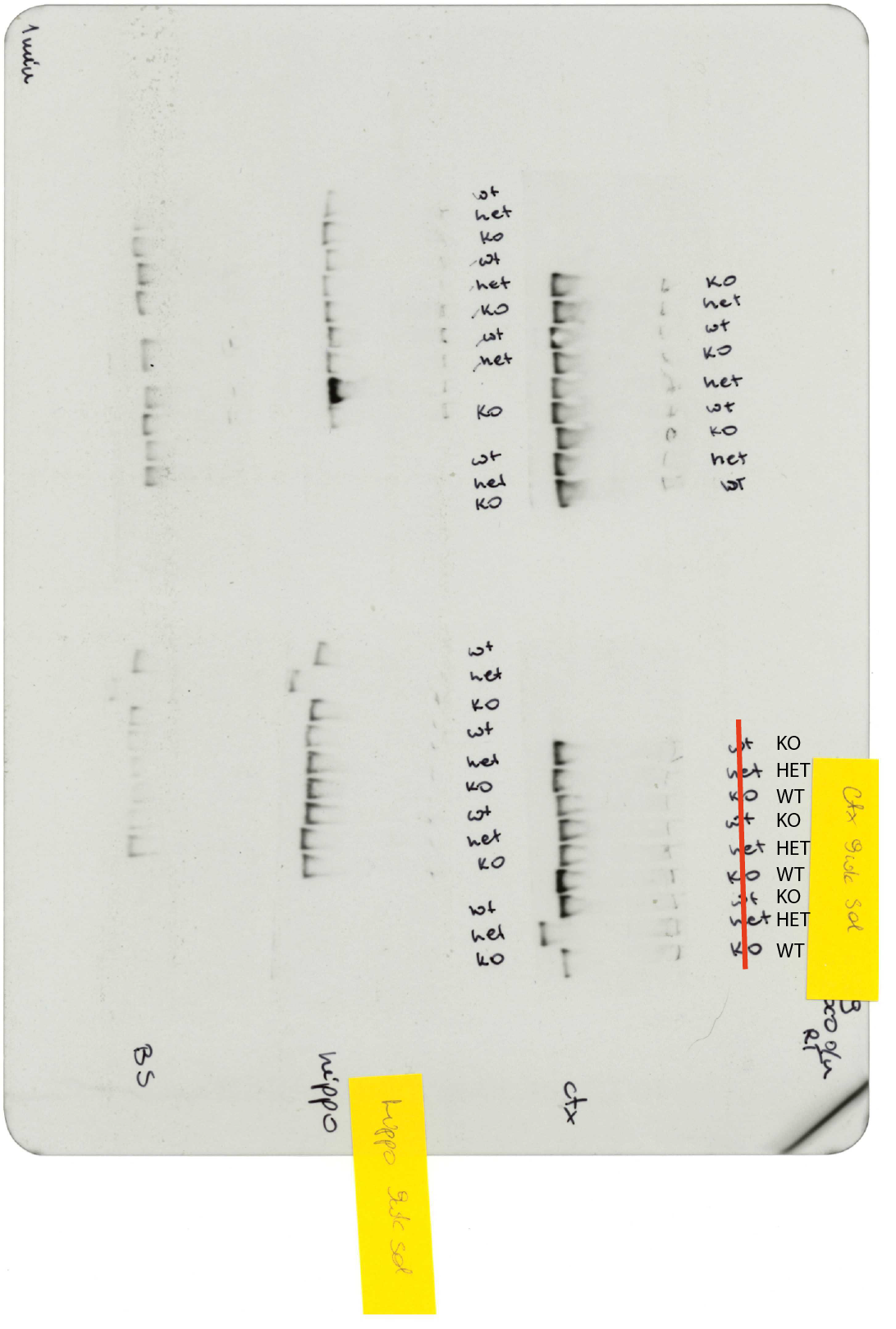

Supplement: S3 File — (ZIP) [file pone.0248926.s003.zip › S3/S3/Fig 5 C-D Whole Film Images/9 weeks HTT 1 min_Correct Labels.png]

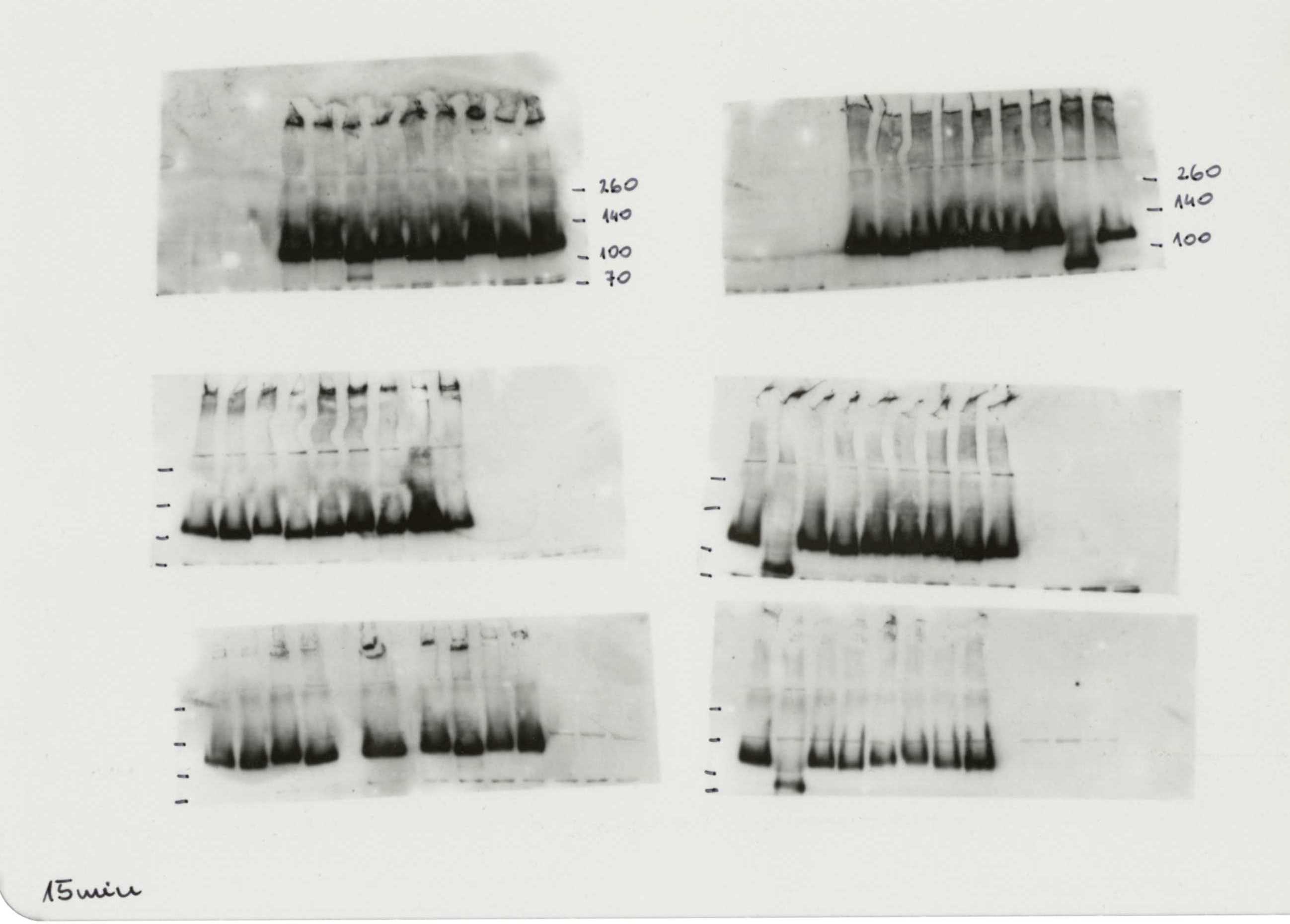

Supplement: S3 File — (ZIP) [file pone.0248926.s003.zip › S3/S3/Fig 5 C-D Whole Film Images/9 weeks HTT 15 min.jpg]

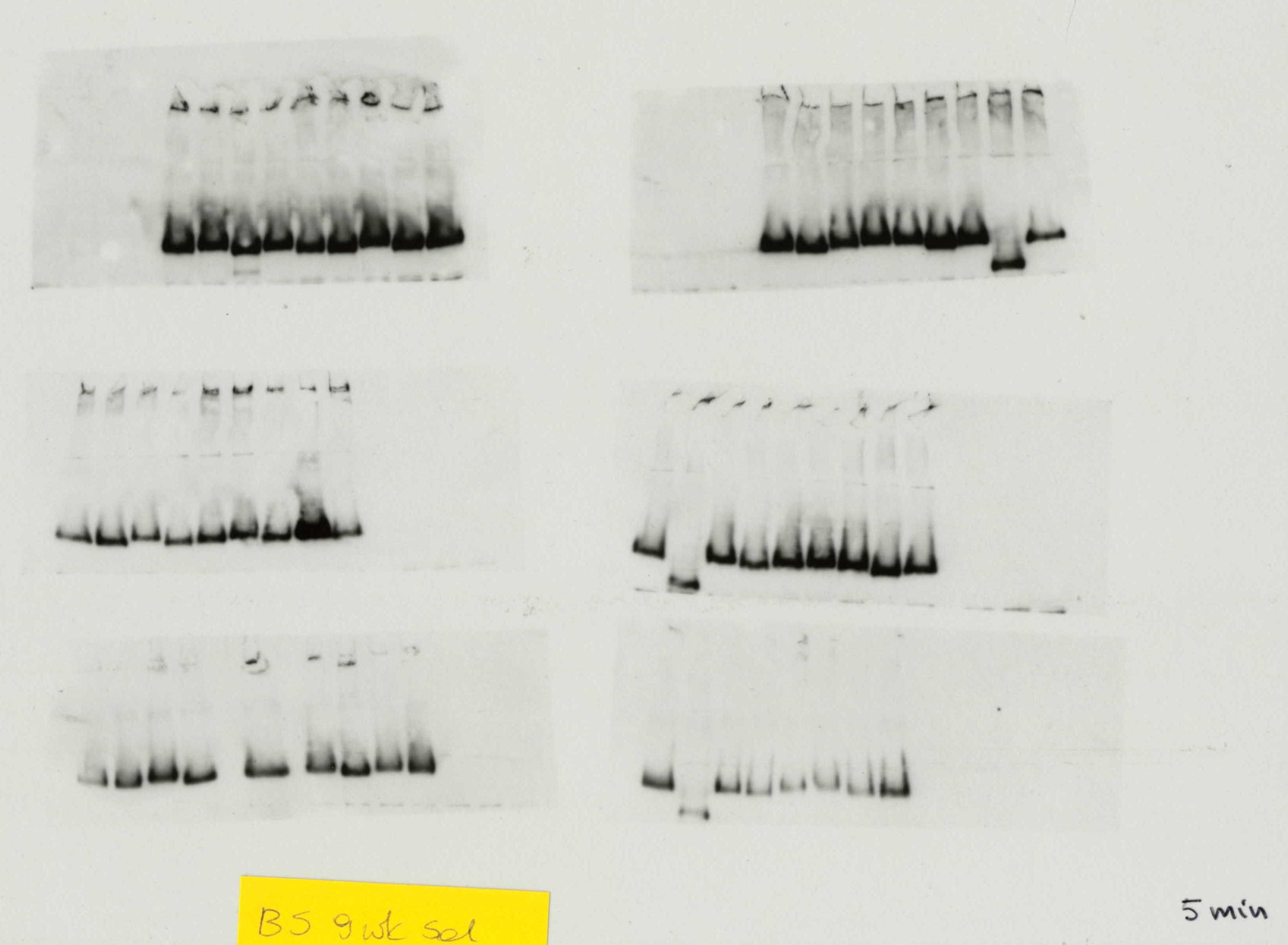

Supplement: S3 File — (ZIP) [file pone.0248926.s003.zip › S3/S3/Fig 5 C-D Whole Film Images/9 weeks HTT 5 min.jpg]

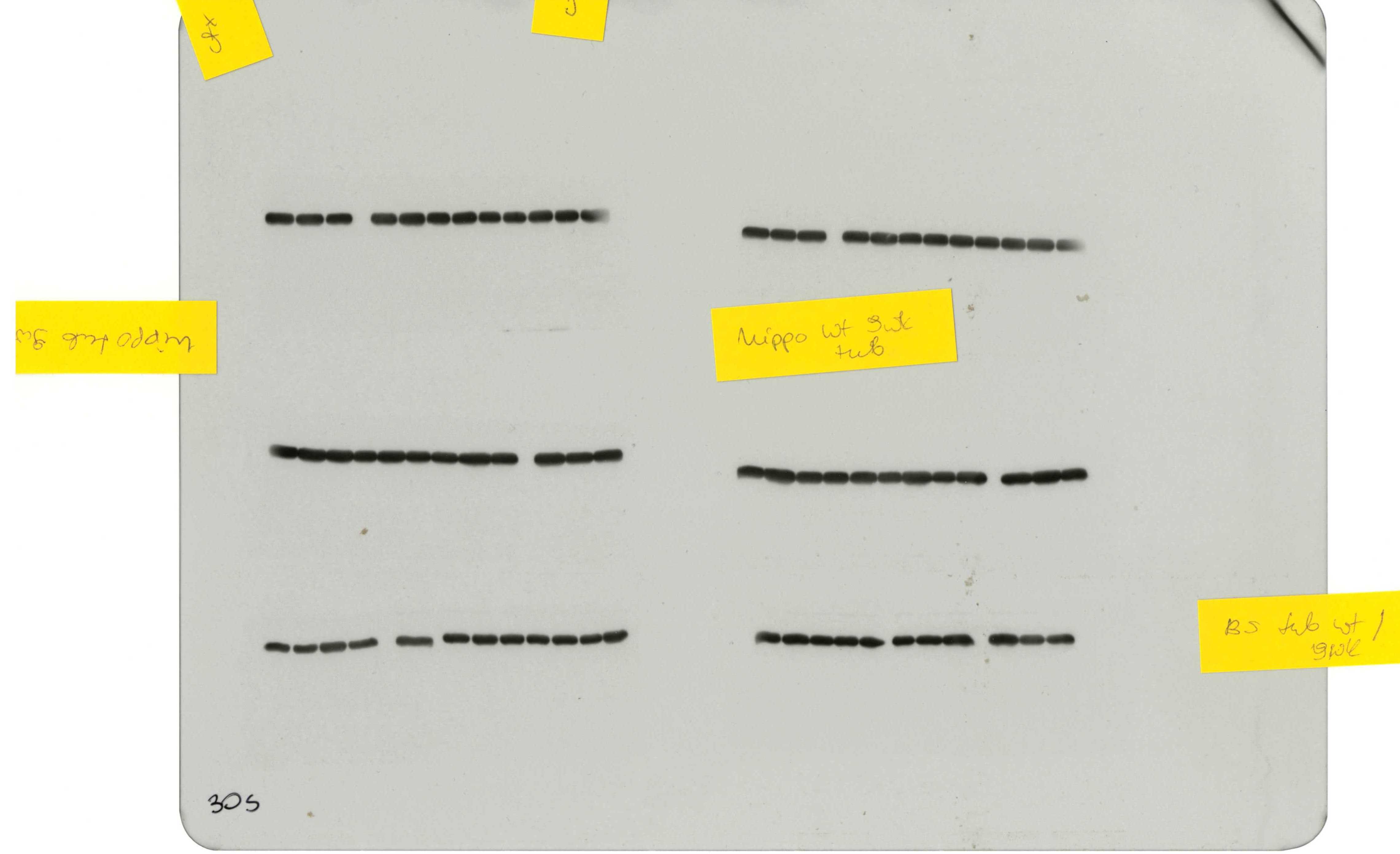

Supplement: S3 File — (ZIP) [file pone.0248926.s003.zip › S3/S3/Fig 5 C-D Whole Film Images/9 weeks tub 30 sec.jpg]

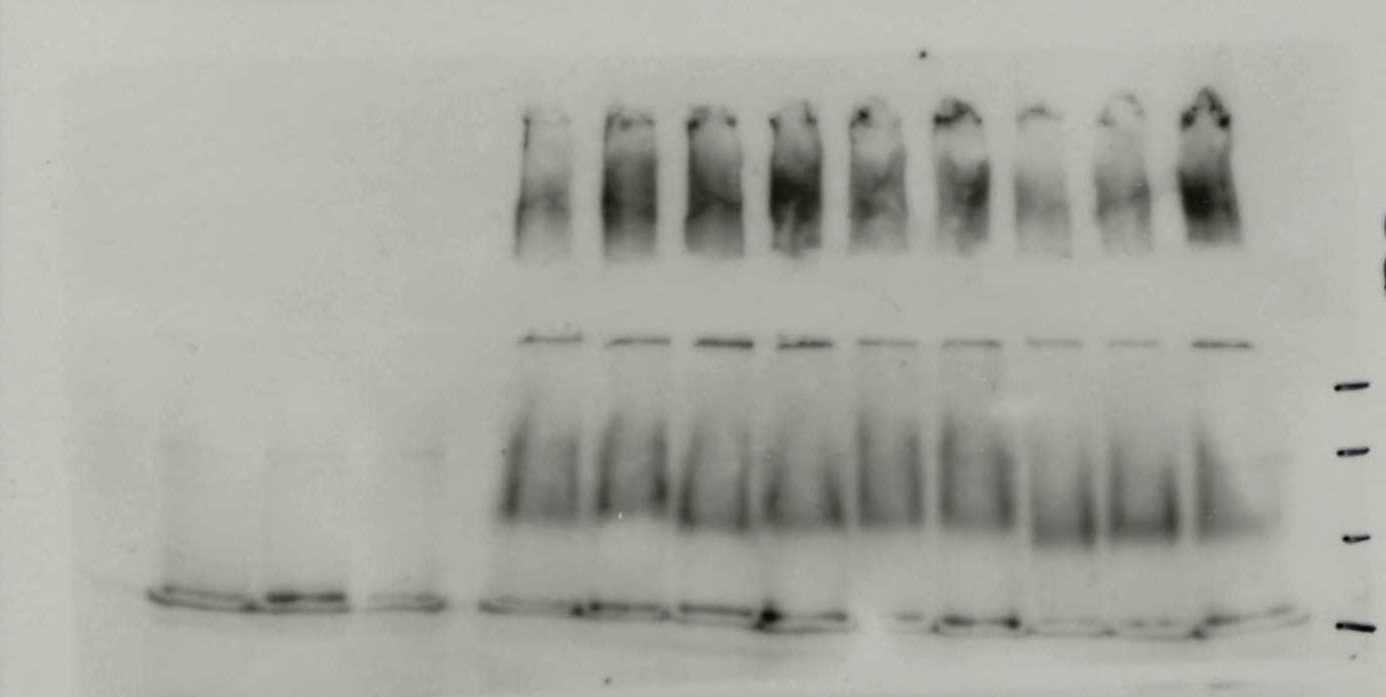

Supplement: S4 File — (ZIP) [file pone.0248926.s004.zip › S4/S4/12_Brain stem 15 wks HTT.jpg]

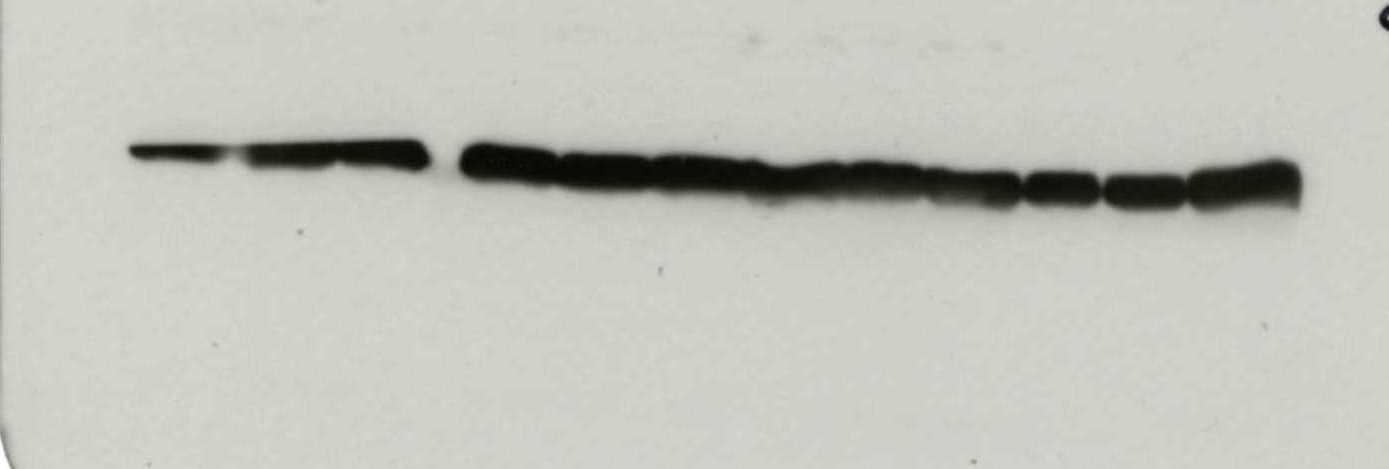

Supplement: S4 File — (ZIP) [file pone.0248926.s004.zip › S4/S4/12_Brain stem 15 wks tubulin.jpg]

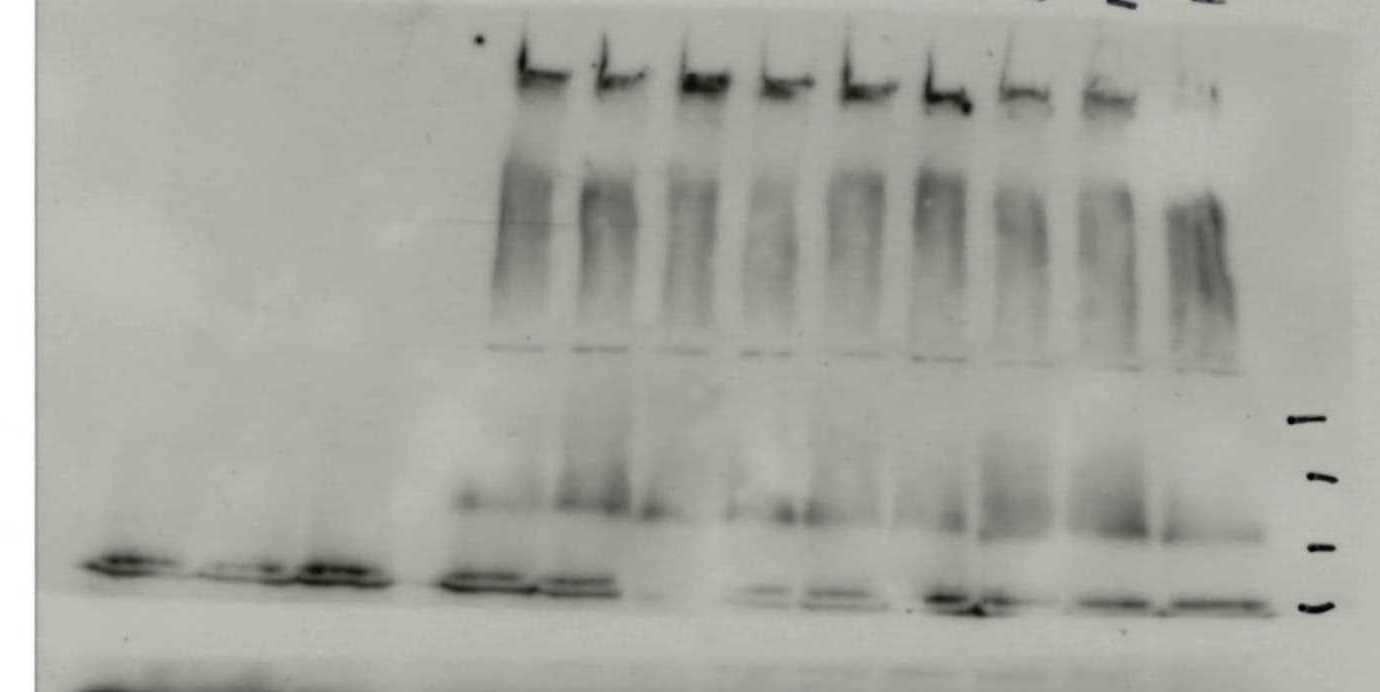

Supplement: S4 File — (ZIP) [file pone.0248926.s004.zip › S4/S4/6_Cortex 15 wks HTT.jpg]

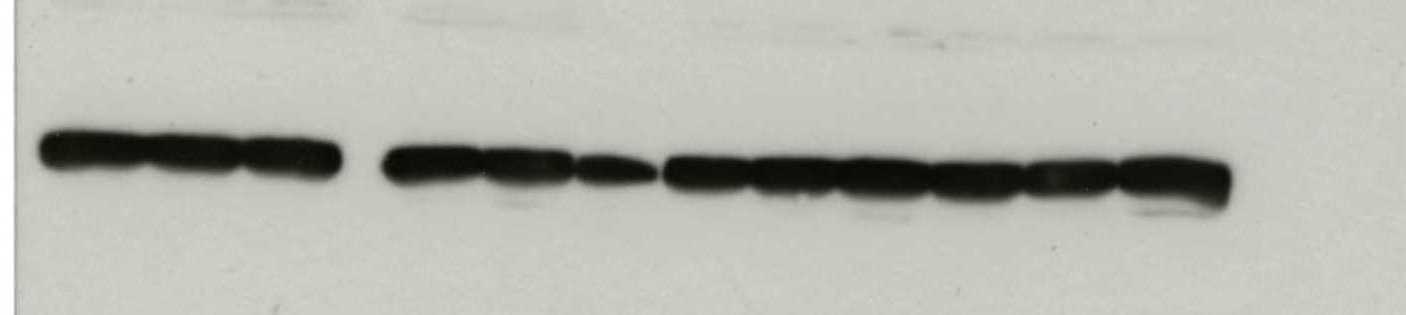

Supplement: S4 File — (ZIP) [file pone.0248926.s004.zip › S4/S4/6_Cortex 15 wks tubulin.jpg]

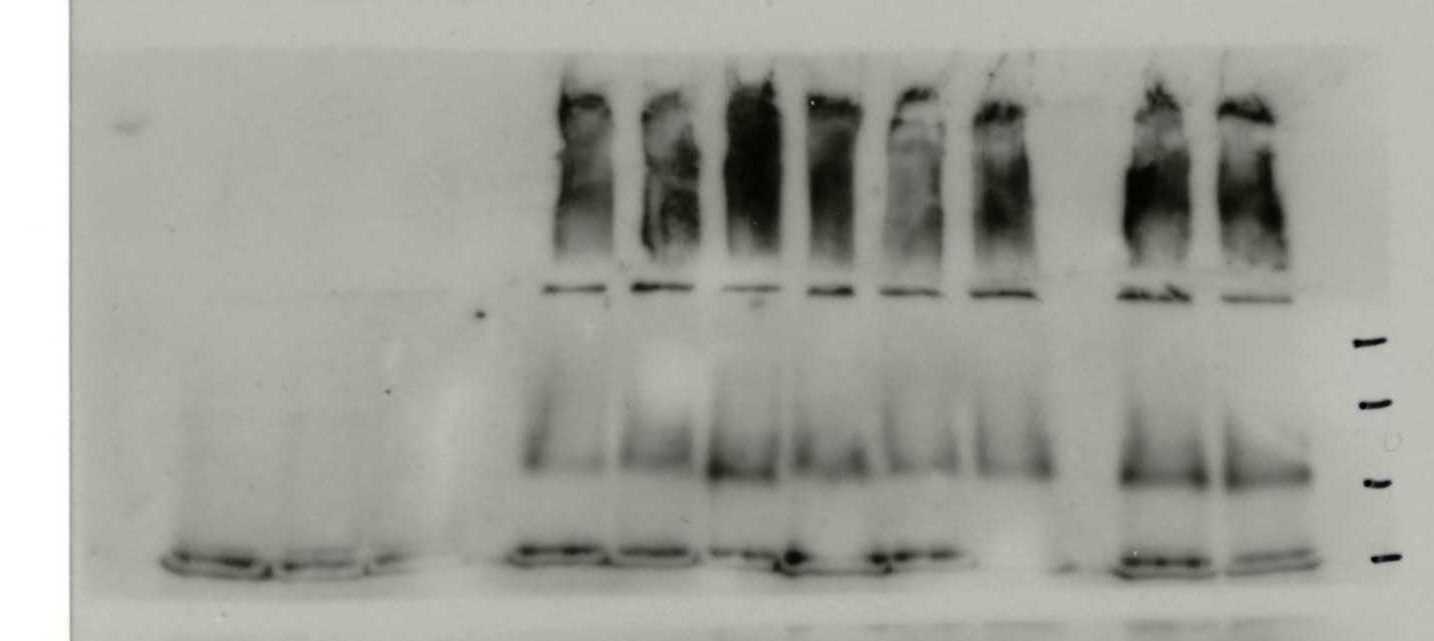

Supplement: S4 File — (ZIP) [file pone.0248926.s004.zip › S4/S4/9_Hippocampus 15 wks HTT.jpg]

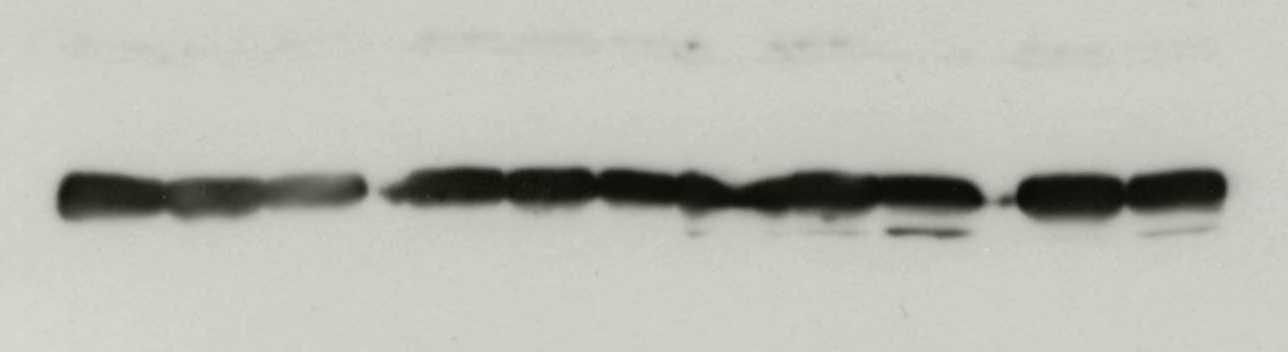

Supplement: S4 File — (ZIP) [file pone.0248926.s004.zip › S4/S4/9_Hippocampus 15 wks tubulin.jpg]

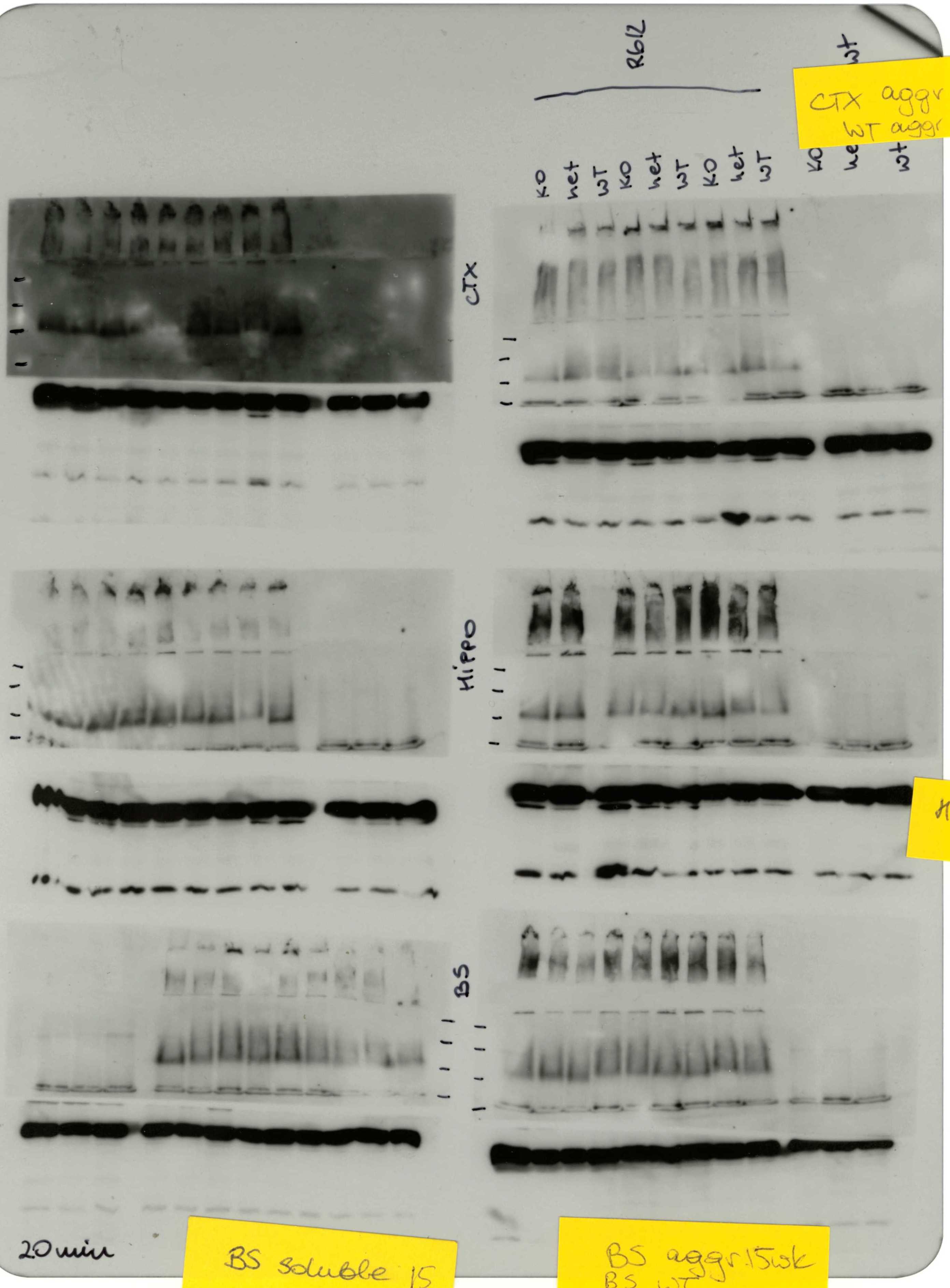

Supplement: S4 File — (ZIP) [file pone.0248926.s004.zip › S4/S4/Fig 5 E-F Whole Film Images/15 weeks 20 min.jpg]

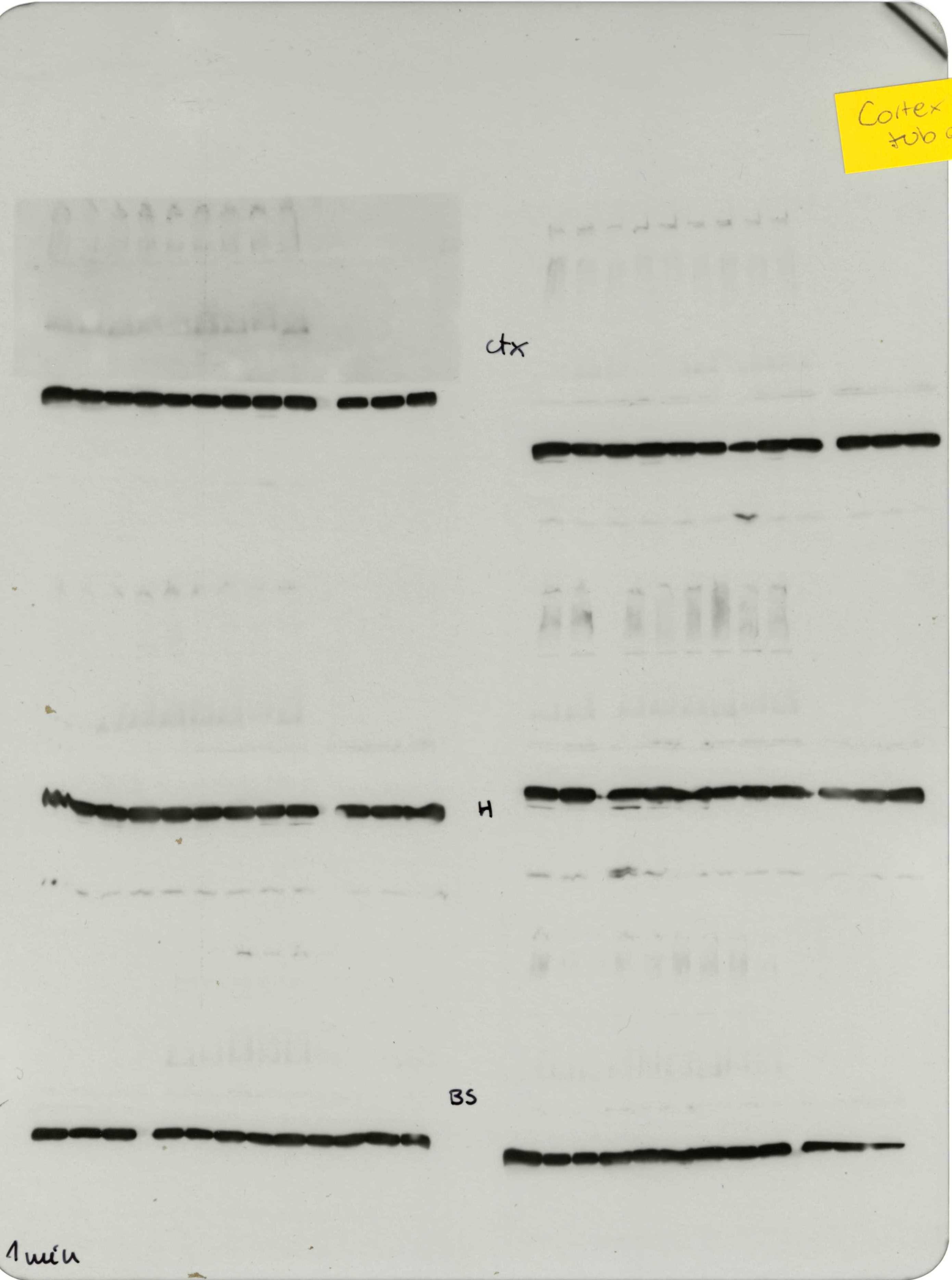

Supplement: S4 File — (ZIP) [file pone.0248926.s004.zip › S4/S4/Fig 5 E-F Whole Film Images/15 weks 1 min.jpg]
