# Supplementary material for: Correction: SIRT2 Ablation Has No Effect on Tubulin Acetylation in Brain, Cholesterol Biosynthesis or the Progression of Huntington’s Disease Phenotypes In Vivo
Source: PLoS One. 2021 Mar 25;16(3):e0248926. doi: 10.1371/journal.pone.0248926 (PMC7993610; doi:10.1371/journal.pone.0248926)
Supplement: S5 File — (PPTX) [file pone.0248926.s005.pptx]

## Slide 1
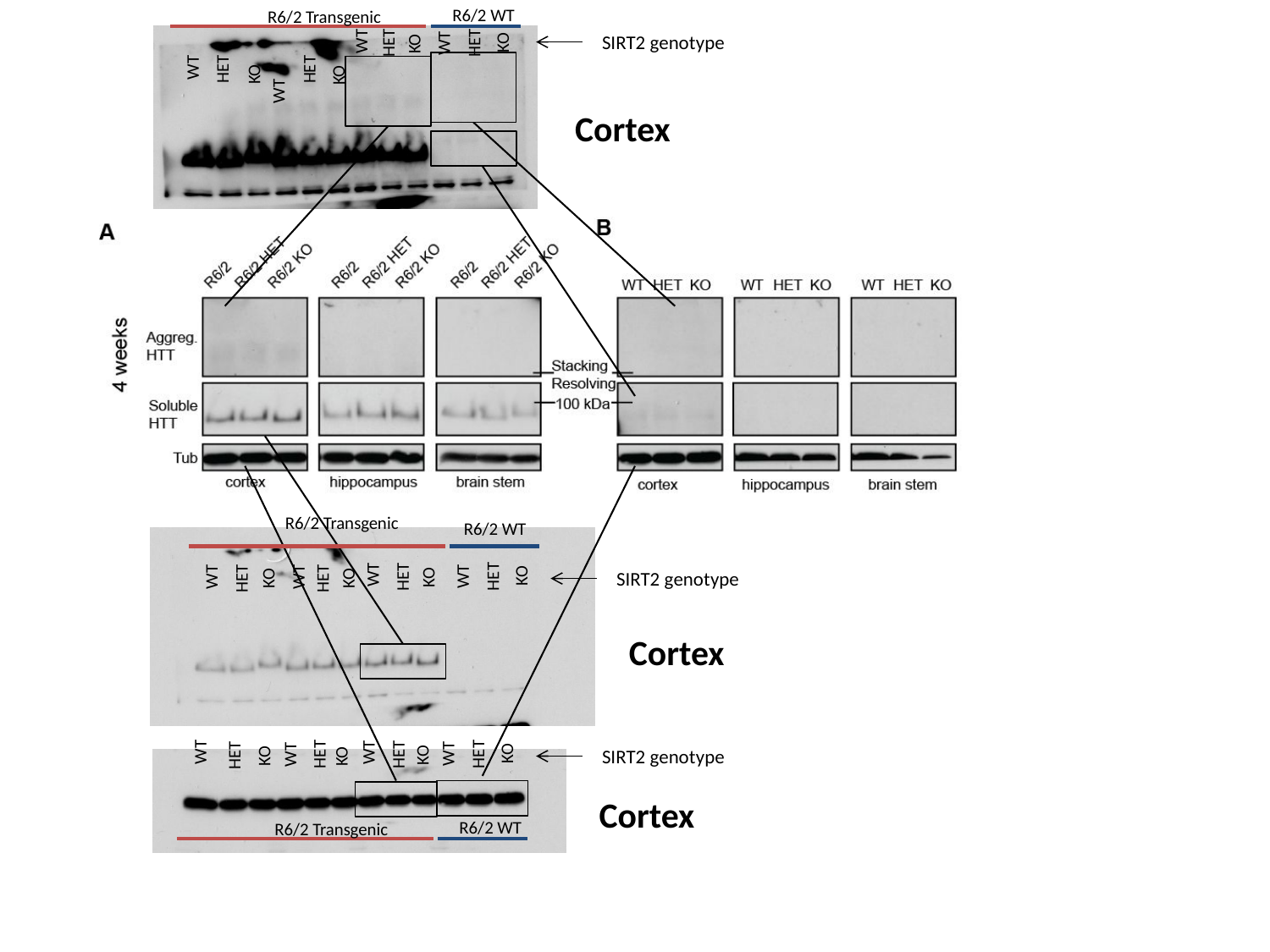

R6/2 WT
R6/2 Transgenic
WT
SIRT2 genotype
KO
WT
HET
HET
KO
WT
HET
HET
KO
KO
WT
Cortex
R6/2 Transgenic
R6/2 WT
WT
KO
WT
HET
HET
WT
WT
KO
SIRT2 genotype
KO
KO
HET
HET
Cortex
WT
WT
KO
WT
HET
HET
HET
HET
WT
KO
SIRT2 genotype
KO
KO
Cortex
R6/2 WT
R6/2 Transgenic

## Slide 2
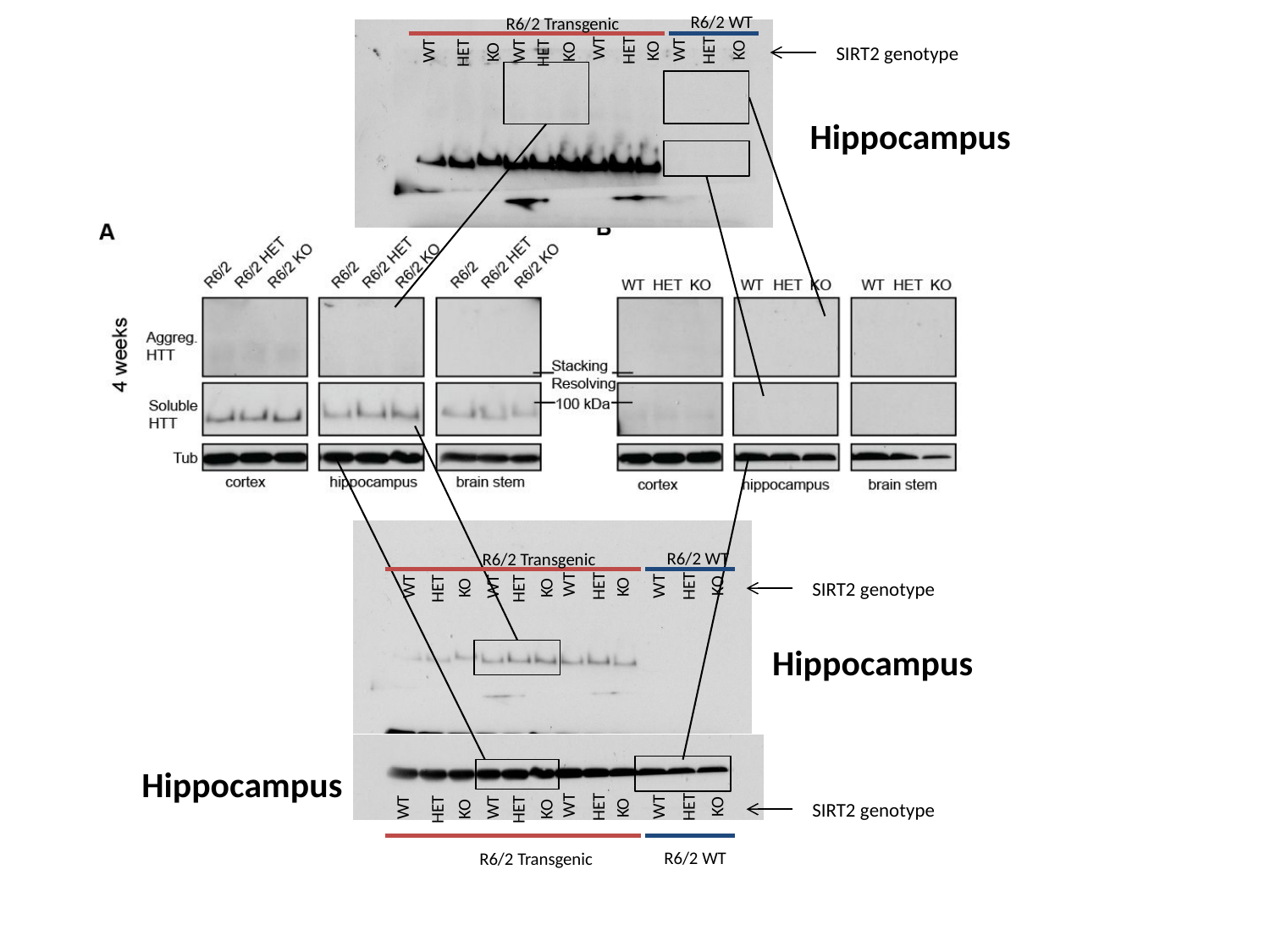

R6/2 WT
R6/2 Transgenic
WT
KO
WT
HET
HET
WT
WT
KO
SIRT2 genotype
KO
KO
HET
HET
Hippocampus
R6/2 WT
R6/2 Transgenic
WT
KO
WT
HET
HET
WT
WT
KO
SIRT2 genotype
KO
KO
HET
HET
Hippocampus
Hippocampus
WT
KO
WT
HET
HET
WT
WT
KO
SIRT2 genotype
KO
KO
HET
HET
R6/2 WT
R6/2 Transgenic

## Slide 3
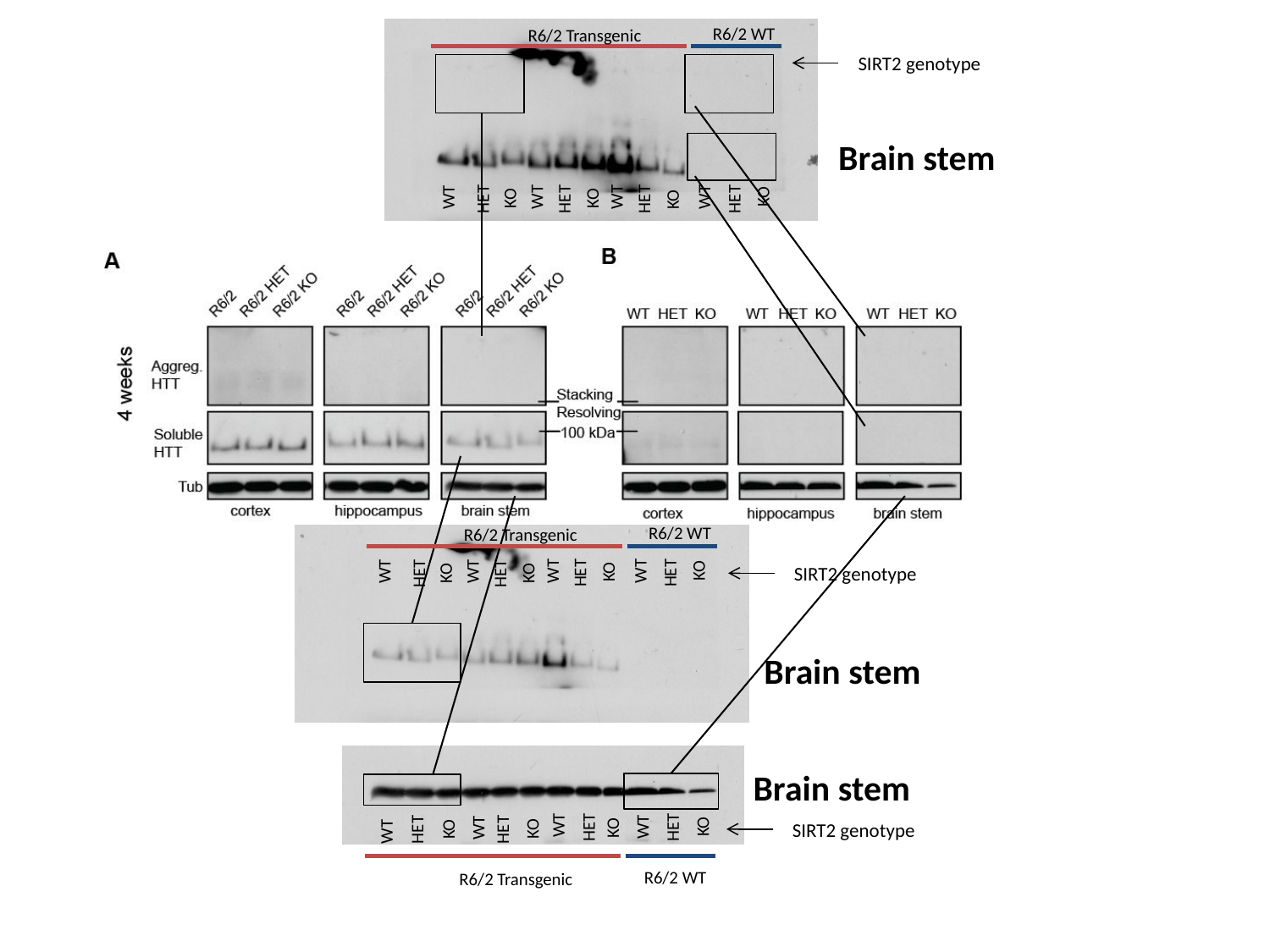

R6/2 WT
R6/2 Transgenic
SIRT2 genotype
Brain stem
KO
WT
WT
WT
WT
KO
KO
HET
HET
HET
HET
KO
R6/2 WT
R6/2 Transgenic
KO
WT
WT
WT
WT
KO
SIRT2 genotype
HET
HET
KO
KO
HET
HET
Brain stem
Brain stem
WT
KO
WT
HET
HET
WT
KO
SIRT2 genotype
KO
KO
HET
HET
WT
R6/2 WT
R6/2 Transgenic

## Slide 4
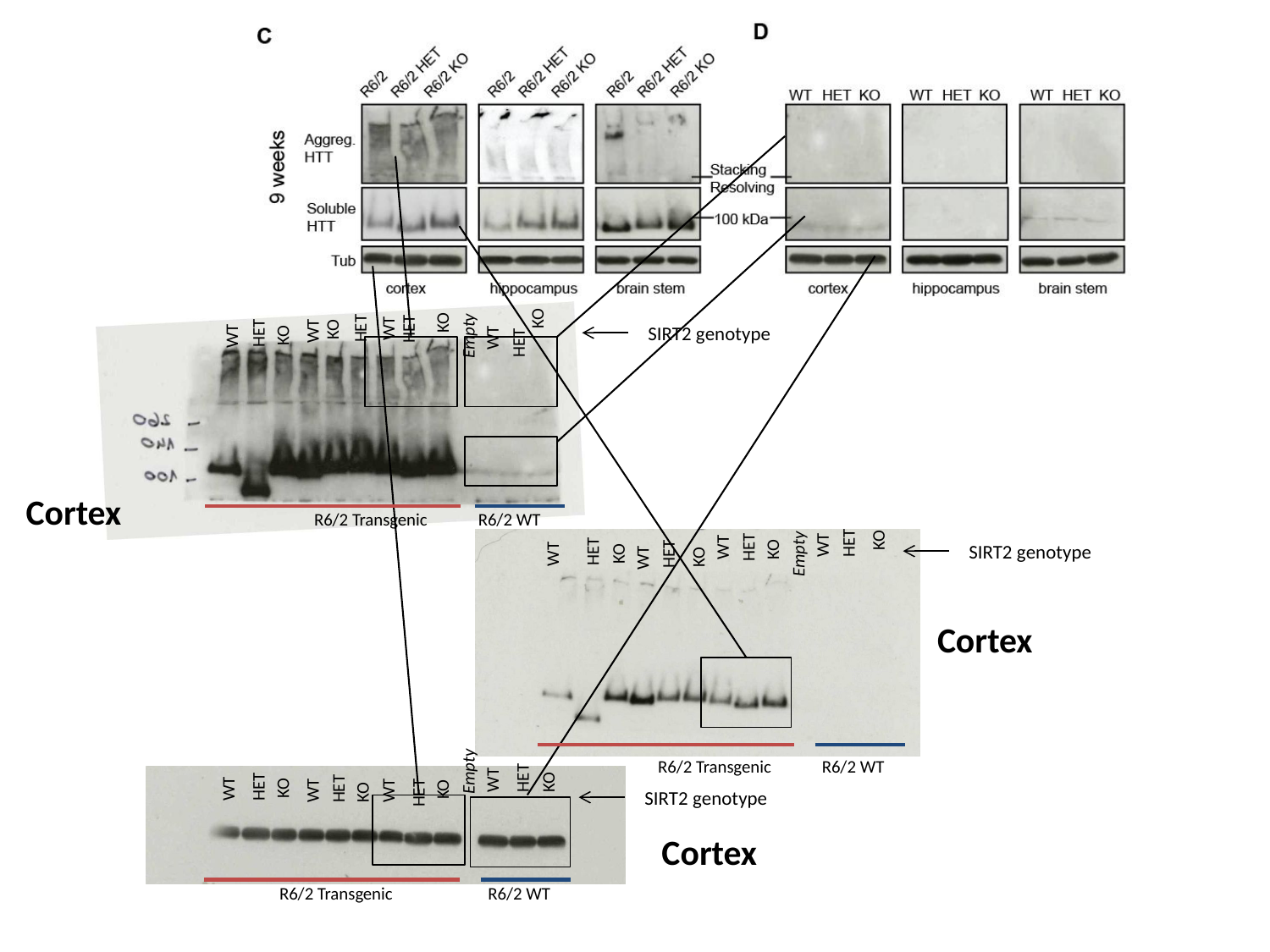

KO
KO
HET
WT
HET
KO
WT
SIRT2 genotype
HET
KO
WT
Empty
WT
HET
Cortex
R6/2 Transgenic
R6/2 WT
KO
HET
WT
WT
HET
KO
SIRT2 genotype
HET
KO
HET
WT
Empty
KO
WT
Cortex
R6/2 Transgenic
R6/2 WT
Empty
HET
WT
KO
HET
KO
HET
WT
KO
WT
WT
KO
HET
SIRT2 genotype
Cortex
R6/2 Transgenic
R6/2 WT

## Slide 5
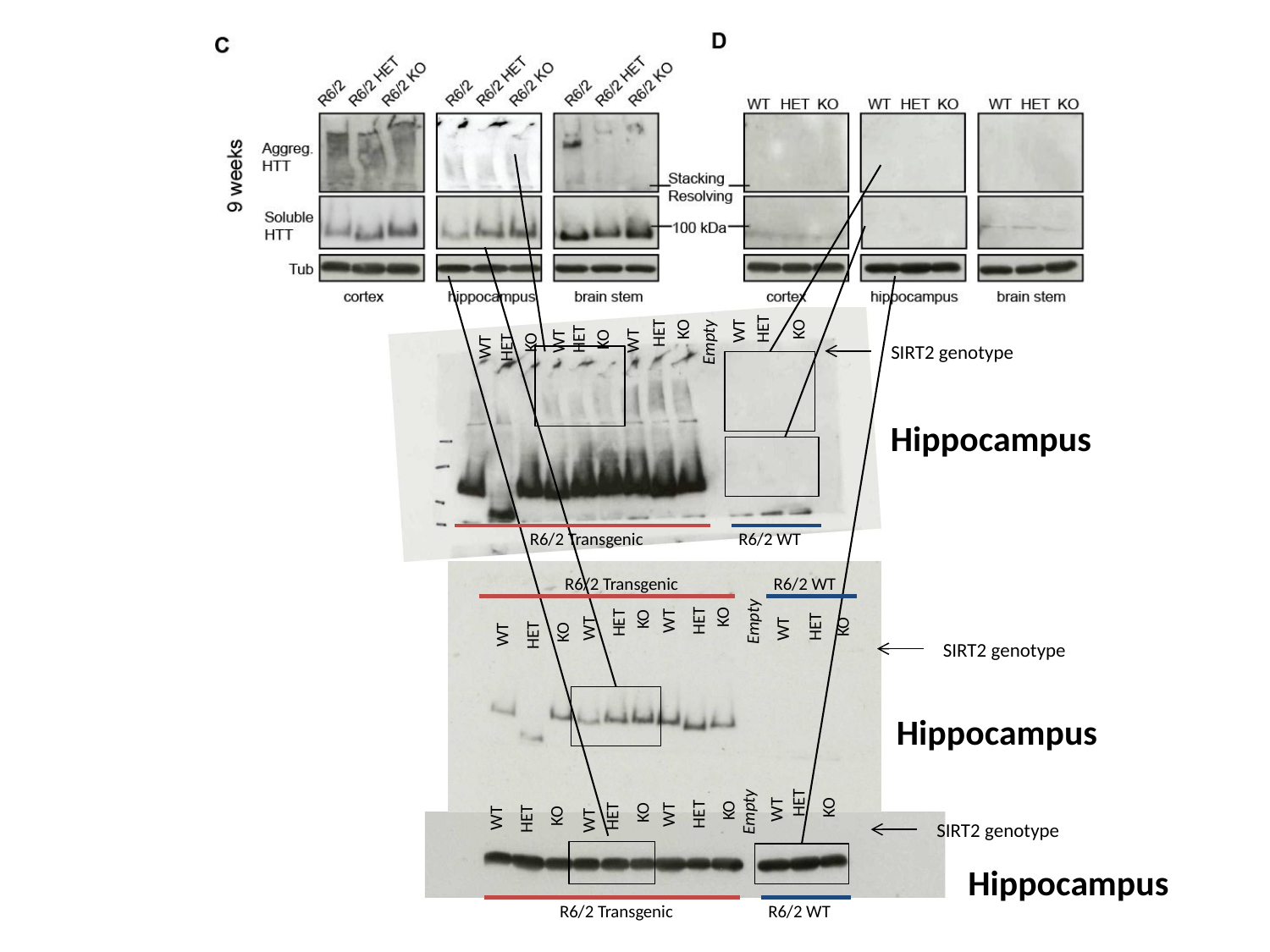

HET
KO
KO
WT
HET
HET
KO
WT
WT
Empty
KO
WT
HET
SIRT2 genotype
Hippocampus
R6/2 Transgenic
R6/2 WT
R6/2 Transgenic
R6/2 WT
KO
KO
HET
WT
Empty
HET
HET
KO
WT
WT
KO
WT
HET
SIRT2 genotype
Hippocampus
HET
KO
WT
KO
Empty
KO
HET
WT
KO
HET
WT
HET
WT
SIRT2 genotype
Hippocampus
R6/2 Transgenic
R6/2 WT

## Slide 6
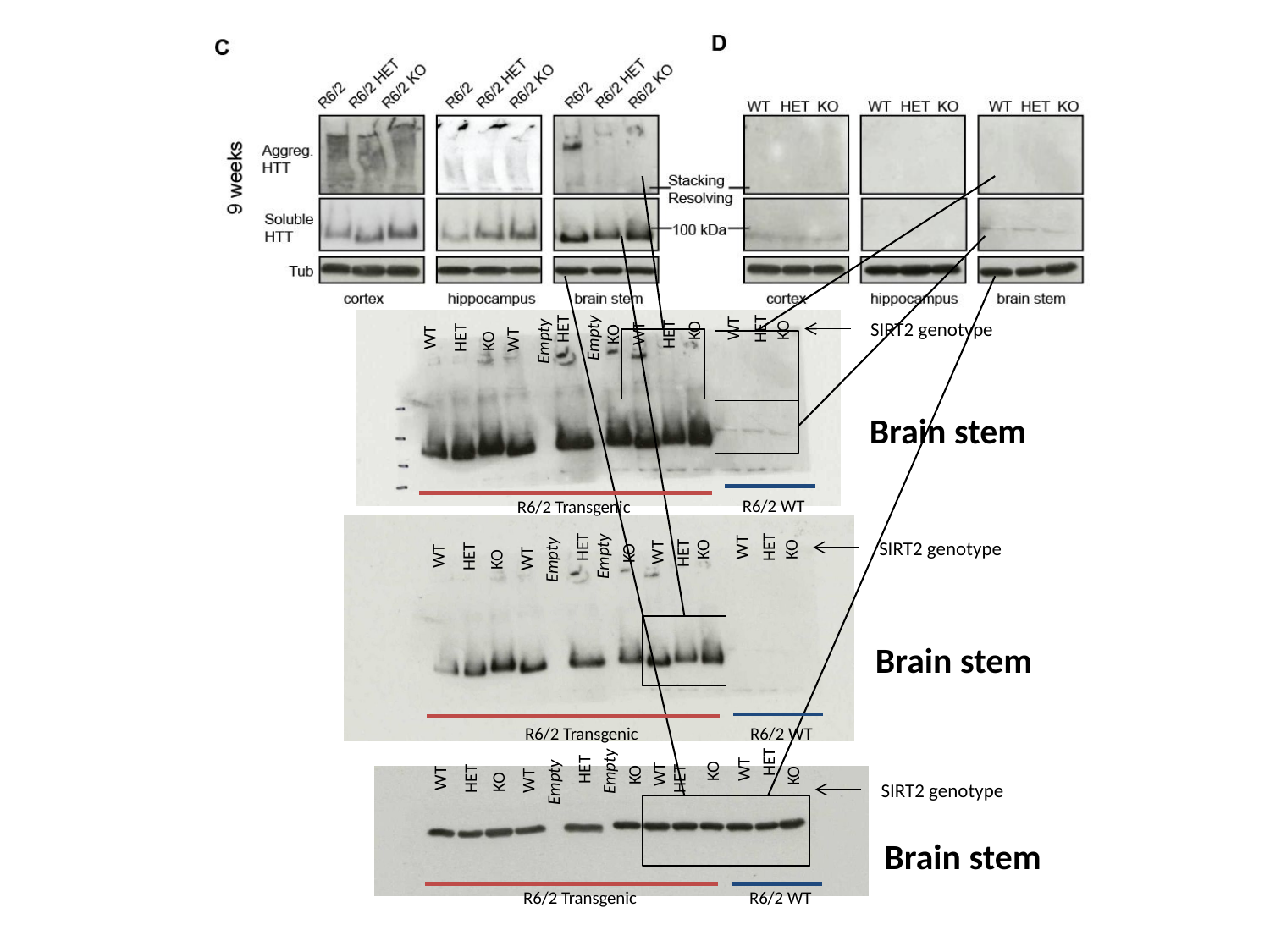

SIRT2 genotype
HET
HET
WT
KO
KO
KO
WT
HET
WT
Empty
HET
WT
Empty
KO
Brain stem
R6/2 WT
R6/2 Transgenic
SIRT2 genotype
HET
HET
WT
KO
KO
KO
WT
HET
WT
Empty
HET
WT
Empty
KO
Brain stem
R6/2 Transgenic
R6/2 WT
HET
HET
WT
KO
KO
Empty
WT
KO
WT
HET
HET
WT
Empty
KO
SIRT2 genotype
Brain stem
R6/2 Transgenic
R6/2 WT

## Slide 7
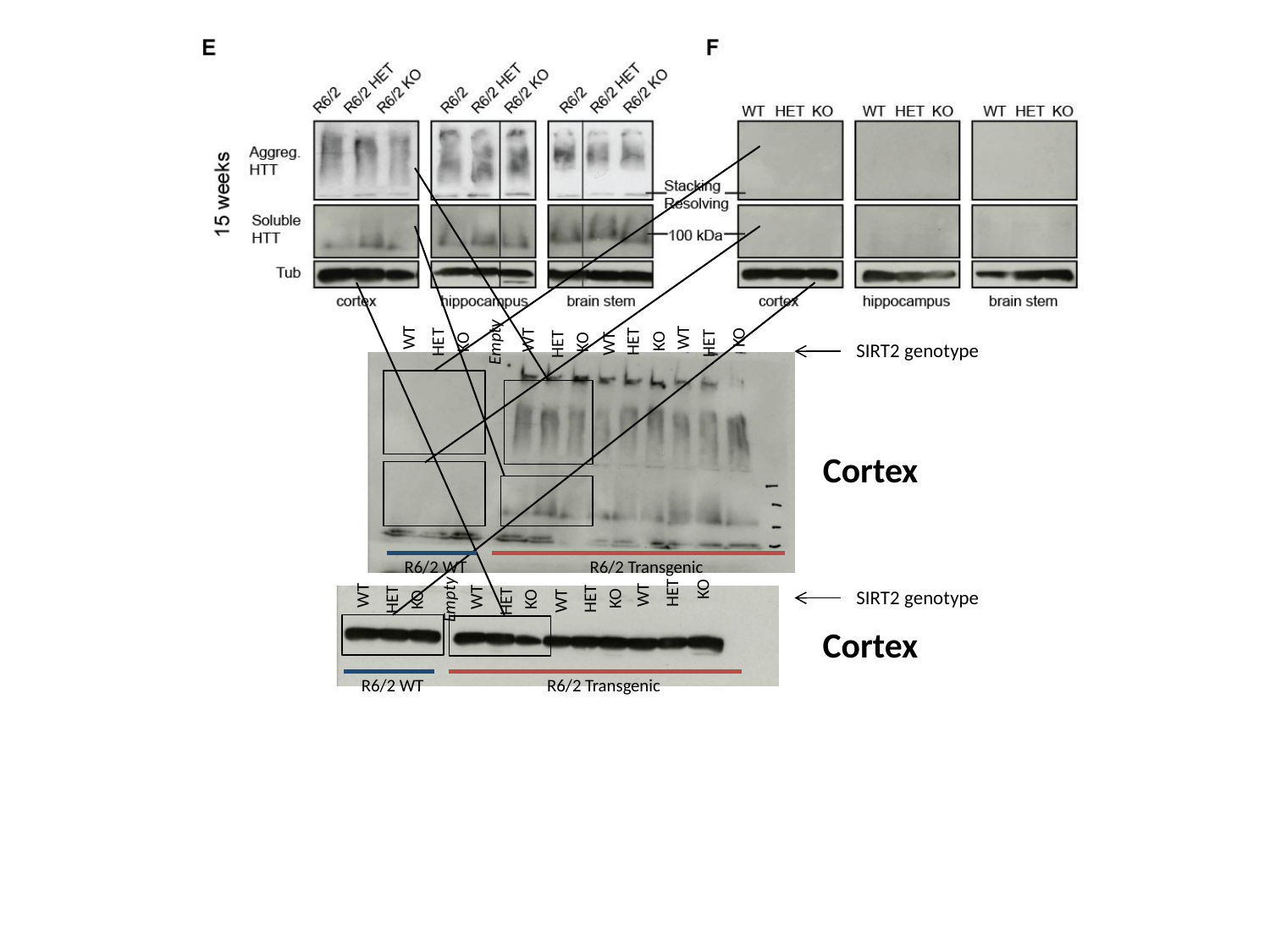

KO
WT
WT
WT
HET
KO
KO
HET
Empty
KO
HET
WT
HET
SIRT2 genotype
Cortex
R6/2 WT
R6/2 Transgenic
KO
HET
WT
WT
SIRT2 genotype
WT
HET
KO
KO
HET
Empty
KO
WT
HET
Cortex
R6/2 WT
R6/2 Transgenic

## Slide 8
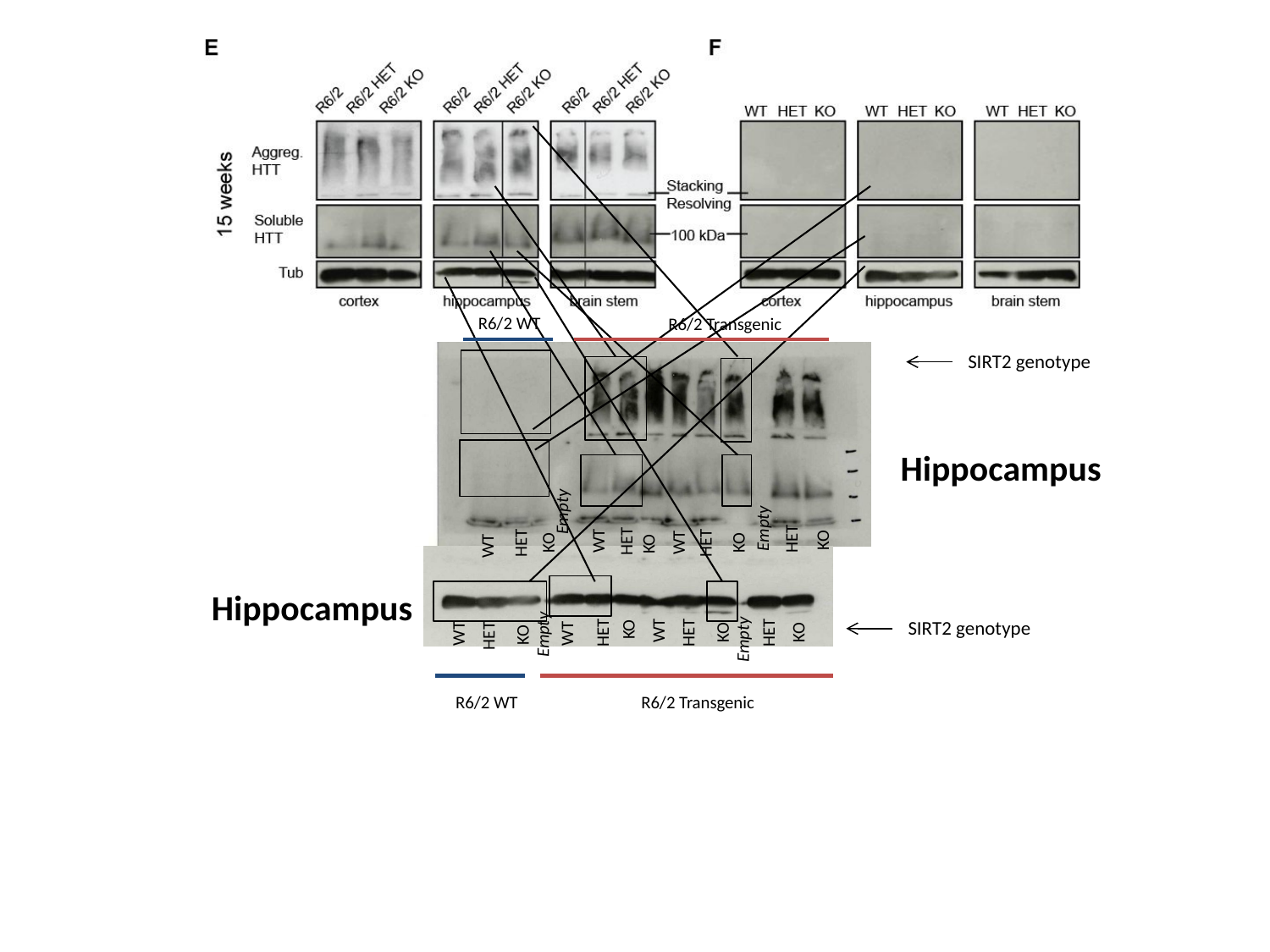

R6/2 WT
R6/2 Transgenic
SIRT2 genotype
Hippocampus
Empty
Empty
HET
KO
HET
WT
KO
WT
HET
KO
HET
KO
WT
Hippocampus
SIRT2 genotype
KO
WT
KO
KO
HET
HET
HET
WT
WT
Empty
KO
HET
Empty
R6/2 WT
R6/2 Transgenic

## Slide 9
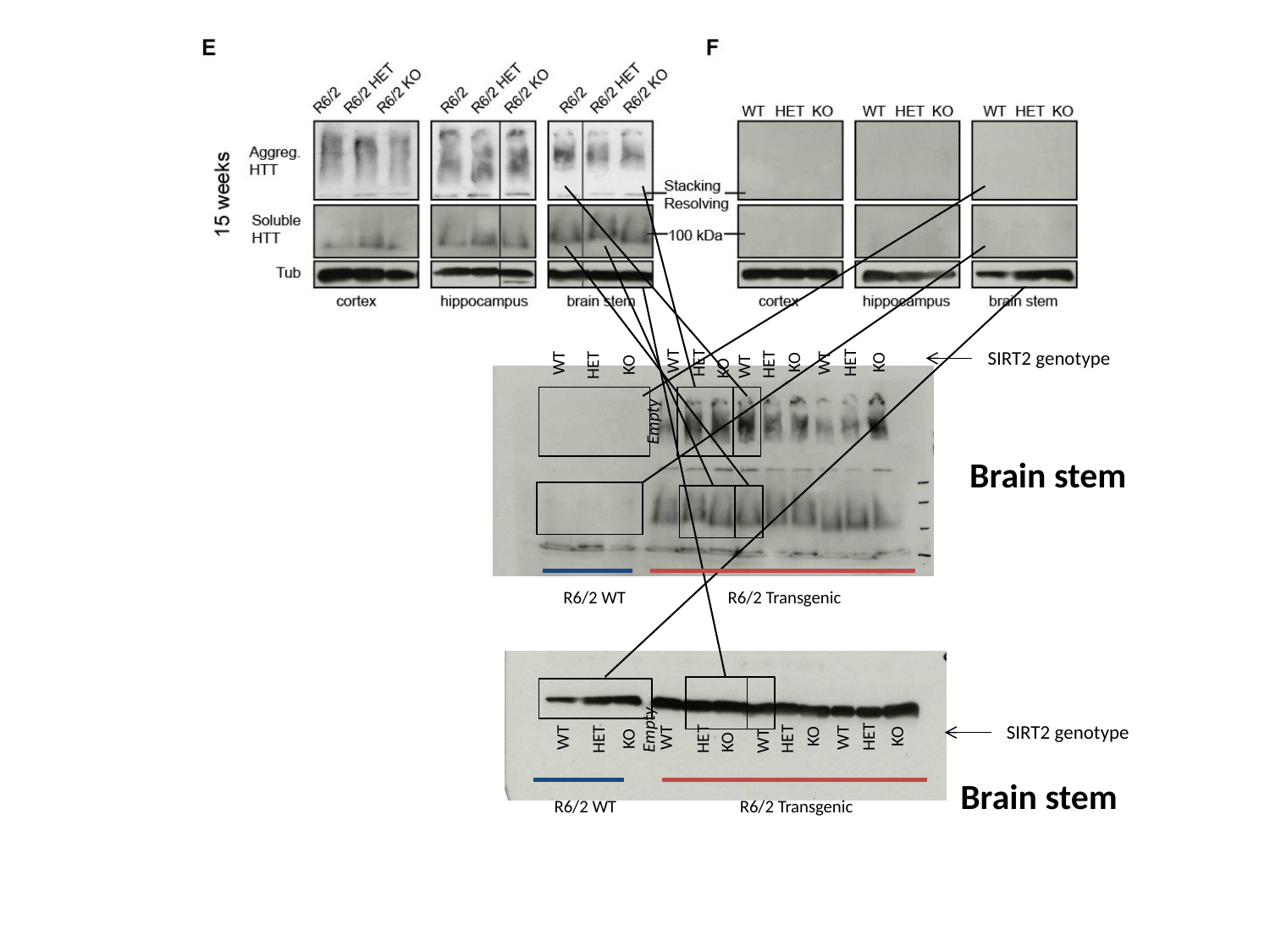

SIRT2 genotype
WT
KO
KO
HET
HET
WT
WT
HET
KO
HET
WT
KO
Empty
Brain stem
R6/2 WT
R6/2 Transgenic
Empty
SIRT2 genotype
KO
KO
HET
WT
WT
WT
HET
HET
KO
HET
WT
KO
Brain stem
R6/2 WT
R6/2 Transgenic
